# Supplementary material for: Trial Of Neurostimulation In Conversion Symptoms (TONICS): a feasibility randomised controlled trial of transcranial magnetic stimulation for functional limb weakness
Source: BMJ Open. 2020 Oct 6;10(10):e037198. doi: 10.1136/bmjopen-2020-037198 (PMC7539585; doi:10.1136/bmjopen-2020-037198)
Supplement: Supplementary data [file bmjopen-2020-037198supp001.pdf]

Supplementary File 1. Patient Information Sheet

*There is no potentially identifiable patient information in this document.

Patient Information Sheet & Consent Form

Trial Of Neurostimulation In Conversion Symptoms (TONICS):

A Randomised Controlled Trial (RCT) feasibility study of Transcranial Magnetic Stimulation (TMS) for conversion disorder with motor symptoms

REC reference number 17/LO/0410

You are being invited to take part in a research study. Before you decide, please take time to read the following information and to decide whether or not you would like to participate. If anything is unclear or you would like further information, please ask a member or the research team. Thank you for taking the time to read this.

What is the purpose of the study?

This study aims to assess Transcranial Magnetic Stimulation (TMS) as a new potential treatment for conversion disorder (CD), also known as Functional Neurological Disorder (FND). CD is where neurological symptoms, such as weakness, occur but no structural neurological disease can be found – therefore they are disorders of function, rather than structure. There are few proven treatments for weakness that is caused by CD. However, there is encouraging preliminary evidence that TMS could be an effective and safe treatment for such symptoms but until a Randomised Controlled Trial (RCT) is conducted it is not possible to establish whether this is the case.

What is Transcranial Magnetic Stimulation (TMS)?

TMS is a form of 'non-invasive brain stimulation', i.e. it is a way of stimulating the brain from outside the head. It works by holding a magnetic coil approximately the size of a small side plate against the head (it rests on the scalp) which then delivers magnetic pulses that stimulate the underlying brain. It was developed over 30 years ago and has been increasingly used treat a number of neurological and psychiatric disorders. It is considered to be a relatively safe and generally well-tolerated treatment. This is a picture of TMS coil being used in our laboratory*:

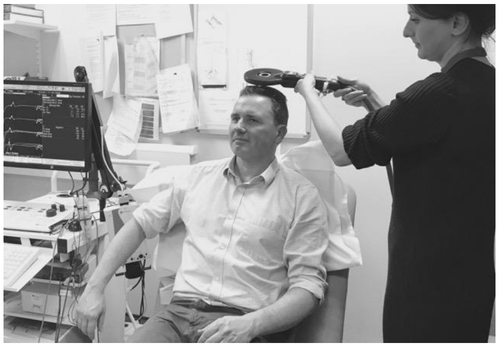

*The individuals depicted in this image are members of the research team, not clinical cases.

Pick S, *et al. BMJ Open* 2020; 10:e037198. doi: 10.1136/bmjopen-2020-037198

What is a Randomised Controlled Trial (RCT)?

Randomised Controlled Trials (RCTs) are the best way to tell whether a treatment really works and each year thousands of people take part in them. The word 'controlled' means that a 'control' treatment, e.g. an inactive or 'placebo' form of the treatment, is used to compare response to the 'active' treatment being investigated. This allows us to know whether any improvements (or side effects) are really due to the treatment or could either have occurred due to placebo effects or could have naturally occurred. Therefore patients are allocated to different groups to receive either the active treatment (Group A) or the inactive / placebo treatment (Group B).

The term 'randomised' means that people allocated at random to one of these two groups as this is the only way to compare treatments fairly. Randomisation means the chances are exactly equal for being allocated to either group and therefore no-one can predict in advance the group to which you will be allocated, in case this in any way affects what you or we expect to be the outcome of the study. Random allocation could be done using the result of tossing a coin (i.e. 'heads' for group A and 'tails' for Group B) to decide which treatment you will get but we will do this using a computer.

It is also important, where possible, that patients do no know (i.e. are 'blind' to) which treatment they have been allocated to as this can affect response. This means you won't know which group you've been allocated to until *after* you have not only completed the treatment but also completed the follow up interviews and questionnaires which will assess your response to the treatment you received.

For those who were allocated the 'inactive treatment' if it is felt after completing the treatment that they might benefit from receiving 'active' TMS as well, they will be offered this treatment after finishing the trial.

Why have I been chosen?

You have been chosen because you are over 18 and have been diagnosed with CD that is causing weakness in at least one of your limbs – this is known as 'motor' CD. As we don't currently know if TMS is any more helpful to patients than placebo, a Randomised Controlled Trial (RCT) is the most exact and fair way for us to see how helpful TMS really is at improving weakness in motor CD.

Do I have to take part and can I withdraw from the study if I change my mind?

It is completely up to you to decide whether or not to take part. You may consider this at your leisure, and contact us for more information, at the number below or arrange to discuss the study with a member of the research team. If you do decide to take part you will still be free to withdraw at any time and without giving a reason and this will not affect the standard of care you receive now or in the future. We would not collect any new information on you. However, any information that we had already collected would be kept by the study team.

What will happen to me if I take part?

If you decide to take part then a research worker will arrange to meet with you at a time that is convenient for you. At the appointment the research worker will explain the study to you in more detail, check you are eligible for the study and answer any questions that

you may have. We will give you another copy of this Information Sheet to keep and ask you to sign a consent form.

The research worker will then collect some simple information on things such as your age, previous medical history, current medications and employment history. They will undertake an assessment of any psychological problems that you may have and ask you to complete a number of questionnaires. In total this will take about 1.5 hours. and will explain how treatment might help you. They will also carefully check that it is safe to give you TMS treatment, such as whether you have seizures (specifically epileptic seizures).

You will then be randomly assigned to either Group A, where you will receive the active treatment, or to Group B where you will receive the inactive treatment. The randomisation will be done by someone who does not know you and who is not directly involved in the study.

You will then be invited for the first treatments session. The treatment itself will take about 30 minutes and beforehand your strength will be tested by a member of the research team and you will be asked to fill in some more questionnaires about your current symptoms and health which will take approximately another 60 minutes so the whole session will take about an hour and a half. You will then be invited back for another identical treatment session 1 month later. Another 2 months later, so 3 months after the first session, you will be invited for a final session – this time with no treatment but just the examination and questionnaires. All these sessions will be arranged at a time to suit you and we will provide your transport costs.

How long will I be in the study?

If you agree to take part in this part of the study it will take 3 months from the start of treatment until the completion of the last follow up session.

What are the possible risks of taking part?

There are some risks to taking part in the study as TMS can cause side effects. The most common side effect is that some people can find the TMS treatment uncomfortable around the area it is delivered to (the scalp) and for some this experience is painful but the vast majority of people given the type of TMS in this study find it tolerable.

It can also cause headaches which generally resolve soon after the treatment is given. Very rarely it can cause seizures – but is only reported to occur with higher ‘doses’ of TMS than used in this study and only in those with, or predisposed to, epilepsy - which is why this is carefully screened for beforehand.

It is also possible that some of the questionnaires you will be asked to fill might cause you distress to answer as they ask about you past psychiatric history and if you have suffered from any abuse. If you experience any of these issues you can discuss them with a member of the research team or your GP and re-evaluate whether you want to continue with the study or not.

What are the possible benefits of taking part?

By taking part in the study you will help us understand more about treatments that are effective in helping people with weakness caused by CD. We cannot be sure at this stage whether the active TMS will be any more effective than the inactive TMS and

Pick S, *et al. BMJ Open* 2020; 10:e037198. doi: 10.1136/bmjopen-2020-037198

therefore whether you will personally benefit, regardless of which group you are allocated to.

Will taking part or not influence my medical care?

Your participation will have no influence on your medical care. There will be no restrictions on your diet or lifestyle during the study. Any doctors or other healthcare professionals you see can make any changes to your medication or other treatments that they feel are necessary for you. Similarly, as mentioned above, not taking part will have no influence on any aspect of your care.

What expenses will be covered?

Whichever group you are allocated to, we will pay for your travel up to a maximum £25 for each assessment that is necessary. However, if you take time off work to attend the study appointments we cannot pay you or your employer for this.

Will my taking part in this study be kept confidential?

All information which is collected about you during the course of the research will be kept strictly confidential. The research workers who contact you will need to keep your contact details at the university research sites, but only for the purposes of contacting you about arranging to see you or to send you questionnaires. Any other information about you will have your name and address removed so that you cannot be recognised from it. We will not identify you in our computers or publications by name, and will only refer to you by participant number, which will be used in place of your name on any future publications. All information will be stored on password protected computers and paperwork will be stored securely in locked university offices.

If you take part in this Randomised Controlled Trial (RCT) we will ask if we can contact you, perhaps through your GP, if you move house during our study. With your permission we would want to inform your GP that you are taking part in the study and potentially also see your medical file. We would also need to inform your GP or other professionals if one of the health professionals or research workers in the study became concerned about your well-being or about the implications of what you tell us for someone else’s well-being. We would of course discuss this with you if such a situation arose.

What will happen if new information becomes available?

Sometimes during the course of a study new information might become available about the treatment that is being tested. If this happens, either your medical doctor or a member of the research team will contact you and arrange to talk to you about this and discuss with you whether you want to continue. If you decide to withdraw from the study your doctor will make arrangements for your care to continue. If you decide to continue in the study you may be asked to sign an updated consent form.

What happens when the trial is over?

Once the trial is over, we will see whether the active TMS has helped people reduce their weakness any more than the inactive TMS. If you did not receive active TMS during the study then the doctors treating you will decide whether you might still benefit from this and if so they will refer you for this treatment.

What happens if something goes wrong?

We do not expect there to be any significant adverse effects from taking part in this study. However if you are harmed during the study and this is due to someone's negligence, then you may have grounds for legal action for compensation against the NHS but you may have to pay your legal costs. Regardless of this, if you wish to complain, or have any concerns about any aspect of the way you have been approached or treated during the course of this study, the normal National Health Service complaints mechanisms should be available to you.

King's College London holds insurance policies that apply to this study. If you experience harm or injury as a result of taking part in this study you may be eligible to claim compensation without having to prove that King's College London is at fault. This does not affect your legal rights to seek compensation.

If for any reason your symptoms get much worse during the study, then you will be able to talk to your medical specialist or any of us who are involved in the study and discuss what you want to do.

What happens to the results of the research study?

We will publish the results of the research in scientific journals and we will present the results at scientific meetings. In addition we will talk to service providers about the results of our research. We will not identify you in any report/publication. If you would like a copy of the published results, we can provide this at the end of the study.

How often will I be contacted by the investigators?

We will need to contact you at different stages of the study to arrange treatment or follow up sessions and will give you two reminders to let us have this information. If at any particular stage you change your mind about taking part in the study and we do not hear from you at all, we will contact you on only one further occasion to discuss the study. If we cannot discuss this with you we will assume you have chosen to leave the study. We can reassure you that you will not be contacted repeatedly if you decide you no longer wish to be part of the study. If you then change your mind about letting us have the information we asked for, you can contact us by phone, letter or email to then re-join the study if you wish.

Can my participation in the study be discontinued by the investigators?

Yes. At any time during the study, the investigators have the right to end your participation in the study for any reason. If so, this reason will be explained to you. If later on in the study it is concluded that you no longer have capacity to consent to participating we would like to be able to continue to use any data that we have already collected, in an anonymised form.

Who is organized, funded and reviewed the research?

The research is funded by the National Institute of Health Research, and administered by the Institute of Psychiatry Psychology & Neuroscience, part of King's College London. The study has been reviewed and approved by a UK Research Ethics Committee (London-Stanmore Research Ethics Committee - study reference number 17/LO/0410).

Contacts for Further Information

If you require any further information, please contact Dr Nicholson or a member of the research team at the Section of Cognitive Neuropsychiatry (PO68), Institute of Psychiatry Psychology & Neuroscience, De Crespigny Park, London SE5 8AF
Tel: 0207 848 5136 Fax: 0207 848 0572 Email timothy.nicholson@kcl.ac.uk.

You will be given a copy of this information sheet and a signed consent form to keep.

If you would like any independent advice about taking part in a research study, or have concerns about the conduct of the study, please contact your Trust Patient Advice and Liaison Service (PALS). PALS offers free confidential advice, support and information on health-related matters and are independent of clinical services. They provide a point of contact for patients, their families and their carers. PALS also helps to improve the NHS by listening to your concerns and suggestions. You can find your nearest PALS on the NHS Choices website: http://www.nhs.uk/Service-Search/Patient-advice-and-liaison-services-(PALS)/LocationSearch/363

Local PALS offices are also listed below:

South London & Maudsley NHS Foundation Trust (SLAM) PALS
Website: http://www.slam.nhs.uk/patients-and-carers/advice-and-information
Email: pals@slam.nhs.uk
Phone: 0800 731 2864 (freephone number)

King's College Hospital NHS Foundation Trust (KCH) PALS
Online contact form: https://www.kch.nhs.uk/contact/pals
Email: kch-tr.PALS@nhs.net
Phone: 020 3299 3601, 9am to 4.30pm, Monday to Friday (not bank holidays)

Guys and St Thomas' NHS Foundation Trust (GST) PALS
Online contact form: http://www.guysandstthomas.nhs.uk/contact-us/feedback-forms/Questions-about-care.aspx
Email: pals@gstt.nhs.uk
Phone: 020 7188 8801

Pick S, *et al. BMJ Open* 2020; 10:e037198. doi: 10.1136/bmjopen-2020-037198
