# Supplementary material for: Trial Of Neurostimulation In Conversion Symptoms (TONICS): a feasibility randomised controlled trial of transcranial magnetic stimulation for functional limb weakness
Source: BMJ Open. 2020 Oct 6;10(10):e037198. doi: 10.1136/bmjopen-2020-037198 (PMC7539585; doi:10.1136/bmjopen-2020-037198)
Supplement: Supplementary data [file bmjopen-2020-037198supp002.pdf]

Supplementary File 2

Table 2.1. Concomitant treatments by group and timepoint*

Treatment	Baseline	TMS Visit 1<br>n (%)	TMS Visit 2<br>n (%)	Follow up<br>n (%)
**Medication**	Active=10 (100)<br>Inactive=10 (100)	Active=10 (100)<br>Inactive=9 (100)	Active=6 (100)<br>Inactive=8 (100)	Active=9 (100)<br>Inactive=9 (100)
**Physiotherapy**	Active=4 (40)<br>Inactive=2 (20)	Active=4 (40)<br>Inactive=2 (2)	Active=0 (0)<br>Inactive=1 (13)	Active=1 (11)<br>Inactive=1 (11)
**Neurology inpatient**	Active=0 (0)<br>Inactive=0 (0)	Active=1 (10)<br>Inactive=0 (0)	Active=0 (0)<br>Inactive=0 (0)	Active=0 (0)<br>Inactive=0 (0)
**General inpatient**	Active=3 (30)<br>Inactive=1 (10)	Active=2 (20)<br>Inactive=1 (11)	Active=0 (0)<br>Inactive=1 (13)	Active=0 (0)<br>Inactive=0 (0)
**Specialist MDT inpatient neurorehabilitation**	Active=1 (10)<br>Inactive=1 (10)	Active=1 (10)<br>Inactive=1 (11)	Active=1 (17)<br>Inactive=1 (13)	Active=1 (11)<br>Inactive=1 (11)
**Specialist MDT day hospital**	Active=0 (0)<br>Inactive=1 (10)	Active=0 (0)<br>Inactive=1 (11)	Active=0 (0)<br>Inactive=0 (0)	Active=1 (11)<br>Inactive=0 (0)
**CBT / Psychology**	Active=2 (20)<br>Inactive=1 (10)	Active=2 (20)<br>Inactive=1 (11)	Active=1 (17)<br>Inactive=0 (0)	Active=1 (11)<br>Inactive=2 (22)
**Occupational therapy**	Active=2 (20)<br>Inactive=1 (10)	Active=2 (20)<br>Inactive=1 (11)	Active=1 (17)<br>Inactive=1 (13)	Active=1 (11)<br>Inactive=0 (0)
**Psychiatry (outpatient)**	Active=0 (0)<br>Inactive=0 (0)	Active=0 (0)<br>Inactive=0 (0)	Active=0 (0)<br>Inactive=1 (13)	Active=1 (11)<br>Inactive=1 (11)

Key: CBT=cognitive behavioural therapy; MDT=multidisciplinary team; TMS=transcranial magnetic stimulation
