# Supplementary material for: Trial Of Neurostimulation In Conversion Symptoms (TONICS): a feasibility randomised controlled trial of transcranial magnetic stimulation for functional limb weakness
Source: BMJ Open. 2020 Oct 6;10(10):e037198. doi: 10.1136/bmjopen-2020-037198 (PMC7539585; doi:10.1136/bmjopen-2020-037198)
Supplement: Supplementary data [file bmjopen-2020-037198supp003.pdf]

Supplementary File 3 – Outcome measure completion data

Table 3.1. Data quality by timepoint*

Outcome measure	TMS Visit 1 n (%)	TMS Visit 2 n (%)	Follow up n (%)
**CGI Patient**	21 (100)	16 (100)	19 (100)
**CGI Outcome assessor**	21 (100)	16 (100)	20 (105)
**CGI Carer**	2 (10)	4 (25)	4 (21)
**SF36: Physical Function**	21 (100)	16 (100)	19 (100)
**SF36: Role Physical**	20 (95)	16 (100)	19 (100)
**SF36: Bodily Pain**	21 (100)	16 (100)	19 (100)
**SF36: General Health**	21 (100)	16 (100)	19 (100)
**SF36: Vitality**	21 (100)	16 (100)	19 (100)
**SF36: Social Functioning**	21 (100)	16 (100)	19 (100)
**SF36: Role Emotional**	18 (86)	16 (100)	19 (100)
**SF36: Mental Health**	21 (100)	16 (100)	19 (100)
**Barthel Index**	21 (100)	16 (100)	20 (105)
**FIM-FAM**	4 (19)	2 (12)	2 (11)
**GAD 7**	21 (100)	16 (100)	19 (100)
**PHQ 9**	21 (100)	16 (100)	19 (100)
**PHQ 15**	21 (100)	16 (100)	19 (100)
**CORE-10**	21 (100)	16 (100)	19 (100)
**WSAS**	21 (100)	16 (100)	19 (100)
**Left Arm; Strength**	20 (95)	15 (94)	17 (89)
**Left Arm: Weakness**	20 (95)	15 (94)	18 (95)
**Right Arm: Strength**	20 (95)	15 (94)	17 (89)
**Right Arm: Weakness**	20 (95)	15 (94)	18 (95)
**Left Leg; Strength**	21 (100)	16 (100)	18 (95)
**Left Leg: Weakness**	21 (100)	16 (100)	19 (100)
**Right Leg: Strength**	20 (95)	15 (94)	17 (89)
**Right Leg: Weakness**	20 (95)	15 (94)	18 (95)
**Dynamometry Left Arm: Max**	20 (95)	15 (94)	17 (89)
**Dynamometry Left Arm: Max**	20 (95)	15 (94)	18 (95)
**Dynamometry Left Arm: Max**	20 (95)	15 (94)	17 (89)
**Dynamometry Left Arm: Max**	20 (95)	15 (94)	18 (95)

Key: CGI=Clinical Global Impression; CORE=10=Clinical Outcomes in Routine Evaluation-10 item; GAD-7=Generalised Anxiety Disorder-7 item; KG=kilogram; PHQ=Patient Health Questionnaire; SF-36=Short Form Health Survey-36 item; TMS=transcranial magnetic stimulation; WSAS=Work & Social Adjustment Scale

**Percentages calculated relative to the number of patients in attendance in each group*
