# Supplementary material for: Trial Of Neurostimulation In Conversion Symptoms (TONICS): a feasibility randomised controlled trial of transcranial magnetic stimulation for functional limb weakness
Source: BMJ Open. 2020 Oct 6;10(10):e037198. doi: 10.1136/bmjopen-2020-037198 (PMC7539585; doi:10.1136/bmjopen-2020-037198)
Supplement: Supplementary data [file bmjopen-2020-037198supp004.pdf]

Supplementary File 4 - Descriptive statistics and effect sizes for primary and secondary outcomes

Supplementary Table 4.1. Patient CGI-I ratings

		Visit 1				Visit 2				Follow-up	
		Pre-TMS		Post-TMS		Pre-TMS		Post-TMS			
		Active	Inactive	Active	Inactive	Active	Inactive	Active	Inactive	Active	Inactive
**Very much improved**	**n (%)**	0 (0)	0 (0)	0 (0)	0 (0)	0 (0)	1 (10)	0 (0)	0 (0)	0 (0)	0 (0)
**Much improved**	**n (%)**	0 (0)	1 (9)	0 (0)	1 (9)	4 (67)	1 (10)	4 (67)	2 (20)	4 (44)	2 (20)
**Minimally improved**	**n (%)**	1 (10)	2 (18)	1 (10)	2 (18)	0 (0)	1 (10)	0 (0)	1 (10)	1 (11)	4 (40)
**No change**	**n (%)**	3 (30)	5 (45)	4 (40)	6 (55)	0 (0)	2 (20)	0 (0)	2 (20)	2 (22)	1 (10)
**Minimally worse**	**n (%)**	3 (30)	1 (9)	2 (20)	0 (0)	1 (17)	3 (30)	1 (17)	2 (20)	1 (11)	2 (20)
**Much worse**	**n (%)**	3 (30)	2 (18)	3 (30)	2 (18)	1 (17)	2 (20)	1 (17)	3 (30)	0 (0)	1 (10)
**Very much worse**	**n (%)**	0 (0)	0 (0)	0 (0)	0 (0)	0 (0)	0 (0)	0 (0)	0 (0)	1 (11)	0 (0)
**Total**	**n (%)**	10 (100)	11 (100)	10(100)	11 (100)	6 (60)	10 (91)	6 (60)	10 (91)	9 (90)	10 (91)
**Missing***	**n (%)**	0 (0)	0 (0)	0 (0)	0 (0)	4 (40)	1 (9)	4 (40)	1 (9)	1 (10)	1 (9)
**Effect size (negative = benefit)**	**Cliff's delta (95% CI)**	0.35 (-0.17, 0.71)		0.35 (-0.15, 0.7)		-0.35 (-0.73, 0.19)		-0.44 (-0.79, 0.13)		-0.2 (-0.6, 0.28)	

Key: CGI-I=Clinical Global Impression-Improvement; CI=confidence interval; TMS=transcranial magnetic stimulation

**Percentage calculated relative to total number of participants enrolled in study*

Supplementary Table 4.2. Outcome assessor CGI-I ratings

		Visit 1				Visit 2				Follow-up	
		Pre-TMS		Post-TMS		Pre-TMS		Post-TMS			
		**Active**	**Inactive**	**Active**	**Inactive**	**Active**	**Inactive**	**Active**	**Inactive**	**Active**	**Inactive**
**Very much improved**	**n (%)**	0 (0)	0 (0)	0 (0)	0 (0)	0 (0)	0 (0)	0 (0)	0 (0)	0 (0)	0 (0)
**Much improved**	**n (%)**	0 (0)	0 (0)	0 (0)	0 (0)	3 (50)	2 (20)	3 (50)	2 (20)	4 (44)	2 (18)
**Minimally improved**	**n (%)**	1 (10)	3 (27)	1 (10)	3 (27)	1 (17)	1 (10)	1 (17)	1 (10)	3 (33)	5 (45)
**No change**	**n (%)**	3 (30)	7 (64)	4 (40)	6 (55)	0 (0)	5 (50)	0 (0)	5 (50)	1 (11)	2 (18)
**Minimally worse**	**n (%)**	3 (30)	1 (9)	1 (10)	2 (18)	2 (33)	2 (20)	2 (33)	2 (20)	0 (0)	1 (9)
**Much worse**	**n (%)**	3 (30)	0 (0)	4 (40)	0 (0)	0 (0)	0 (0)	0 (0)	0 (0)	0 (0)	1 (9)
**Very much worse**	**n (%)**	0 (0)	0 (0)	0 (0)	0 (0)	0 (0)	0 (0)	0 (0)	0 (0)	1 (11)	0 (0)
**Total**	**n (%)**	10 (100)	11 (100)	10 (100)	11 (100)	6 (60)	10 (91)	6 (60)	10 (91)	9 (90)	11 (100)
**Missing***	**n (%)**	0 (0)	0 (0)	0 (0)	0 (0)	4 (40)	1 (9)	4 (40)	1 (9)	1 (10)	0 (0)
**Effect size (negative = benefit)**	**Cliff's delta (95% CI)**	0.55 (0.05, 0.83)		0.45 (-0.06, 0.77)		-0.29 (-0.69, 0.25)		-0.29 (-0.69, 0.25)		-0.26 (-0.65, 0.23)	

Key: CGI-I=Clinical Global Impression-Improvement; CI=confidence interval; TMS=transcranial magnetic stimulation

**Percentage calculated relative to total number of participants enrolled in the study*

Supplementary Table 4.3. Patient weakness ratings

		Visit 1				Visit 2				Follow-up	
		Pre-TMS		Post-TMS		Pre-TMS		Post-TMS			
		**Active**	**Inactive**	**Active**	**Inactive**	**Active**	**Inactive**	**Active**	**Inactive**	**Active**	**Inactive**
**No weakness**	**n(%)**	2 (20)	1 (9)	3 (30)	1 (9)	0 (0)	1 (10)	1 (17)	1 (10)	2 (22)	0 (0)
**Mild weakness**	**n(%)**	1 (10)	3 (27)	0 (0)	5 (45)	1 (17)	4 (40)	0 (0)	5 (50)	1 (11)	5 (50)
**Moderate weakness**	**n(%)**	1 (10)	3 (27)	1 (10)	0 (0)	0 (0)	1 (10)	1 (17)	0 (0)	3 (33)	1 (10)
**Severe weakness**	**n(%)**	3 (30)	1 (9)	3 (30)	2 (18)	3 (50)	2 (20)	3 (50)	3 (30)	1 (11)	3 (30)
**Very severe weakness**	**n(%)**	3 (30)	3 (27)	3 (30)	3 (27)	2 (33)	2 (20)	1 (17)	1 (10)	2 (22)	1 (10)
**Total**	**n(%)**	10 (100)	11 (100)	10 (100)	11 (100)	6 (60)	10 (91)	6 (60)	10 (91)	9 (90)	10 (91)
**Missing***	**n(%)**	0 (0)	0 (0)	0 (0)	0 (0)	4 (40)	1 (9)	4 (40)	1 (9)	1 (10)	1 (9)
**Effect size (negative = treatment benefit)**	**Cliff's Delta (95% CI)**	0.09 (-0.41, 0.55)		0.04 (-0.46, 0.51)		0.27 (-0.11, 0.58)		0.17 (-0.25, 0.53)		-0.08 (-0.51, 0.37)	

Key: CI=confidence interval; TMS=transcranial magnetic stimulation

******Percentage calculated relative to total number of participants enrolled in the study*

Supplementary Table 4.4. Additional secondary outcome measures

		Visit 1		Visit 2		Follow-up
**Measure**	**Statistic**	**Pre-TMS**	**Post-TMS**	**Pre-TMS**	**Post-TMS**	
**Target limb strength rating (0-100%)**	**Mean (SD)**	Active=42.5 (37.4)<br>Inactive=52.3 (30.4)	Active=44.5 (40.6)<br>Inactive=52.7 (35)	Active=38.3 (25)<br>Inactive=55 (34)	Active=42.8 (34.1)<br>Inactive=57 (34)	Active=41.9 (27.5)<br>Inactive=51.8 (36.2)
	**Cohen's d**	0.29 (-0.63, 1.21)	0.22 (-0.7, 1.14)	0.54 (-0.59, 1.66)	0.42 (-0.7, 1.53)	0.3 (-0.71, 1.31)
**Dynamometry – left arm (average KG)**	**Mean (SD)**	Active=12.4 (10.8)<br>Inactive=6.1 (6.9)	Active=11.3 (11.7)<br>Inactive=7 (8.9)	Active=11.9 (3.7)<br>Inactive=6.3 (11)	Active=11.6 (6.1)<br>Inactive=6.4 (12)	Active=10.7 (9.1)<br>Inactive=9.7 (12.3)
	**Cohen's d**	0.68 (-0.35, 1.71)	0.41 (-0.61, 1.42)	0.65 (-0.6, 1.91)	0.53 (-0.72, 1.77)	0.09 (-1.02, 1.21)
**Dynamometry – right arm (average KG)**	**Mean (SD)**	Active=9.4 (9)<br>Inactive=10.5 (9.1)	Active=9.4 (8.6)<br>Inactive=9.6 (8.8)	Active=11.9 (6.6)<br>Inactive=10.3 (9.1)	Active=11.9 (9)<br>Inactive=9.6 (12.2)	Active=12.5 (12.9)<br>Inactive=11.1 (9.1)
	**Cohen's d**	-0.12 (-1.09, 0.85)	-0.02 (-0.99, 0.95)	0.19 (-0.99, 1.37)	0.21 (-0.97, 1.39)	0.13 (-0.95, 1.2)
**PHQ-15**	**Mean (SD)**	Active=15.4 (3.3)<br>Inactive=13.5 (6)		Active=15.7 (4.4)<br>Inactive=14.2 (7.2)		Active=15.2 (5.3)<br>Inactive=12.4 (6)
	**Cohen's d**	-0.39 (-1.31, 0.54)		-0.26 (-1.38, 0.85)		-0.5 (-1.48, 0.49)
**PHQ-9**	**Mean (SD)**	Active=15 (5.2)<br>Inactive=14.1 (8.9)		Active=13.3 (2.2)<br>Inactive=12.8 (8.4)		Active=14.3 (6.1)<br>Inactive=12.3 (11.2)
	**Cohen's d**	-0.13 (-1.04, 0.79)		-0.1 (-1.21, 1.01)		-0.22 (-1.19, 0.75)
**GAD-7**	**Mean (SD)**	Active=8.7 (5.6)<br>Inactive=10.5 (7.7)		Active=7.3 (3.4)<br>Inactive=7.5 (7)		Active=7.1 (4.9)<br>Inactive=9.1 (7.6)
	**Cohen's d**	0.28 (-0.64, 1.2)		0.03 (-1.07, 1.14)		0.32 (-0.66, 1.29)

Measure	Statistic	Visit 1 Pre-TMS	Visit 1 Post-TMS	Visit 2 Pre-TMS	Visit 2 Post-TMS	Follow-up
**CORE-10**	**Mean (SD)**	Active=18.4 (8.3)<br>Inactive=17.1 (10.3)		Active=16.7 (4)<br>Inactive=16.5 (9.4)		Active=14.8 (5.2)<br>Inactive=16.4 (8.2)
	**Cohen's d**	-0.14 (-1.06, 0.77)		-0.03 (-1.13, 1.08)		0.24 (-0.73, 1.21)
**SF-36 Physical functioning**	**Mean (SD)**	Active=10 (11.5)<br>Inactive=22.7 (22.2)		Active=15.8 (21.3)<br>Inactive=30 (28.9)		Active=21.2 (26.4)<br>Inactive=28 (29.6)
	**Cohen's d**	0.73 (-0.21, 1.68)		0.58 (-0.55, 1.71)		0.24 (-0.73, 1.22)
**SF-36 Physical role**	**Mean (SD)**	Active=2.5 (7.9)<br>Inactive=15 (33.7)		Active=4.2 (10.2)<br>Inactive=20 (36.9)		Active=8.3 (25)<br>Inactive=17.5 (37.4)
	**Cohen's d**	0.51 (-0.44, 1.46)		0.67 (-0.46, 1.81)		0.29 (-0.68, 1.27)
**SF-36 Bodily pain**	**Mean (SD)**	Active=22.2 (18.3)<br>Inactive=25 (27.1)		Active=29.8 (27.7)<br>Inactive=19.1 (22.1)		Active=31 (23.6)<br>Inactive=32.6 (21)
	**Cohen's d**	0.12 (-0.79, 1.04)		-0.42 (-1.53, 0.7)		0.07 (-0.9, 1.04)
**SF-36 General health**	**Mean (SD)**	Active=29.9 (9.7)<br>Inactive=30.8 (21.2)		Active=38.2 (15.8)<br>Inactive=35.4 (26.2)		Active=31.6 (11)<br>Inactive=39.8 (20.2)
	**Cohen's d**	0.06 (-0.86, 0.97)		-0.14 (-1.25, 0.97)		0.51 (-0.47, 1.5)
**SF-36 Vitality**	**Mean (SD)**	Active=17.5 (11.6)<br>Inactive=22.9 (24.6)		Active=20 (8.4)<br>Inactive=26.5 (25.6)		Active=29.4 (12.6)<br>Inactive=30.5 (30.1)
	**Cohen's d**	0.28 (-0.63, 1.2)		0.39 (-0.73, 1.51)		0.05 (-0.92, 1.02)
**SF-36 Social functioning**	**Mean (SD)**	Active=20 (17.9)<br>Inactive=28.4 (29.1)		Active=39.6 (31)<br>Inactive=42.5 (35)		Active=20.8 (25.8)<br>Inactive=40 (33.7)
	**Cohen's d**	0.35 (-0.57, 1.27)		0.09 (-1.02, 1.2)		0.64 (-0.35, 1.64)

		Visit 1		Visit 2		Follow-up
**Measure**	**Statistic**	**Pre-TMS**	**Post-TMS**	**Pre-TMS**	**Post-TMS**	
**SF-36 Emotional role**	**Mean (SD)**	Active=12.5 (24.8)<br>Inactive=46.7 (50.2)		Active=25 (41.8)<br>Inactive=33.3 (41.6)		Active=59.3 (40.1)<br>Inactive=30 (48.3)
	**Cohen's d**	0.9 (-0.16, 1.95)		0.2 (-0.91, 1.31)		-0.66 (-1.66, 0.33)
**SF-36 Mental health**	**Mean (SD)**	Active=54.4 (20.8)<br>Inactive=54.5 (30)		Active=56 (14.8)<br>Inactive=56.8 (29.7)		Active=59.6 (18.4)<br>Inactive=59.6 (25.6)
	**Cohen's d**	0.01 (-0.91, 0.92)		0.03 (-1.08, 1.14)		0 (-0.97, 0.97)
**Barthel**	**Mean (SD)**	Active=12.3 (3.8)<br>Inactive=14.5 (5.6)		Active=12.5 (4.4)<br>Inactive=14.4 (5.6)		Active=14.9 (4.2)<br>Inactive=15.8 (5.2)
	**Cohen's d**	0.44 (-0.48, 1.37)		0.36 (-0.75, 1.48)		0.19 (-0.75, 1.14)
**WSAS**	**Mean (SD)**	Active=32.3 (3.4)<br>Inactive=29.1 (9.1)		Active=29.7 (8.3)<br>Inactive=23.9 (10.6)		Active=29.9 (9.9)<br>Inactive=23.2 (11.8)
	**Cohen's d**	-0.48 (-1.4, 0.45)		-0.63 (-1.76, 0.5)		-0.62 (-1.61, 0.37)

Key: CORE=10=Clinical Outcomes in Routine Evaluation-10 item; GAD-7=Generalised Anxiety Disorder-7 item; IQR=interquartile range; KG=kilogram; PHQ=Patient Health Questionnaire; SD=standard deviation; SF-36=Short Form Health Survey-36 item; TMS=transcranial magnetic stimulation; WSAS=Work & Social Adjustment Scale
