# Supplementary material for: Trial Of Neurostimulation In Conversion Symptoms (TONICS): a feasibility randomised controlled trial of transcranial magnetic stimulation for functional limb weakness
Source: BMJ Open. 2020 Oct 6;10(10):e037198. doi: 10.1136/bmjopen-2020-037198 (PMC7539585; doi:10.1136/bmjopen-2020-037198)
Supplement: Author's manuscript [file bmjopen-2020-037198.draft_revisions.pdf]

BMJ Open

BMJ Open is committed to open peer review. As part of this commitment we make the peer review history of every article we publish publicly available.

When an article is published we post the peer reviewers' comments and the authors' responses online. We also post the versions of the paper that were used during peer review. These are the versions that the peer review comments apply to.

The versions of the paper that follow are the versions that were submitted during the peer review process. They are not the versions of record or the final published versions. They should not be cited or distributed as the published version of this manuscript.

BMJ Open is an open access journal and the full, final, typeset and author-corrected version of record of the manuscript is available on our site with no access controls, subscription charges or pay-per-view fees (http://bmjopen.bmj.com).

If you have any questions on BMJ Open's open peer review process please email info.bmjopen@bmj.com

BMJ Open

# Trial Of Neurostimulation In Conversion Symptoms (‘TONICS’): a feasibility randomised controlled trial of transcranial magnetic stimulation for functional limb weakness

| | |
|---|---|
| Journal: | *BMJ Open* |
| Manuscript ID | bmjopen-2020-037198 |
| Article Type: | Original research |
| Date Submitted by the Author: | 23-Jan-2020 |
| Complete List of Authors: | Pick, Susannah; Institute of Psychiatry Psychology and Neuroscience<br>Hodsoll, John ; Institute of Psychiatry Psychology and Neuroscience<br>Stanton, Biba; Institute of Psychiatry Psychology and Neuroscience; King's College Hospital NHS Foundation Trust<br>Eskander, Amy; King's College Hospital NHS Foundation Trust<br>Stavropoulos, Ioannis; King's College Hospital NHS Foundation Trust<br>Samra, Kiran; King's College Hospital NHS Foundation Trust<br>Bottini, Julia; King's College Hospital NHS Foundation Trust<br>Ahmad, Hena; King's College Hospital NHS Foundation Trust<br>David, Anthony; University College London, Institute of Mental Health<br>Purves, Alistair; King's College Hospital NHS Foundation Trust<br>Nicholson, Timothy; Institute of Psychiatry Psychology and Neuroscience |
| Keywords: | Adult neurology < NEUROLOGY, Adult psychiatry < PSYCHIATRY, Clinical trials < THERAPEUTICS |
| | |

SCHOLARONE™
Manuscripts

*I, the Submitting Author has the right to grant and does grant on behalf of all authors of the Work (as defined in the below author licence), an exclusive licence and/or a non-exclusive licence for contributions from authors who are: i) UK Crown employees; ii) where BMJ has agreed a CC-BY licence shall apply, and/or iii) in accordance with the terms applicable for US Federal Government officers or employees acting as part of their official duties; on a worldwide, perpetual, irrevocable, royalty-free basis to BMJ Publishing Group Ltd ("BMJ") its licensees and where the relevant Journal is co-owned by BMJ to the co-owners of the Journal, to publish the Work in this journal and any other BMJ products and to exploit all rights, as set out in our licence.*

*The Submitting Author accepts and understands that any supply made under these terms is made by BMJ to the Submitting Author unless you are acting as an employee on behalf of your employer or a postgraduate student of an affiliated institution which is paying any applicable article publishing charge ("APC") for Open Access articles. Where the Submitting Author wishes to make the Work available on an Open Access basis (and intends to pay the relevant APC), the terms of reuse of such Open Access shall be governed by a Creative Commons licence – details of these licences and which Creative Commons licence will apply to this Work are set out in our licence referred to above.*

*Other than as permitted in any relevant BMJ Author's Self Archiving Policies, I confirm this Work has not been accepted for publication elsewhere, is not being considered for publication elsewhere and does not duplicate material already published. I confirm all authors consent to publication of this Work and authorise the granting of this licence.*

For peer review only

# Trial Of Neurostimulation In Conversion Symptoms ('TONICS'): a feasibility randomised controlled trial of transcranial magnetic stimulation for functional limb weakness

**Susannah Pick[1], John Hodsoll[1], Biba Stanton[1,2], Amy Eskander[2], Ioannis Stavropoulos[2], Kiran Samra[2], Julia Bottini[2], Hena Ahmad[2], Anthony S. David[3], Alistair Purves[2], & Timothy R. Nicholson[1‡]**

[1]Institute of Psychiatry, Psychology & Neuroscience, King's College London, London, UK
[2] King's College Hospital NHS Foundation Trust, London, UK
[3]Institute of Mental Health, University College London, London, UK

[‡]corresponding author
Dr Timothy Nicholson. Email: timothy.nicholson@kcl.ac.uk; Tel: 0207 848 5136

Word count: 4775
Tables: 2
Figures: 2
Supplementary files: 2
Keywords: functional neurological disorder, conversion disorder, transcranial magnetic stimulation, functional motor, trial, treatment



For peer review only - http://bmjopen.bmj.com/site/about/guidelines.xhtml

## ABSTRACT

**Objectives:** Transcranial magnetic stimulation (TMS) has been used therapeutically for functional (conversion) motor symptoms but there is limited evidence for its efficacy and the optimal protocol. We examined the feasibility of a novel randomised controlled trial (RCT) protocol of TMS to treat functional limb weakness.

**Design:** A double-blind (patient, outcome assessor) two parallel-arm, placebo-controlled RCT.

**Setting:** Specialist neurology and neuropsychiatry services at a large National Health Service Foundation Trust in London, UK.

**Participants:** Patients with DSM-5 diagnosis of functional limb weakness. Exclusion criteria included comorbid neurological or major psychiatric disorder, contraindications to TMS, or previous TMS treatment.

**Interventions:** Patients were randomised to receive either active (single-pulse TMS to primary motor cortex (M1) above resting motor-threshold) or inactive treatment (single-pulse TMS to M1 below resting motor-threshold). Both groups received two TMS sessions, four weeks apart.

**Outcome measures:** We assessed recruitment, randomisation, and retention rates. The primary outcome was patient-rated symptom change (Clinical Global Impression–Improvement scale, CGI-I). Secondary outcomes included clinician-rated symptom change, psychosocial functioning, and disability. Outcomes were assessed at baseline, both TMS visits and at 3-month follow-up.

**Results:** Twenty-two patients were recruited and twenty-one (96%) were successfully randomised (active=10; inactive=11). Nineteen (91%) patients were included at follow-up (active=9; inactive=10). Completion rates for most outcomes were good (80-100%). Most patients were satisfied/very satisfied with the trial in both groups, although ratings were higher in the inactive arm (active=60%, inactive=92%). Adverse events were not more common for the active treatment. Treatment effect sizes for patient-rated CGI-I scores were small-moderate (Cliff's delta=0.1-0.3), reflecting a more positive outcome for the active treatment. Effect sizes for secondary outcomes were variable.

**Conclusions:** Our protocol is feasible. The findings suggest that supra-motor threshold TMS of M1 is safe, acceptable and potentially beneficial as a treatment for functional limb weakness. A larger RCT is warranted.



For peer review only - http://bmjopen.bmj.com/site/about/guidelines.xhtml

**Trial registration:** ISRCTN51225587

## ARTICLE SUMMARY

### Strengths and limitations of this study

- The study examined the feasibility of a novel, placebo-controlled TMS protocol for treating functional limb weakness.
- The TMS protocol has potential to inform the minimal dose required and mechanism of action for positive outcomes in this population.
- Both patients and outcome assessors were blind to treatment allocation, but it was not possible to blind the delivery of the treatment.
- As this was a feasibility study with a small sample size, randomisation might not have adequately balanced group differences across the treatment arms.

For peer review only

## BACKGROUND

Functional neurological disorder (FND) is defined by neurological symptoms that are incompatible with other medical/neurological diagnoses [1]. FND can resemble any neurological disorder, with seizures, motor (e.g., limb weakness, tremor, dystonia, myoclonus) and sensory (visual, auditory, somatosensory) symptoms predominating. Quality of life and prognosis are often poor [2-4]. Despite recent developments in detection and diagnosis of the disorder [5], there is still a marked paucity of evidence-based, accessible treatment options. There is emerging evidence for the efficacy of some treatment modalities (e.g., specialist physiotherapy for motor symptoms or cognitive behavioural therapy for seizures) [6-9], but availability is currently limited. The development of alternative treatment options that are safe, cost-effective, acceptable to patients and accessible is critical for improving outcomes in this population.

Transcranial magnetic stimulation (TMS) has been explored as a potential treatment option for functional motor symptoms and there is accumulating evidence for its efficacy and safety from uncontrolled studies and five randomised controlled trials (RCTs) [10-15]. These studies used divergent methods and so the optimal protocol is presently unclear, for example, whether to use single pulse (spTMS) or repetitive (rTMS) stimulation; which brain region to target; how many sessions are needed; and the optimal control intervention. Previous studies have generally found post-intervention functional motor symptom improvements following stimulation of primary motor cortex (M1) [11-15]. However, few of these RCTs reported gains in other important outcomes (e.g., comorbid psychological/physical symptoms, quality of life/global functioning, healthcare resource use). Despite post-treatment



For peer review only - http://bmjopen.bmj.com/site/about/guidelines.xhtml

improvements in core FND symptoms following rTMS to M1, Taib et al. [14] for example, did not observe superior improvements in health-related quality of life (SF-36 vitality/general health) for active rTMS relative to sham-TMS, and no improvements were observed in psychological symptoms. Similarly, McWhirter et al. [15] reported improvements in subjective symptoms immediately following spTMS of M1 relative to standard care, but no associated improvements in self-reported mental or physical health (SF-12) or clinician-rated disability (Modified Rankin Scale).

Further research is therefore needed to optimise both TMS treatment and RCT protocols to enable more definitive testing of the efficacy of TMS in improving functional motor symptoms themselves as well as other important outcome domains [16, 17].

## OBJECTIVES

We aimed to explore the feasibility and acceptability of a novel, placebo-controlled spTMS protocol for functional limb weakness, to inform the design and implementation of a subsequent larger-scale RCT. The protocol consisted of a minimal 'dose', consisting of two brief sessions of spTMS to M1, with the target region tailored to the specific limb weakness reported by each patient. We compared active stimulation delivered above resting motor threshold (RMT) to a placebo control condition, consisting of exactly the same procedures delivered below RMT. We hypothesised that this protocol would be feasible in terms of the following key parameters: recruitment rates, acceptance of randomisation, tolerance of the intervention, successful blinding and completion of outcome measures. We also



For peer review only - http://bmjopen.bmj.com/site/about/guidelines.xhtml

aimed to estimate the variability of outcome measures and treatment effect sizes to inform design of the next RCT.

## METHODS

### Trial design

The study was a double-blind two parallel arm placebo-controlled feasibility RCT of tailored spTMS to M1 in patients with functional limb weakness. The primary outcome was patient-rated symptom change. We also measured a range of other relevant secondary outcome domains to assess their feasibility and acceptability in this population (outlined below).

### Study setting and participants

Ethical approval was received from the London-Stanmore Research Ethics Committee, UK (ref:17/LO/0410). Patients with functional limb weakness were recruited from inpatient and outpatient neurology and neuropsychiatry services across the King's Health Partners (National Health Service, UK), including King's College Hospital, Guy's and St Thomas' Hospital, and the South London and Maudsley NHS Foundation Trusts. Recruitment took place between October 2017 and March 2018.

Inclusion criteria were:

- DSM-5 diagnosis of functional neurological disorder confirmed by a consultant neuropsychiatrist or neurologist



For peer review only - http://bmjopen.bmj.com/site/about/guidelines.xhtml

- Motor symptoms defined by functional weakness of at least one limb
- 18 years old or older
- Capacity to consent

Exclusion criteria were:

- Epilepsy (or considered high risk of epilepsy from medical history)
- Other contraindication to TMS (e.g. cochlear implants, metallic intracranial clips or intracranial surgery in last 12 months)
- Comorbid neurological condition (e.g. multiple sclerosis, stroke)
- Pain as primary symptom
- Previous treatment with TMS (for any condition)
- Non-fluent English speakers (if unable to accurately complete self-report questionnaires)
- Major mental health disorder: current diagnosis of schizophrenia or bipolar disorder; current drug/alcohol dependence
- History of factitious disorder
- Currently involved in another trial

Preliminary eligibility screening was completed by clinical neurology and neuropsychiatry staff. When patients were considered potentially eligible, Participant Information Sheets were provided, and permission sought for the research team (TN/SP) to contact the patient. When permission was granted, a member of the research team subsequently contacted the patient to answer any questions and arrange an initial screening assessment visit, if the patient wished to enrol. Written informed consent was obtained at the initial screening visit, after the study had been



For peer review only - http://bmjopen.bmj.com/site/about/guidelines.xhtml

explained in full and any remaining questions answered. Participants were not reimbursed for involvement in the study, but assistance with travel arrangements and expenses was provided, as necessary.

**Patient and Public Involvement**

A specialist service user advisory group was set up to inform the design and conduct of the study. Key national and international patient groups are involved in the dissemination plans.

**Background/screening measures**

At the initial screening visit, demographic details and medical history were obtained and a formal psychiatric screening tool administered (MINI International Neuropsychiatric Interview)[18]. Additional background measures were administered, including a personality disorder screen (Standardised Assessment of Personality – Self-Report, SAPAS-SR)[19], a measure of estimated intellectual functioning (National Adult Reading Test, NART)[20], and a trauma inventory (Childhood Experiences of Care and Abuse Questionnaire, CECA-Q)[21].

**Intervention**

Participants were randomised to receive active or inactive TMS, as described below. Both groups received two TMS sessions, separated by approximately 4 weeks.

***Active TMS***

The active treatment consisted of spTMS delivered to M1 including stimuli above resting motor threshold (RMT), thereby causing observable movement of the target



For peer review only - http://bmjopen.bmj.com/site/about/guidelines.xhtml

limb. The target limb was determined for each participant, defined as the weakest limb (i.e. arm or leg on either side) that caused most significant functional impairment in daily life. The target limb remained unchanged throughout both treatment sessions. The treatment was delivered in 2 phases:

*Phase 1: Measuring resting motor threshold (RMT)*

Single pulses were delivered with a Magstim 200 (Magstim, Whitland U.K.) TMS machine either using a circular coil to the area of M1 corresponding to the hand region of both the symptomatic and non-symptomatic arms, or using a double cone coil to deliver pulses to the M1 area for the legs (for participants with leg weakness only). As double cone coils cannot target left or right legs separately (M1 for both legs are stimulated) the same procedure was repeated twice as if targeting each side individually so that the procedure was the same for legs as it was for arms.

Pulses started at 20% of machine output and increased at increments of 5% until the evoked response (measured by surface electromyography in the first dorsal interosseous of the hand or extensor digitorum brevis of the foot) exceeded 50mcV in 50% of trials using standardised protocols [22]. This value was recorded as the RMT. As a variable number of pulses was needed to establish RMT in each patient, further pulses were then delivered at an interval of 5-10 seconds so that a total of 100 stimuli were delivered (50 stimuli to the same region of M1 bilaterally), to ensure that all participants received an equal number of stimuli during this phase.

*Phase 2: Supra-threshold (Active) TMS*



For peer review only - http://bmjopen.bmj.com/site/about/guidelines.xhtml

A further 20 pulses, again at an interval of 5-10 seconds, were delivered at 120% of RMT, applied to the region of M1 corresponding to the participant's weakest limb. No deliberate effort was made by the TMS deliverer to draw attention to the movement of the target limb. A total of 120 pulses were delivered during each of the two treatment sessions. The total number of 120 stimuli was adopted because 100 stimuli is the minimum required to reliably measure RMT and an extra 20 stimuli were needed for clear supra-threshold stimulation for therapeutic effect. This number has been recommended in standardised protocols for RMT measurement [22].

***Inactive (control) TMS***

The inactive treatment consisted of spTMS delivered to M1 that was always below RMT, thereby not leading to observable movement of the target limb. Phase 1 was identical to the procedures outlined above for measuring RMT.

*Phase 2: Sub-motor threshold (inactive) TMS*

A further 20 pulses at 80% of RMT were applied to the region of M1 corresponding to the patient's weakest limb. Whilst this constituted 'real' TMS, these stimuli did not produce any limb movement. Therefore, the key difference between the treatment conditions was whether stimulation was delivered above or below RMT and the initiation of automatic limb movement or not, respectively. As with the active treatment, a total of 120 stimuli were delivered during each TMS session.

**Changes to protocol during trial**

The original protocol specified that the second TMS session would follow the first within a narrowly defined period (30 +/- 2 days); however, during the course of the



For peer review only - http://bmjopen.bmj.com/site/about/guidelines.xhtml

For peer review only

trial it became clear that this was too restrictive and therefore not practicable, so the time period permitted between treatment sessions was extended (TMS session 2 to occur 28-50 days after TMS session 1).

**Outcome measures**

Outcome measures were completed before and/or after the first TMS session (baseline), before and/or after the second TMS session and three-months after the first TMS session. The primary outcome measure was patient-rated symptom change assessed with the Clinical Global Impression Improvement (CGI-I) scale [23], given the emerging consensus that patient-rated, subjective symptom improvements are particularly meaningful outcomes in this disorder [16, 17].

A range of secondary outcome measures was also included to assess the feasibility of measuring other relevant outcome domains in this group:

- outcome-rater and carer assessed symptom change (CGI-I scale)
- manual muscle testing (MRC strength scale performed by neurologist)
- dynamometry (if upper limb weakness present)
- subjective ratings of strength (0-100%) and weakness (1-5) in the weakest/target limb
- somatic symptoms (Patient Health Questionnaire (PHQ)-15) [24]
- depression (PHQ-9) [25]
- overall psychological distress (Core Outcomes in Routine Evaluation – 10, CORE-10) [26]
- quality of life (Short-Form Health Survey – 36, SF-36) [27]



For peer review only - http://bmjopen.bmj.com/site/about/guidelines.xhtml

- anxiety (Generalised Anxiety Disorder Questionnaire – 7 item, GAD-7) [28]
- disability / physical functioning (Barthel Index / Functional Independence Measure and Functional Assessment Measure (FIM/FAM) [29, 30]
- social and occupational functioning (Work and Social Adjustment Scale, WSAS) [31]
- healthcare utilisation (Client Service Receipt Inventory, CSRI) [32]

**Randomisation and blinding**

Randomisation occurred after the initial screening visit, once eligibility and consent had been confirmed. Randomisation was carried out online by the King's Clinical Trials Unit (KCTU) at the Institute of Psychiatry, Psychology and Neuroscience (IoPPN), using block randomisation. Computer-generated randomisation was initiated when the trial outcome-rater (SP) entered the initials and date-of-birth of the participant onto an online system. Randomisation was then conducted automatically and a confidential email with treatment allocation (active or inactive) sent directly to the TMS deliverer (TN). The outcome-rater (SP) remained blind to treatment allocation throughout the study, as did participants.

After completion of all study visits for each participant, blinding of the outcome-rater and participant were tested with a forced-choice question about which treatment the patient had received (active or inactive). The patient and outcome-rater answered the question independently. At the end of the study, participants were unblinded individually by the Principal Investigator (TN) during debriefing, with the outcome-rater absent from the room.  The outcome-rater remained blind to treatment allocation until all outcome data analyses were completed by the trial statistician.



For peer review only - http://bmjopen.bmj.com/site/about/guidelines.xhtml

### Safety monitoring

Adverse events (AEs) were monitored and recorded at each study visit and reported to the Principal Investigator (TN) or Trial Steering Committee as appropriate. Patients were invited to contact the research team at any time during the trial, in case of an AE occurring between visits.

### Statistical analysis

#### *Sample size determination*

Published data on TMS in FND indicates an improvement rate of approximately 10%, albeit on the basis of uncontrolled data. As spontaneous recovery rates are very low, a 10% improvement rate in the control arm at 1 month would be a conservative figure. From a previous CBT trial in FND, we would expect 30% of consented patients to decline randomisation and then 10% drop out. Hence with alpha=0.05 and 90% power, to detect an improvement rate of 80% in the active treatment arm relative to 10% in the control (z test between two independent proportions), 9 patients would be needed per arm.  For 18 patients to complete the study, given a 10% drop out, we would need to randomise 20 participants (30 consented). This allows 10% dropout rate to be assessed with an expected 95% CI of 0% to 24%.

#### *Feasibility parameters*

Data analysis was carried out by the blinded trial statistician (JH) and adopted the intention-to-treat (ITT) principle. The aims of the analysis were to examine trial feasibility parameters as follows:



For peer review only - http://bmjopen.bmj.com/site/about/guidelines.xhtml

- recruitment, randomisation and loss to follow-up rates
- tolerance of treatment, safety, treatment fidelity, participant / outcome-rater blinding and patient satisfaction
- estimate treatment effect sizes as potential outcomes of future trials

The analysis primarily consisted of descriptive statistics to summarise the rates of consent and randomisation of eligible patients, study retention, data quality (i.e., completion of outcome measures, missing data) and the acceptability of TMS to the patient population. Participant demographic and clinical characteristics were also described at baseline.

To assess improvement in symptoms, estimates of treatment effect sizes and 95% confidence intervals on the primary outcome measure (patient-rated CGI-I scale) were obtained using Cliff's Delta as this scale is ordinal. Cliff's Delta is a functional equivalent to Cohen's d for ordinal data, which does not make assumptions about the shape or spread of the distribution. The effect size values can be interpreted as reflecting the number of times a value in one distribution (active group) is higher than the value in the other distribution (inactive group). Criteria for interpreting the effect size were given by Romano et al. [33], with delta < 0.147 being negligible, delta < 0.33 small, delta < 0.474 being medium and otherwise large. For the secondary outcomes, descriptive statistics and effect sizes were calculated as appropriate for the type of data. Effect sizes (and 95% CI) for secondary outcomes were presented as Cohen's d or Cliff's delta as appropriate to the outcome data.



For peer review only - http://bmjopen.bmj.com/site/about/guidelines.xhtml

# RESULTS

## Sample characteristics

### *Demographics*

The demographic characteristics of participants at enrolment to the study are displayed in Table 1. The average age in each group was similar and the majority of participants in both groups was female, right-handed, married/cohabiting, and most often of white or black British ethnicity. Participants were most likely to report holding an undergraduate degree or vocational qualification. Participants were most often unemployed, but a proportion of patients reported being retired due to ill-health or employed full-time.

### *Background / clinical characteristics*

Table 2 shows key background and clinical features of participants at entry to the study. The MINI screen identified one patient with possible current psychosis, who was subsequently excluded and referred to appropriate clinical services. In eligible patients, the most common comorbid mental health diagnoses identified were major depressive disorder (n=8, 38%) and post-traumatic stress disorder (n=6, 29%). A larger proportion of the inactive group reported additional FND symptoms (i.e., other than limb weakness), relative to the active group. The duration of time since diagnosis was longer for the inactive group, although the duration since symptom onset was similar across groups. A similar proportion of patients in each group reported concurrent interventions at entry to the study and the average number of medications taken was approximately equal.



For peer review only - http://bmjopen.bmj.com/site/about/guidelines.xhtml

## Table 1 – Participant demographic characteristics

| | | Active TMS (N=10) | Inactive TMS (N=11) |
|---|---|---|---|
| **Age (Median, interquartile range)** | | 38 (32.5, 46.5) | 41 (33.5, 51) |
| **Gender** | **Female** | 8 (80) | 10 (90.9) |
| | **Male** | 2 (20) | 1 (9.1) |
| **Marital Status** | **Single** | 5 (50) | 3 (27.3) |
| | **Cohabiting / Married** | 5 (50) | 7 (63.6) |
| | **Separated / Divorced** | 0 (0) | 1 (9.1) |
| **Qualifications** | **None** | 0 (0) | 1 (9.1) |
| | **GCSE** | 4 (40) | 1 (9.1) |
| | **A Levels** | 1 (10) | 0 (0) |
| | **Graduate** | 3 (30) | 3 (27.3) |
| | **Postgraduate** | 0 (0) | 1 (9.1) |
| | **Vocational** | 2 (20) | 5 (45.5) |
| **Employment** | **Full Time** | 1 (10) | 3 (27.3) |
| | **Part Time** | 2 (20) | 0 (0) |
| | **Unemployed** | 7 (70) | 4 (36.4) |
| | **Retired (ill health)** | 0 (0) | 4 (36.4) |
| **Handedness** | **Right** | 8 (80) | 8 (72.7) |
| | **Left** | 2 (20) | 2 (18.2) |
| | **Ambidextrous** | 0 (0) | 1 (9.1) |
| **Ethnicity** | **White British** | 5 (50) | 7 (63.6) |
| | **Irish** | 1 (10) | 0 (0) |
| | **White and Black Caribbean** | 0 (0) | 1 (9.1) |
| | **Mixed** | 1 (10) | 0 (0) |
| | **Black British** | 2 (20) | 2 (18.2) |
| | **Caribbean** | 0 (0) | 1 (9.1) |
| | **Other** | 1 (10) | 0 (0) |

**Key:** TMS=transcranial magnetic stimulation

For peer review only

### Table 2 – Background/clinical characteristics by treatment group

| | Active TMS (n=10) | Inactive TMS (n=11) |
|---|---|---|
| **SAPAS-SR Total scores (median, IQR)** | 3 (2, 4.8) | 3 (2, 4) |
| **NART estimated IQ scores (median, IQR)** | 107 (105, 113) | 108 (108, 112) |
| **Psychiatric comorbidity present (baseline) (n, %)** | 6 (60) | 5 (45.5) |
| **Other FND symptoms (baseline) (n, %)** | 5 (50) | 9 (81.8) |
| **Age at FND onset, years (median, IQR)** | 35 (28.25, 45) | 31 (23.5, 48.5) |
| **Duration of FND, months (baseline) (median, IQR)** | 41 (14.75 ,63) | 42 (37, 107.5) |
| **Duration since FND diagnosis, months (baseline) (median, IQR)** | 1 (0, 5.25) | 12 (0.5, 38.5) |
| **Number of current medications (median, IQR)** | | |
| **Baseline** | 3 (2.25, 11) | 4 (3.5, 6) |
| **TMS session 1** | 3 (2, 11) | 4 (3.5, 6.5) |
| **TMS session 2** | 7 (2.25, 12.5) | 4.5 (3.25, 6.5) |
| **Follow-up** | 3 (2, 12) | 5 (3.5, 7) |
| **Concurrent treatments (n, %)** | | |
| **Baseline** | 10 (100) | 10 (91) |
| **TMS session 1** | 10 (100) | 10 (91) |
| **TMS session 2** | 6 (100) | 9 (90) |
| **Follow-up** | 9 (100) | 9 (82) |

**Key:** FND=functional neurological disorder; IQR=interquartile range; MDD=major depressive disorder; MINI=MINI International Neuropsychiatric Interview; NART=National Adult Reading Test; PTSD=post-traumatic stress disorder; SAPAS-SR=Standardised Assessment of Personality Abbreviated Scale–Self-Report; TMS=transcranial magnetic stimulation

## Feasibility parameters

Figure 1 displays rates of recruitment, treatment allocation, completion of the study and participants included in the data analysis (CONSORT flow diagram).

<insert Figure 1>

### *Recruitment, attendance and completion*

Of 32 potential candidates referred to the study, 22 consented to participate. Of these, 21 were found to be eligible at baseline screening. All 21 eligible patients were randomised and attended the first TMS treatment session. A total of five



For peer review only - http://bmjopen.bmj.com/site/about/guidelines.xhtml

For peer review only

patients did not attend the second TMS session (active=4; inactive=1), none gave reasons directly related to the intervention (Figure 1). At follow-up, two patients did not attend (active=1; inactive=1). Recruitment of the target number of participants (n=20) was completed within six months. The final follow up session took place approximately nine months after commencement of the study.

### *Data quality*

For each visit, the percentage return for each outcome measure was calculated in relation to the number of patients who attended that session (Supplementary File 1). Completion rates for the primary outcome measure (patient-rated CGI-I scale) was 100% at all timepoints. For most other measures, return rates were between 90-100% (i.e., outcome-rater CGI-I scale, Barthel Index, GAD-7, PHQ-9, PHQ-15, WSAS, CORE-10, most SF-36 subscales). A small number of scales were completed less consistently, although rates were still above 80% (e.g., SF-36 Role Emotional at TMS session 1, patient strength ratings/dynamometry at follow-up). Two measures were completed infrequently (carer-rated CGI-I scale/FIM-FAM) in 25% or fewer of the attendees at each timepoint.

### *Blinding*

There were no unexpected compromises to blinding during the study procedures. When asked with a forced-choice question at the end of the study, the active treatment was more likely to be correctly guessed as active by both patients (40%) and the outcome-rater (50%), compared to the inactive treatment (patients=36%; outcome-rater=27%). The percentage of correct responses by either informant was not above chance.



For peer review only - http://bmjopen.bmj.com/site/about/guidelines.xhtml

### *Patient satisfaction*

Patients' ratings of their overall experiences of the trial were good. The majority of patients (76%) stated that they were either 'somewhat' or 'very' satisfied with the trial, although ratings were higher in the inactive group (active=60%, inactive=92%). None of the patients in either group reported being 'unsatisfied' (neither 'somewhat' nor 'very'). Qualitatively, patients reported feeling pleased with the level of support and information provided by the research team, felt valued, found assistance with travel arrangements beneficial, and were pleased to be part of a study that could help people with FND more broadly. For some patients, lack of improvement and/or unwanted side effects were noted in the feedback to explain less positive satisfaction ratings (i.e., 'neither satisfied nor unsatisfied').

### *Adverse events*

There were four serious adverse events (SAEs) reported during the study (active=3; inactive=1). One SAE occurred between TMS session 1 and 2, and the other three occurred between TMS session 2 and follow-up. There were no SAEs immediately following a TMS session and none of the SAEs were considered related to the treatment by the Trial Steering Committee. In total there were 78 (non-serious) adverse events (AEs) with 15 of these occurring before the first treatment session. Following the start of treatment, there were 26 AEs in the active group and 37 in the inactive group. A proportion of patients in each group reported headaches at some time during the trial, but rates were slightly higher in the inactive group (n=5) relative to the active group (n=3). Worsening of FND symptoms was reported by some patients in each group at one or more time point, but the frequency of such reports was higher in the inactive group (15) compared to the active group (12).



For peer review only - http://bmjopen.bmj.com/site/about/guidelines.xhtml

**Primary outcome: patient-rated CGI-I scores**

Figure 2 displays the patient-rated CGI-I scores by group. Immediately prior to TMS session 1, 1 participant (9%) in the inactive group and 0% of the active group rated their symptoms as 'much improved' relative to their condition at entry to the study. Immediately after TMS session 1, these ratings remained the same. Immediately prior to TMS session 2, 67% of patients in the active group and 20% in the inactive group reported that their symptoms were 'much improved'. The relative percentage of 'much improved' again remained the same immediately following TMS session 2. Finally, at three-month follow-up, the number 'much improved' was 44% in the active group and 20% in the inactive group.

<insert Figure 2>

Effect sizes and 95% confidence intervals (Cliff's Delta) for patient-rated CGI-I scores were calculated. The effect size was positive prior to TMS session 1 reflecting coincidentally worse ratings in the active group (Cliff's delta=0.35 (-0.17, 0.71)). This difference remained the same immediately following TMS session 1 (Cliff's delta=0.35 (-0.15, 0.7)). However, this pattern was reversed by TMS session 2, indicating a benefit for the active treatment with moderate effect sizes pre- (Cliff's delta = -0.35 (-0.73, 0.19)) and post-treatment (Cliff's delta = -0.44 (-0.79, 0.13)). At three-month follow-up there was still an advantage for the active treatment; however, the difference was smaller (Cliff's delta = -0.2 (-0.6, 0.28)), potentially due to a relative improvement in the inactive group.



For peer review only - http://bmjopen.bmj.com/site/about/guidelines.xhtml

### Secondary outcomes

Descriptive statistics, effect sizes and confidence intervals for the secondary outcomes can be found in Supplementary File 2. There was considerable variability in the effect sizes and 95% confidence intervals for these outcomes and so the findings cannot be interpreted conclusively. However, the pattern of findings for the following outcomes suggested a benefit of active TMS: outcome-rater CGI-I scores, psychological distress (CORE-10), aspects of quality of life (SF-36 physical functioning, vitality/energy, role limitations due physical and emotional factors), activities of daily living (Barthel), primary care service use. The following outcomes did not suggest a benefit of active TMS: grip strength (dynamometry), subjective (patient-rated) limb strength, additional physical symptoms (PHQ-15), anxiety (GAD-7), depression (PHQ-9), some aspects of quality of life (SF-36 bodily pain, social functioning, mental health), social/occupational functioning (WSAS), inpatient hospital admissions and total outpatient healthcare contacts.

## DISCUSSION

This novel double-blind RCT of spTMS to M1 for the treatment of functional limb weakness was found to be feasible in terms of key parameters allowing estimation of the effect sizes for key outcome variables, and to inform the planning and implementation of a larger RCT.

### Feasibility

Rates of recruitment and retention were acceptable, with only two patients (10%) failing to complete the follow-up visit. Whilst 5 patients did not attend TMS session 2, none of these instances was directly related to the nature of the intervention.



For peer review only - http://bmjopen.bmj.com/site/about/guidelines.xhtml

Nevertheless, consideration should be given to ways of improving attendance rates at the second TMS session, such as offering the session earlier (e.g., after 1 or 2 weeks) and ensuring that any barriers to attendance are identified and managed in advance.

Completion of outcome measures was generally good with rates of 90-100% for most scales. However, the carer-rated CGI-I scale and the FIM-FAM were not completed frequently. Reasons for the lack of completion of the carer-rated CGI-I related to carers not being present or different carers attending each appointment. In future, a specific carer could be identified at the start of the study (in consultation with the patient) and ratings could be obtained by telephone, should that carer be absent at specific visits. It became clear that the FIM-FAM was not a suitable measure for this study, because it requires completion on an inpatient basis, usually by one or more members of a multidisciplinary clinical team. In this study, patients were recruited from a range of outpatient and inpatient settings, and ratings from inpatient clinical teams were at times difficult to obtain. Furthermore, several items on the measure replicated similar constructs assessed within other measures used in the trial (i.e., Barthel, SF-36).

For peer review only

Blinding appeared to be successful, with correct identification of active treatment below chance for both the patients and outcome-rater at the end of the study. Patient satisfaction ratings were also encouraging, suggesting that the trial procedures and the intervention were acceptable in this population. There were no SAEs directly related to the intervention and rates of potentially related AEs (i.e., headaches, FND



For peer review only - http://bmjopen.bmj.com/site/about/guidelines.xhtml

symptom worsening) were not reported at higher rates in the active group. Adverse events should be closely monitored in future studies.

## Outcomes

### *Primary outcome – patient-rated symptom improvement*

Point estimates for the patient-reported symptom improvement showed superiority for the active spTMS intervention relative to the inactive intervention, with small to moderate effect sizes. Improvements were most apparent at TMS session 2 but were still evident at follow-up. It is notable that the pattern of scores on the outcome-rater CGI-I scale concurred with the patient-rated CGI-I scores. These findings suggest that tailored spTMS, delivered above RMT to the area of M1 corresponding to a target limb (i.e., that limb which is functionally weakest) and thus causing movement of that limb, potentially could lead to greater improvements than the same intervention delivered below RMT (i.e., not inducing observable movement). These results concur with those of other studies [11-15] which have previously shown improvements in subjective or objective measures of functional motor symptom severity following spTMS or rTMS to M1.

The mechanism(s) by which TMS to M1 yields improvements in functional motor symptoms is unclear. It is possible that a neuromodulatory mechanism may operate in protocols using rTMS and/or that a general placebo effect could be responsible for improvements in cases where patients/outcome assessors are not blind to treatment allocation. However, similarly to Garcin et al. [12], our study suggests that elicitation of normal function of the weak limb with minimal doses of spTMS is sufficient to induce improvements, at least in the short-term. Induction of observable normal



For peer review only - http://bmjopen.bmj.com/site/about/guidelines.xhtml

function in the limb might result in modification of patients' beliefs and expectations regarding limb functioning and the possibility of recovery, and/or may represent a form of motor retraining effect. It is notable that the improvements did not occur immediately after the first treatment but were instead evident by the second treatment session (pre-TMS), suggesting that whilst one TMS session was sufficient to induce change, the mechanism by which change occurred required time to manifest as symptom reduction.

The findings in this study suggest that the patient-rated CGI-I scale is acceptable and sensitive to change as a measure of symptom improvement in FND intervention studies, in accordance with previous findings across treatment modalities and FND symptom types. This measure has recently been recommended as a primary outcome measure in FND treatment studies [17].

***Secondary outcomes***

High rates of completion of most of the secondary outcome measures indicated that they are appropriate tools for use in future, similar studies. Of the range of outcome domains included, the clearest trends for intervention-related improvements were in activities of daily living/disability (Barthel), overall psychological distress (CORE-10), aspects of health-related quality of life (i.e., physical functioning, physical role, vitality, emotional role) and primary care service use. Whilst extreme caution should be exercised in interpreting these findings due to the small sample size, small-moderate effect sizes and variable confidence intervals, these initial findings suggest that active spTMS might be associated with improvements in aspects of mental health, daily functioning (i.e., roles, daily activities, physical) and treatment seeking,



For peer review only - http://bmjopen.bmj.com/site/about/guidelines.xhtml

in addition to core FND symptom improvements. This extends the findings of previous studies, which have generally demonstrated improvements in functional motor symptoms only. However, it is not possible to say whether improvements in these additional outcome domains followed or preceded motor symptoms.

**Strengths and limitations**

A key strength of this study included the use of a minimal TMS protocol (two brief sessions of spTMS only), which was acceptable to patients and therefore resulted in good treatment adherence rates. This minimal TMS protocol also has potential to be used as a widely accessible treatment that could be used as adjunct to other therapies in a range of settings.

The inclusion of a placebo control condition in this study was also an advantage. Our inactive intervention was similar enough to the active treatment (i.e., 'real' TMS) to reduce the risk of patients inadvertently becoming unblinded to treatment allocation. Furthermore, blinding of both patients and outcome assessors ensured that post-treatment gains were not due entirely to general placebo effects. The inclusion of patients with additional functional neurological symptoms, non-major psychiatric comorbidities and those undergoing concomitant treatments yielded a sample that was representative of the broader FND patient population, improving the generalisability of the findings.

However, it is possible that the additional interventions that some patients were undergoing may have facilitated some of the improvements reported following treatment. Future RCTs with larger samples should balance the influence of

For peer review only

concomitant treatments and/or any incidental baseline between-group differences in symptoms, background features, or other relevant variables.

Another limitation to note is that some degree of improvement in FND symptoms was observed in both groups prior to commencing the first TMS session, relative to enrolment to the study. It is therefore unclear whether the improvements observed following TMS reflected the effect of the intervention (including its anticipation) or the natural course of the disorder. Future studies might valuably include an additional standard care or waiting-list control group, to examine these factors.

## Conclusion

The findings suggest that active (supra-motor threshold) spTMS to M1 is a safe, efficient, acceptable, and potentially effective treatment for functional limb weakness, leading to improvements in core symptoms and potentially other important outcome domains. A larger, pilot RCT is now warranted, to obtain a more robust estimate of effect sizes and variability in outcomes for this promising intervention.

For peer review only

## FIGURE LEGENDS

**Figure 1 – CONSORT diagram**

**Figure 2 – Patient-rated CGI-I categories by treatment group and timepoint**

## DECLARATIONS

### Ethics approval and consent to participate

The study was reviewed and approved by the London-Stanmore NHS Research Ethics Committee - study reference number 17/LO/0410). All participants provided informed, written consent prior to involvement in the study.

### Consent for publication

Not applicable.

### Competing interests

Not applicable.

### Data availability

All data relevant to the study are included in the article or uploaded as supplementary information

### Funding

TN, SP and the study were funded by a National Institute of Health Research (NIHR) Clinician Scientist Award to TN. This article represents independent research funded by the NIHR. The views expressed are those of the authors and not necessarily those of the NHS, NIHR or the Department of Health and Social Care.



For peer review only - http://bmjopen.bmj.com/site/about/guidelines.xhtml

## Authors' contributions

TN, AP and AD developed the study design. TN wrote the ethics proposal/study protocol, recruited some participants and conducted the TMS sessions. SP recruited and screened participants, conducted baseline and all subsequent outcome assessments, cleaned/entered data, and wrote the first/subsequent drafts of the manuscript. JH conducted the statistical analyses, prepared the CONSORT flow diagram and some sections of the results. BS, KS, JB, HA, IS, and AE conducted clinical strength tests during outcome assessments. All authors contributed to editing of the manuscript for important intellectual content and approved the final version prior to submission.

For peer review only



For peer review only - http://bmjopen.bmj.com/site/about/guidelines.xhtml

## REFERENCES

[1] American Psychiatric Association. Diagnostic and statistical manual of mental disorders (DSM-5). 5th edn. Washington: American Psychiatric Association, 2013.

[2] Gelauff J, Stone J, Edwards M, *et al*. The prognosis of functional (psychogenic) motor symptoms: a systematic review. *J Neurol Neurosurg Psychiatry* 2014;**85**(2):220-226.

[3] Anderson KE, Gruber-Baldini AL, Vaughan CG, *et al*. Impact of psychogenic movement disorders versus Parkinson's on disability, quality of life, and psychopathology. *Mov Disord* 2007;**22**(15):2204-2209.

[4] Szaflarski JP, Hughes C, Szaflarski M, *et al*. Quality of life in psychogenic nonepileptic seizures. *Epilepsia* 2003;**44**(2):236-242.

[5] Espay A.J., Aybek S., Carson A., *et al*. Current concepts in diagnosis and treatment of functional neurological disorders. *JAMA Neurol* 2018;**75**(9):1132-1141.

[6] LaFrance WC, Jr., Baird GL, Barry JJ, *et al*. Multicenter pilot treatment trial for psychogenic nonepileptic seizures: A randomized clinical trial. *JAMA Psychiatry* 2014;**71**(9):997-1005.

[7] Goldstein LH, Chalder T, Chigwedere C, *et al*. Cognitive-behavioral therapy for psychogenic nonepileptic seizures: a pilot RCT. *Neurology* 2010;**74**(24):1986-1994.

[8] Nielsen G, Buszewicz M, Stevenson F, *et al*. Randomised feasibility study of physiotherapy for patients with functional motor symptoms. *J Neurol Neurosurg Psychiatry* 2017;**88**(6):484-490.

[9] Jordbru AA, Smedstad LM, Klungsøyr O, *et al*. Psychogenic gait disorder: a randomized controlled trial of physical rehabilitation with one-year follow-up. *J Rehabil Med* 2014;**46**(2):181-187.

[10] Pollak TA, Nicholson TR, Edwards MJ, *et al*. A systematic review of transcranial magnetic stimulation in the treatment of functional (conversion) neurological symptoms. *J Neurol Neurosurg Psychiatry* 2014;**85**(2):191-197.

[11] Broersma M, Koops EA, Vroomen PC, *et al*. Can repetitive transcranial magnetic stimulation increase muscle strength in functional neurological paresis? A proof-of-principle study. *Eur J Neurol* 2015;**22**(5):866-873.

[12] Garcin B, Mesrati F, Hubsch C, *et al*. Impact of transcranial magnetic stimulation on functional movement disorders: cortical modulation or a behavioral effect? *Front Neurol* 2017;**8**:338.



For peer review only - http://bmjopen.bmj.com/site/about/guidelines.xhtml

[13] Garcin B, Roze E, Mesrati F, *et al*. Transcranial magnetic stimulation as an efficient treatment for psychogenic movement disorders. *J Neurol Neurosurg Psychiatry* 2013;**84**(9):1043-1046.

[14] Taib S, Ory-Magne F, Brefel-Courbon C, *et al*. Repetitive transcranial magnetic stimulation for functional tremor: A randomized, double-blind, controlled study. *Mov Disord* 2019;**34**(8):1210-1219.

[15] McWhirter L, Ludwig L, Carson A, *et al*. Transcranial magnetic stimulation as a treatment for functional (psychogenic) upper limb weakness. *J Psychosom Res* 2016;**89**:102-106.

[16] Nicholson TR, Carson AJ, Edwards MJ, *et al*. Outcome measures for functional neurological disorder – a review of the theoretical complexities. *J Neuropsychiatry Clin Neurosci* 2020;**32**(1):33-42.

[17] Pick S, Anderson D, Asadi-Pooya A, *et al*. Outcome measurement in functional neurological disorder: a systematic review and recommendations. *J Neurol Neurosurg Psychiatry* (in press). doi:10.1136/jnnp-2019-322180.

[18] Sheehan DV, Lecrubier Y, Sheehan KH, *et al*. The Mini-International Neuropsychiatric Interview (M.I.N.I.): the development and validation of a structured diagnostic psychiatric interview for DSM-IV and ICD-10. *J Clin Psychiatry* 1998;**59 Suppl 20**:22-33.

[19] Germans S, Van Heck GL, Moran P, *et al*. The Self-report Standardized Assessment of Personality-abbreviated Scale: Preliminary results of a brief screening test for personality disorders. *Personal Ment Health* 2008;**2**(2):70-76.

[20] Nelson HE. National Adult Reading Test (NART): For the assessment of premorbid intelligence in patients with dementia: Test manual. Nfer-Nelson, 1982.

[21] Bifulco A, Bernazzani O, Moran P, *et al*. The childhood experience of care and abuse questionnaire (CECA. Q): validation in a community series. *Br J Clin Psychol* 2005;**44**(4):563-581.

[22] Mills KR. Magnetic Stimulation of the Human Nervous System. Oxford: Oxford University Press 1999.

[23] Guy W. ECDEU Assessment Manual for Psychopharmacology. Rockville, MD, US: Department of Health, Education, and Welfare, 1976.

[24] Kroenke K, Spitzer RL, Williams JB. The PHQ-15: validity of a new measure for evaluating the severity of somatic symptoms. *Psychosom Med* 2002;**64**(2):258-266.

[25] Kroenke K, Spitzer RL, Williams JBW. The PHQ-9. *J Gen Intern Med* 2001;**16**(9):606-613.



For peer review only - http://bmjopen.bmj.com/site/about/guidelines.xhtml

[26] Barkham M, Bewick B, Mullin T*, et al*. The CORE-10: A short measure of psychological distress for routine use in the psychological therapies. *Couns Psychother Res* 2013;**13**(1):3-13.

[27] Ware Jr JE, Sherbourne CD. The MOS 36-item short-form health survey (SF-36): I. Conceptual framework and item selection. *Med Care* 1992;**30**:473-483.

[28] Spitzer RL, Kroenke K, Williams JB*, et al*. A brief measure for assessing generalized anxiety disorder: the GAD-7. *Arch Intern Med* 2006;**166**(10):1092-1097.

[29] Mahoney FI, Barthel DW. Functional evaluation: the Barthel Index: a simple index of independence useful in scoring improvement in the rehabilitation of the chronically ill. *Md State Med J* 1965;**14**:61-65.

[30] Turner-Stokes L, Nyein K, Turner-Stokes T*, et al*. The UK FIM FAM: development and evaluation. *Clin Rehabil* 1999;**13**(4):277-287.

[31] Mundt JC, Marks IM, Shear MK*, et al*. The Work and Social Adjustment Scale: a simple measure of impairment in functioning. *Br J Psychiatry* 2002;**180**(5):461-464.

[32] Beecham J, Knapp M. Costing psychiatric interventions. Measuring mental health needs.Gaskell, London 2001;200-224.

[33] Romano J, Kromrey JD, Coraggio J*, et al*. Exploring methods for evaluating group differences on the NSSE and other surveys: Are the t-test and Cohen'sd indices the most appropriate choices. In *Annual Meeting of the Southern Association for Institutional Research* 2006;1-51.

For peer review only



For peer review only - http://bmjopen.bmj.com/site/about/guidelines.xhtml

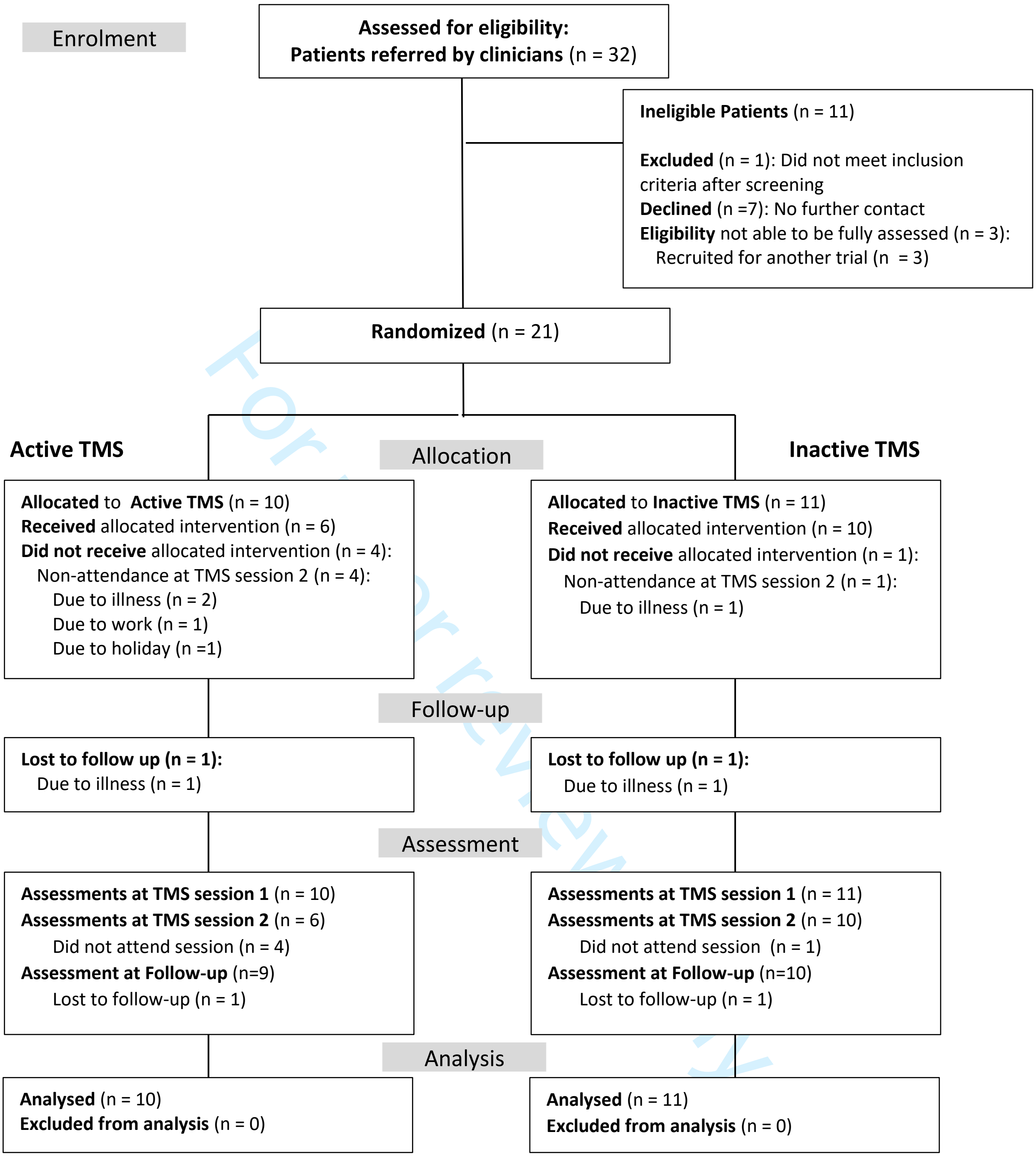


For peer review only - http://bmjopen.bmj.com/site/about/guidelines.xhtml

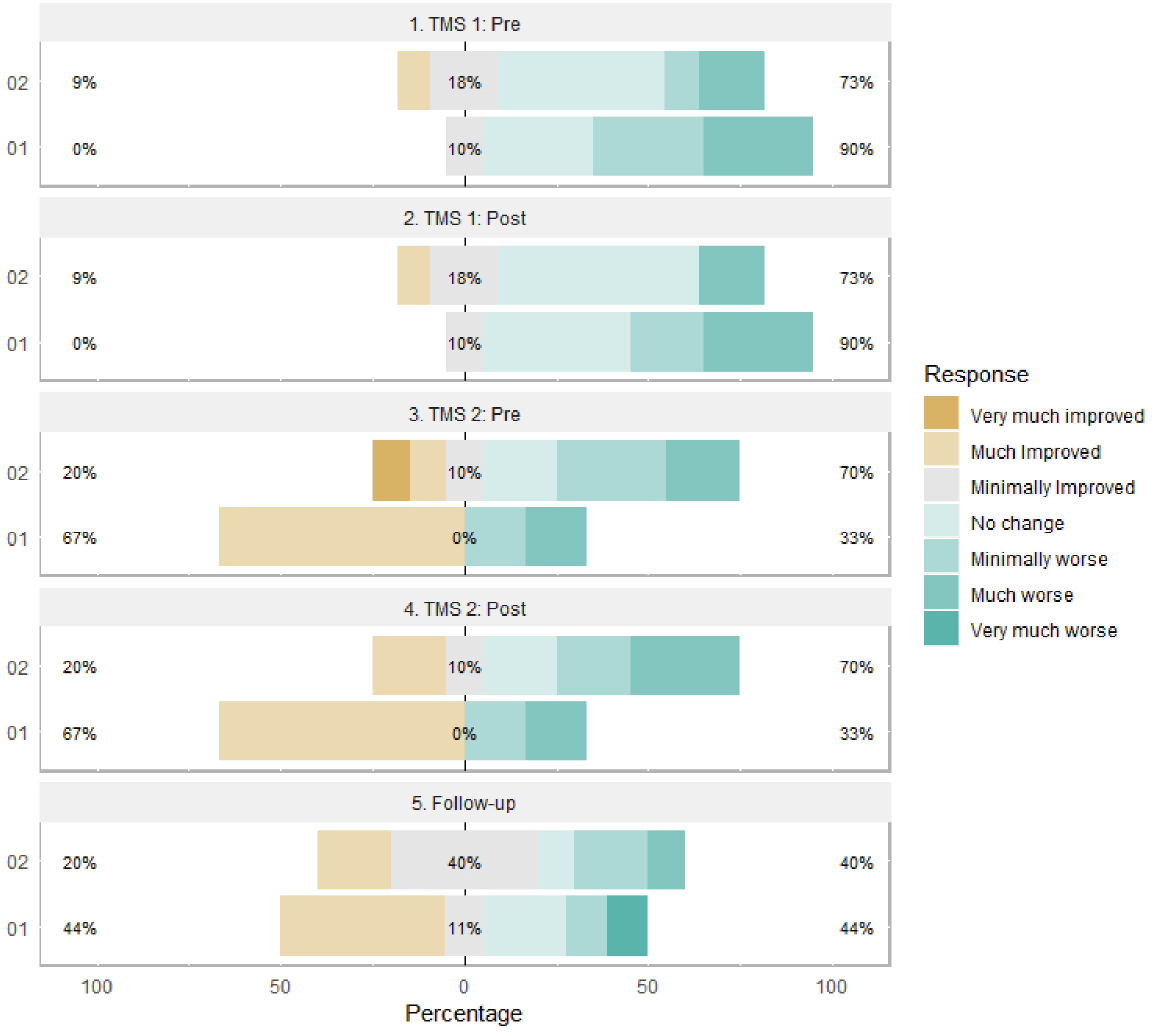


Key: 01=Active TMS; 02=Inactive TMS

For peer review only - http://bmjopen.bmj.com/site/about/guidelines.xhtml

## Supplementary File 1 – Outcome measure completion data

***Table 1.1. Data quality by group and timepoint****

| Outcome measure | TMS Visit 1 n (%) | TMS Visit 2 n (%) | Follow up n (%) |
|---|---|---|---|
| **CGI Patient** | 21 (100) | 16 (100) | 19 (100) |
| **CGI Outcome assessor** | 21 (100) | 16 (100) | 20 (105) |
| **CGI Carer** | 2 (10) | 4 (25) | 4 (21) |
| **SF36: Physical Function** | 21 (100) | 16 (100) | 19 (100) |
| **SF36: Role Physical** | 20 (95) | 16 (100) | 19 (100) |
| **SF36: Bodily Pain** | 21 (100) | 16 (100) | 19 (100) |
| **SF36: General Health** | 21 (100) | 16 (100) | 19 (100) |
| **SF36: Vitality** | 21 (100) | 16 (100) | 19 (100) |
| **SF36: Social Functioning** | 21 (100) | 16 (100) | 19 (100) |
| **SF36: Role Emotional** | 18 (86) | 16 (100) | 19 (100) |
| **SF36: Mental Health** | 21 (100) | 16 (100) | 19 (100) |
| **Barthel Index** | 21 (100) | 16 (100) | 20 (105) |
| **FIM-FAM** | 4 (19) | 2 (12) | 2 (11) |
| **GAD 7** | 21 (100) | 16 (100) | 19 (100) |
| **PHQ 9** | 21 (100) | 16 (100) | 19 (100) |
| **PHQ 15** | 21 (100) | 16 (100) | 19 (100) |
| **CORE-10** | 21 (100) | 16 (100) | 19 (100) |
| **WSAS** | 21 (100) | 16 (100) | 19 (100) |
| **Left Arm; Strength** | 20 (95) | 15 (94) | 17 (89) |
| **Left Arm: Weakness** | 20 (95) | 15 (94) | 18 (95) |
| **Right Arm: Strength** | 20 (95) | 15 (94) | 17 (89) |
| **Right Arm: Weakness** | 20 (95) | 15 (94) | 18 (95) |
| **Left Leg; Strength** | 21 (100) | 16 (100) | 18 (95) |
| **Left Leg: Weakness** | 21 (100) | 16 (100) | 19 (100) |
| **Right Leg: Strength** | 20 (95) | 15 (94) | 17 (89) |
| **Right Leg: Weakness** | 20 (95) | 15 (94) | 18 (95) |
| **Dynamometry Left Arm: Max** | 20 (95) | 15 (94) | 17 (89) |
| **Dynamometry Left Arm: Max** | 20 (95) | 15 (94) | 18 (95) |
| **Dynamometry Left Arm: Max** | 20 (95) | 15 (94) | 17 (89) |
| **Dynamometry Left Arm: Max** | 20 (95) | 15 (94) | 18 (95) |

**Key:** CGI=Clinical Global Impression; CORE=10=Clinical Outcomes in Routine Evaluation-10 item; GAD-7=Generalised Anxiety Disorder-7 item; KG=kilogram; PHQ=Patient Health Questionnaire; SF-36=Short Form Health Survey-36 item; TMS=transcranial magnetic stimulation; WSAS=Work & Social Adjustment Scale

***Percentages calculated relative to the number of patients in attendance in each group*

For peer review only

## Supplementary File 2 - Descriptive statistics and effect sizes for primary and secondary outcomes

### *Supplementary Table 2.1. Patient CGI-I ratings*

| | | Visit 1 | | | | Visit 2 | | | | Follow-up | |
|---|---|---|---|---|---|---|---|---|---|---|---|
| | | Pre-TMS | | Post-TMS | | Pre-TMS | | Post-TMS | | | |
| | | Active | Inactive | Active | Inactive | Active | Inactive | Active | Inactive | Active | Inactive |
| **Very much improved** | **n (%)** | 0 (0) | 0 (0) | 0 (0) | 0 (0) | 0 (0) | 1 (10) | 0 (0) | 0 (0) | 0 (0) | 0 (0) |
| **Much improved** | **n (%)** | 0 (0) | 1 (9) | 0 (0) | 1 (9) | 4 (67) | 1 (10) | 4 (67) | 2 (20) | 4 (44) | 2 (20) |
| **Minimally improved** | **n (%)** | 1 (10) | 2 (18) | 1 (10) | 2 (18) | 0 (0) | 1 (10) | 0 (0) | 1 (10) | 1 (11) | 4 (40) |
| **No change** | **n (%)** | 3 (30) | 5 (45) | 4 (40) | 6 (55) | 0 (0) | 2 (20) | 0 (0) | 2 (20) | 2 (22) | 1 (10) |
| **Minimally worse** | **n (%)** | 3 (30) | 1 (9) | 2 (20) | 0 (0) | 1 (17) | 3 (30) | 1 (17) | 2 (20) | 1 (11) | 2 (20) |
| **Much worse** | **n (%)** | 3 (30) | 2 (18) | 3 (30) | 2 (18) | 1 (17) | 2 (20) | 1 (17) | 3 (30) | 0 (0) | 1 (10) |
| **Very much worse** | **n (%)** | 0 (0) | 0 (0) | 0 (0) | 0 (0) | 0 (0) | 0 (0) | 0 (0) | 0 (0) | 1 (11) | 0 (0) |
| **Total** | **n (%)** | 10 (100) | 11 (100) | 10(100) | 11 (100) | 6 (60) | 10 (91) | 6 (60) | 10 (91) | 9 (90) | 10 (91) |
| **Missing*** | **n (%)** | 0 (0) | 0 (0) | 0 (0) | 0 (0) | 4 (40) | 1 (9) | 4 (40) | 1 (9) | 1 (10) | 1 (9) |
| **Effect size (negative = benefit)** | **Cliff's delta (95% CI)** | 0.35 (-0.17, 0.71) | | 0.35 (-0.15, 0.7) | | -0.35 (-0.73, 0.19) | | -0.44 (-0.79, 0.13) | | -0.2 (-0.6, 0.28) | |

**Key:** CGI-I=Clinical Global Impression-Improvement; CI=confidence interval; TMS=transcranial magnetic stimulation

**Percentage calculated relative to total number of participants enrolled in study*

For peer review only

***Supplementary Table 2.2. Outcome assessor CGI-I ratings***

| | | Visit 1 | | | | Visit 2 | | | | Follow-up | |
|---|---|---|---|---|---|---|---|---|---|---|---|
| | | Pre-TMS | | Post-TMS | | Pre-TMS | | Post-TMS | | | |
| | | Active | Inactive | Active | Inactive | Active | Inactive | Active | Inactive | Active | Inactive |
| **Very much improved** | **n (%)** | 0 (0) | 0 (0) | 0 (0) | 0 (0) | 0 (0) | 0 (0) | 0 (0) | 0 (0) | 0 (0) | 0 (0) |
| **Much improved** | **n (%)** | 0 (0) | 0 (0) | 0 (0) | 0 (0) | 3 (50) | 2 (20) | 3 (50) | 2 (20) | 4 (44) | 2 (18) |
| **Minimally improved** | **n (%)** | 1 (10) | 3 (27) | 1 (10) | 3 (27) | 1 (17) | 1 (10) | 1 (17) | 1 (10) | 3 (33) | 5 (45) |
| **No change** | **n (%)** | 3 (30) | 7 (64) | 4 (40) | 6 (55) | 0 (0) | 5 (50) | 0 (0) | 5 (50) | 1 (11) | 2 (18) |
| **Minimally worse** | **n (%)** | 3 (30) | 1 (9) | 1 (10) | 2 (18) | 2 (33) | 2 (20) | 2 (33) | 2 (20) | 0 (0) | 1 (9) |
| **Much worse** | **n (%)** | 3 (30) | 0 (0) | 4 (40) | 0 (0) | 0 (0) | 0 (0) | 0 (0) | 0 (0) | 0 (0) | 1 (9) |
| **Very much worse** | **n (%)** | 0 (0) | 0 (0) | 0 (0) | 0 (0) | 0 (0) | 0 (0) | 0 (0) | 0 (0) | 1 (11) | 0 (0) |
| **Total** | **n (%)** | 10 (100) | 11 (100) | 10 (100) | 11 (100) | 6 (60) | 10 (91) | 6 (60) | 10 (91) | 9 (90) | 11 (100) |
| **Missing*** | **n (%)** | 0 (0) | 0 (0) | 0 (0) | 0 (0) | 4 (40) | 1 (9) | 4 (40) | 1 (9) | 1 (10) | 0 (0) |
| **Effect size (negative = benefit)** | **Cliff's delta (95% CI)** | 0.55 (0.05, 0.83) | | 0.45 (-0.06, 0.77) | | -0.29 (-0.69, 0.25) | | -0.29 (-0.69, 0.25) | | -0.26 (-0.65, 0.23) | |

**Key:** CGI-I=Clinical Global Impression-Improvement; CI=confidence interval; TMS=transcranial magnetic stimulation

*****Percentage calculated relative to total number of participants enrolled in the study*

For peer review only

***Supplementary Table 2.3. Patient weakness ratings***

| | | Visit 1 | | | | Visit 2 | | | | Follow-up | |
|---|---|---|---|---|---|---|---|---|---|---|---|
| | | Pre-TMS | | Post-TMS | | Pre-TMS | | Post-TMS | | | |
| | | **Active** | **Inactive** | **Active** | **Inactive** | **Active** | **Inactive** | **Active** | **Inactive** | **Active** | **Inactive** |
| **No weakness** | **n(%)** | 2 (20) | 1 (9) | 3 (30) | 1 (9) | 0 (0) | 1 (10) | 1 (17) | 1 (10) | 2 (22) | 0 (0) |
| **Mild weakness** | **n(%)** | 1 (10) | 3 (27) | 0 (0) | 5 (45) | 1 (17) | 4 (40) | 0 (0) | 5 (50) | 1 (11) | 5 (50) |
| **Moderate weakness** | **n(%)** | 1 (10) | 3 (27) | 1 (10) | 0 (0) | 0 (0) | 1 (10) | 1 (17) | 0 (0) | 3 (33) | 1 (10) |
| **Severe weakness** | **n(%)** | 3 (30) | 1 (9) | 3 (30) | 2 (18) | 3 (50) | 2 (20) | 3 (50) | 3 (30) | 1 (11) | 3 (30) |
| **Very severe weakness** | **n(%)** | 3 (30) | 3 (27) | 3 (30) | 3 (27) | 2 (33) | 2 (20) | 1 (17) | 1 (10) | 2 (22) | 1 (10) |
| **Total** | **n(%)** | 10 (100) | 11 (100) | 10 (100) | 11 (100) | 6 (60) | 10 (91) | 6 (60) | 10 (91) | 9 (90) | 10 (91) |
| **Missing*** | **n(%)** | 0 (0) | 0 (0) | 0 (0) | 0 (0) | 4 (40) | 1 (9) | 4 (40) | 1 (9) | 1 (10) | 1 (9) |
| **Effect size (negative = treatment benefit)** | **Cliff's Delta (95% CI)** | 0.09 (-0.41, 0.55) | | 0.04 (-0.46, 0.51) | | 0.27 (-0.11, 0.58) | | 0.17 (-0.25, 0.53) | | -0.08 (-0.51, 0.37) | |

**Key:** CI=confidence interval; TMS=transcranial magnetic stimulation

*****Percentage calculated relative to total number of participants enrolled in the study*

For peer review only

***Supplementary Table 2.4. Additional secondary outcome measures***

| | | Visit 1 | | Visit 2 | | Follow-up |
|---|---|---|---|---|---|---|
| **Measure** | **Statistic** | **Pre-TMS** | **Post-TMS** | **Pre-TMS** | **Post-TMS** | |
| **Target limb strength rating (0-100%)** | **Mean (SD)** | Active=42.5 (37.4)<br>Inactive=52.3 (30.4) | Active=44.5 (40.6)<br>Inactive=52.7 (35) | Active=38.3 (25)<br>Inactive=55 (34) | Active=42.8 (34.1)<br>Inactive=57 (34) | Active=41.9 (27.5)<br>Inactive=51.8 (36.2) |
| | **Cohen's d** | 0.29 (-0.63, 1.21) | 0.22 (-0.7, 1.14) | 0.54 (-0.59, 1.66) | 0.42 (-0.7, 1.53) | 0.3 (-0.71, 1.31) |
| **Dynamometry – left arm (average KG)** | **Mean (SD)** | Active=12.4 (10.8)<br>Inactive=6.1 (6.9) | Active=11.3 (11.7)<br>Inactive=7 (8.9) | Active=11.9 (3.7)<br>Inactive=6.3 (11) | Active=11.6 (6.1)<br>Inactive=6.4 (12) | Active=10.7 (9.1)<br>Inactive=9.7 (12.3) |
| | **Cohen's d** | 0.68 (-0.35, 1.71) | 0.41 (-0.61, 1.42) | 0.65 (-0.6, 1.91) | 0.53 (-0.72, 1.77) | 0.09 (-1.02, 1.21) |
| **Dynamometry – right arm (average KG)** | **Mean (SD)** | Active=9.4 (9)<br>Inactive=10.5 (9.1) | Active=9.4 (8.6)<br>Inactive=9.6 (8.8) | Active=11.9 (6.6)<br>Inactive=10.3 (9.1) | Active=11.9 (9)<br>Inactive=9.6 (12.2) | Active=12.5 (12.9)<br>Inactive=11.1 (9.1) |
| | **Cohen's d** | -0.12 (-1.09, 0.85) | -0.02 (-0.99, 0.95) | 0.19 (-0.99, 1.37) | 0.21 (-0.97, 1.39) | 0.13 (-0.95, 1.2) |
| **PHQ-15** | **Mean (SD)** | Active=15.4 (3.3)<br>Inactive=13.5 (6) | | Active=15.7 (4.4)<br>Inactive=14.2 (7.2) | | Active=15.2 (5.3)<br>Inactive=12.4 (6) |
| | **Cohen's d** | -0.39 (-1.31, 0.54) | | -0.26 (-1.38, 0.85) | | -0.5 (-1.48, 0.49) |
| **PHQ-9** | **Mean (SD)** | Active=15 (5.2)<br>Inactive=14.1 (8.9) | | Active=13.3 (2.2)<br>Inactive=12.8 (8.4) | | Active=14.3 (6.1)<br>Inactive=12.3 (11.2) |
| | **Cohen's d** | -0.13 (-1.04, 0.79) | | -0.1 (-1.21, 1.01) | | -0.22 (-1.19, 0.75) |
| **GAD-7** | **Mean (SD)** | Active=8.7 (5.6)<br>Inactive=10.5 (7.7) | | Active=7.3 (3.4)<br>Inactive=7.5 (7) | | Active=7.1 (4.9)<br>Inactive=9.1 (7.6) |
| | **Cohen's d** | 0.28 (-0.64, 1.2) | | 0.03 (-1.07, 1.14) | | 0.32 (-0.66, 1.29) |

For peer review only

| | | Visit 1 | | Visit 2 | | Follow-up |
|---|---|---|---|---|---|---|
| **Measure** | **Statistic** | **Pre-TMS** | **Post-TMS** | **Pre-TMS** | **Post-TMS** | |
| **CORE-10** | **Mean (SD)** | Active=18.4 (8.3)<br>Inactive=17.1 (10.3) | | Active=16.7 (4)<br>Inactive=16.5 (9.4) | | Active=14.8 (5.2)<br>Inactive=16.4 (8.2) |
| | **Cohen's d** | -0.14 (-1.06, 0.77) | | -0.03 (-1.13, 1.08) | | 0.24 (-0.73, 1.21) |
| **SF-36 Physical functioning** | **Mean (SD)** | Active=10 (11.5)<br>Inactive=22.7 (22.2) | | Active=15.8 (21.3)<br>Inactive=30 (28.9) | | Active=21.2 (26.4)<br>Inactive=28 (29.6) |
| | **Cohen's d** | 0.73 (-0.21, 1.68) | | 0.58 (-0.55, 1.71) | | 0.24 (-0.73, 1.22) |
| **SF-36 Physical role** | **Mean (SD)** | Active=2.5 (7.9)<br>Inactive=15 (33.7) | | Active=4.2 (10.2)<br>Inactive=20 (36.9) | | Active=8.3 (25)<br>Inactive=17.5 (37.4) |
| | **Cohen's d** | 0.51 (-0.44, 1.46) | | 0.67 (-0.46, 1.81) | | 0.29 (-0.68, 1.27) |
| **SF-36 Bodily pain** | **Mean (SD)** | Active=22.2 (18.3)<br>Inactive=25 (27.1) | | Active=29.8 (27.7)<br>Inactive=19.1 (22.1) | | Active=31 (23.6)<br>Inactive=32.6 (21) |
| | **Cohen's d** | 0.12 (-0.79, 1.04) | | -0.42 (-1.53, 0.7) | | 0.07 (-0.9, 1.04) |
| **SF-36 General health** | **Mean (SD)** | Active=29.9 (9.7)<br>Inactive=30.8 (21.2) | | Active=38.2 (15.8)<br>Inactive=35.4 (26.2) | | Active=31.6 (11)<br>Inactive=39.8 (20.2) |
| | **Cohen's d** | 0.06 (-0.86, 0.97) | | -0.14 (-1.25, 0.97) | | 0.51 (-0.47, 1.5) |
| **SF-36 Vitality** | **Mean (SD)** | Active=17.5 (11.6)<br>Inactive=22.9 (24.6) | | Active=20 (8.4)<br>Inactive=26.5 (25.6) | | Active=29.4 (12.6)<br>Inactive=30.5 (30.1) |
| | **Cohen's d** | 0.28 (-0.63, 1.2) | | 0.39 (-0.73, 1.51) | | 0.05 (-0.92, 1.02) |
| **SF-36 Social functioning** | **Mean (SD)** | Active=20 (17.9)<br>Inactive=28.4 (29.1) | | Active=39.6 (31)<br>Inactive=42.5 (35) | | Active=20.8 (25.8)<br>Inactive=40 (33.7) |
| | **Cohen's d** | 0.35 (-0.57, 1.27) | | 0.09 (-1.02, 1.2) | | 0.64 (-0.35, 1.64) |

For peer review only

| | | Visit 1 | | Visit 2 | | Follow-up |
|---|---|---|---|---|---|---|
| **Measure** | **Statistic** | **Pre-TMS** | **Post-TMS** | **Pre-TMS** | **Post-TMS** | |
| **SF-36 Emotional role** | **Mean (SD)** | Active=12.5 (24.8)<br>Inactive=46.7 (50.2) | | Active=25 (41.8)<br>Inactive=33.3 (41.6) | | Active=59.3 (40.1)<br>Inactive=30 (48.3) |
| | **Cohen's d** | 0.9 (-0.16, 1.95) | | 0.2 (-0.91, 1.31) | | -0.66 (-1.66, 0.33) |
| **SF-36 Mental health** | **Mean (SD)** | Active=54.4 (20.8)<br>Inactive=54.5 (30) | | Active=56 (14.8)<br>Inactive=56.8 (29.7) | | Active=59.6 (18.4)<br>Inactive=59.6 (25.6) |
| | **Cohen's d** | 0.01 (-0.91, 0.92) | | 0.03 (-1.08, 1.14) | | 0 (-0.97, 0.97) |
| **Barthel** | **Mean (SD)** | Active=12.3 (3.8)<br>Inactive=14.5 (5.6) | | Active=12.5 (4.4)<br>Inactive=14.4 (5.6) | | Active=14.9 (4.2)<br>Inactive=15.8 (5.2) |
| | **Cohen's d** | 0.44 (-0.48, 1.37) | | 0.36 (-0.75, 1.48) | | 0.19 (-0.75, 1.14) |
| **WSAS** | **Mean (SD)** | Active=32.3 (3.4)<br>Inactive=29.1 (9.1) | | Active=29.7 (8.3)<br>Inactive=23.9 (10.6) | | Active=29.9 (9.9)<br>Inactive=23.2 (11.8) |
| | **Cohen's d** | -0.48 (-1.4, 0.45) | | -0.63 (-1.76, 0.5) | | -0.62 (-1.61, 0.37) |

**Key:** CORE=10=Clinical Outcomes in Routine Evaluation-10 item; GAD-7=Generalised Anxiety Disorder-7 item; IQR=interquartile range; KG=kilogram; PHQ=Patient Health Questionnaire; SD=standard deviation; SF-36=Short Form Health Survey-36 item; TMS=transcranial magnetic stimulation; WSAS=Work & Social Adjustment Scale

For peer review only

# CONSORT 2010 checklist of information to include when reporting a randomised trial*

| Section/Topic | Item No | Checklist item | Reported on page No |
|---|---|---|---|
| **Title and abstract** | | | |
| | 1a | Identification as a randomised trial in the title | 1 |
| | 1b | Structured summary of trial design, methods, results, and conclusions (for specific guidance see CONSORT for abstracts) | 2 |
| **Introduction** | | | |
| Background and objectives | 2a | Scientific background and explanation of rationale | 4-5 |
| | 2b | Specific objectives or hypotheses | 5 |
| **Methods** | | | |
| Trial design | 3a | Description of trial design (such as parallel, factorial) including allocation ratio | 6 |
| | 3b | Important changes to methods after trial commencement (such as eligibility criteria), with reasons | 10-11 |
| Participants | 4a | Eligibility criteria for participants | 6-7 |
| | 4b | Settings and locations where the data were collected | 6 |
| Interventions | 5 | The interventions for each group with sufficient details to allow replication, including how and when they were actually administered | 8-10 |
| Outcomes | 6a | Completely defined pre-specified primary and secondary outcome measures, including how and when they were assessed | 11-12 |
| | 6b | Any changes to trial outcomes after the trial commenced, with reasons | NA |
| Sample size | 7a | How sample size was determined | 13 |
| | 7b | When applicable, explanation of any interim analyses and stopping guidelines | NA |
| Randomisation: | | | |
| Sequence generation | 8a | Method used to generate the random allocation sequence | 12 |
| | 8b | Type of randomisation; details of any restriction (such as blocking and block size) | 12 |
| Allocation concealment mechanism | 9 | Mechanism used to implement the random allocation sequence (such as sequentially numbered containers), describing any steps taken to conceal the sequence until interventions were assigned | 12 |
| Implementation | 10 | Who generated the random allocation sequence, who enrolled participants, and who assigned participants to interventions | |
| Blinding | 11a | If done, who was blinded after assignment to interventions (for example, participants, care providers, those | 12 |



For peer review only - http://bmjopen.bmj.com/site/about/guidelines.xhtml

For peer review only

| | | | |
|---|---|---|---|
| | | assessing outcomes) and how | |
| | 11b | If relevant, description of the similarity of interventions | 8-10 |
| Statistical methods | 12a | Statistical methods used to compare groups for primary and secondary outcomes | 13-14 |
| | 12b | Methods for additional analyses, such as subgroup analyses and adjusted analyses | NA |
| **Results** | | | |
| Participant flow (a diagram is strongly recommended) | 13a | For each group, the numbers of participants who were randomly assigned, received intended treatment, and were analysed for the primary outcome | 17-18 (Fig 1) |
| | 13b | For each group, losses and exclusions after randomisation, together with reasons | 17-18 (Fig 1) |
| Recruitment | 14a | Dates defining the periods of recruitment and follow-up | 6, 18 |
| | 14b | Why the trial ended or was stopped | 18 |
| Baseline data | 15 | A table showing baseline demographic and clinical characteristics for each group | 16-17 |
| Numbers analysed | 16 | For each group, number of participants (denominator) included in each analysis and whether the analysis was by original assigned groups | 17-18 (Fig 1) |
| Outcomes and estimation | 17a | For each primary and secondary outcome, results for each group, and the estimated effect size and its precision (such as 95% confidence interval) | 20-21 (Fig 2) (Supplementary File 2) |
| | 17b | For binary outcomes, presentation of both absolute and relative effect sizes is recommended | NA |
| Ancillary analyses | 18 | Results of any other analyses performed, including subgroup analyses and adjusted analyses, distinguishing pre-specified from exploratory | NA |
| Harms | 19 | All important harms or unintended effects in each group (for specific guidance see CONSORT for harms) | 19 |
| **Discussion** | | | |
| Limitations | 20 | Trial limitations, addressing sources of potential bias, imprecision, and, if relevant, multiplicity of analyses | 25-26 |
| Generalisability | 21 | Generalisability (external validity, applicability) of the trial findings | 25-26 |
| Interpretation | 22 | Interpretation consistent with results, balancing benefits and harms, and considering other relevant evidence | 21-26 |
| **Other information** | | | |
| Registration | 23 | Registration number and name of trial registry | 3 |
| Protocol | 24 | Where the full trial protocol can be accessed, if available | NA |
| Funding | 25 | Sources of funding and other support (such as supply of drugs), role of funders | 27 |

*We strongly recommend reading this statement in conjunction with the CONSORT 2010 Explanation and Elaboration for important clarifications on all the items. If relevant, we also recommend reading CONSORT extensions for cluster randomised trials, non-inferiority and equivalence trials, non-pharmacological treatments, herbal interventions, and pragmatic trials. Additional extensions are forthcoming: for those and for up to date references relevant to this checklist, see www.consort-statement.org.



For peer review only - http://bmjopen.bmj.com/site/about/guidelines.xhtml

BMJ Open

# Trial Of Neurostimulation In Conversion Symptoms (‘TONICS’): a feasibility randomised controlled trial of transcranial magnetic stimulation for functional limb weakness

| | |
|---|---|
| Journal: | *BMJ Open* |
| Manuscript ID | bmjopen-2020-037198.R1 |
| Article Type: | Original research |
| Date Submitted by the Author: | 17-Jun-2020 |
| Complete List of Authors: | Pick, Susannah; Institute of Psychiatry Psychology and Neuroscience<br>Hodsoll, John ; Institute of Psychiatry Psychology and Neuroscience<br>Stanton, Biba; Institute of Psychiatry Psychology and Neuroscience; King's College Hospital NHS Foundation Trust<br>Eskander, Amy; King's College Hospital NHS Foundation Trust<br>Stavropoulos, Ioannis; King's College Hospital NHS Foundation Trust<br>Samra, Kiran; King's College Hospital NHS Foundation Trust<br>Bottini, Julia; King's College Hospital NHS Foundation Trust<br>Ahmad, Hena; King's College Hospital NHS Foundation Trust<br>David, Anthony; University College London, Institute of Mental Health<br>Purves, Alistair; King's College Hospital NHS Foundation Trust<br>Nicholson, Timothy; Institute of Psychiatry Psychology and Neuroscience |
| <b>Primary Subject Heading</b>: | Neurology |
| Secondary Subject Heading: | Mental health |
| Keywords: | Adult neurology < NEUROLOGY, Adult psychiatry < PSYCHIATRY, Clinical trials < THERAPEUTICS |
| | |

SCHOLARONE™
Manuscripts

*I, the Submitting Author has the right to grant and does grant on behalf of all authors of the Work (as defined in the below author licence), an exclusive licence and/or a non-exclusive licence for contributions from authors who are: i) UK Crown employees; ii) where BMJ has agreed a CC-BY licence shall apply, and/or iii) in accordance with the terms applicable for US Federal Government officers or employees acting as part of their official duties; on a worldwide, perpetual, irrevocable, royalty-free basis to BMJ Publishing Group Ltd ("BMJ") its licensees and where the relevant Journal is co-owned by BMJ to the co-owners of the Journal, to publish the Work in this journal and any other BMJ products and to exploit all rights, as set out in our licence.*

*The Submitting Author accepts and understands that any supply made under these terms is made by BMJ to the Submitting Author unless you are acting as an employee on behalf of your employer or a postgraduate student of an affiliated institution which is paying any applicable article publishing charge ("APC") for Open Access articles. Where the Submitting Author wishes to make the Work available on an Open Access basis (and intends to pay the relevant APC), the terms of reuse of such Open Access shall be governed by a Creative Commons licence – details of these licences and which Creative Commons licence will apply to this Work are set out in our licence referred to above.*

*Other than as permitted in any relevant BMJ Author's Self Archiving Policies, I confirm this Work has not been accepted for publication elsewhere, is not being considered for publication elsewhere and does not duplicate material already published. I confirm all authors consent to publication of this Work and authorise the granting of this licence.*

For peer review only

# Trial Of Neurostimulation In Conversion Symptoms ('TONICS'): a feasibility randomised controlled trial of transcranial magnetic stimulation for functional limb weakness

**Susannah Pick[1], John Hodsoll[1], Biba Stanton[1,2], Amy Eskander[2], Ioannis Stavropoulos[2], Kiran Samra[2], Julia Bottini[2], Hena Ahmad[2], Anthony S. David[3], Alistair Purves[2], & Timothy R. Nicholson[1‡]**

[1]Institute of Psychiatry, Psychology & Neuroscience, King's College London, London, UK
[2] King's College Hospital NHS Foundation Trust, London, UK
[3]Institute of Mental Health, University College London, London, UK

‡corresponding author
Dr Timothy Nicholson. Email: timothy.nicholson@kcl.ac.uk; Tel: 0207 848 5136

Word count: 5022
Tables: 2
Figures: 2
Supplementary files: 4
Keywords: functional neurological disorder, conversion disorder, transcranial magnetic stimulation, functional motor, trial, treatment

For peer review only



For peer review only - http://bmjopen.bmj.com/site/about/guidelines.xhtml

## ABSTRACT

**Objectives:** Transcranial magnetic stimulation (TMS) has been used therapeutically for functional (conversion) motor symptoms but there is limited evidence for its efficacy and the optimal protocol. We examined the feasibility of a novel randomised controlled trial (RCT) protocol of TMS to treat functional limb weakness.

**Design:** A double-blind (patient, outcome assessor) two parallel-arm, controlled RCT.

**Setting:** Specialist neurology and neuropsychiatry services at a large National Health Service Foundation Trust in London, UK.

**Participants:** Patients with DSM-5 diagnosis of functional limb weakness. Exclusion criteria included comorbid neurological or major psychiatric disorder, contraindications to TMS, or previous TMS treatment.

**Interventions:** Patients were randomised to receive either active (single-pulse TMS to primary motor cortex (M1) above resting motor-threshold) or inactive treatment (single-pulse TMS to M1 below resting motor-threshold). Both groups received two TMS sessions, four weeks apart.

**Outcome measures:** We assessed recruitment, randomisation, and retention rates. The primary outcome was patient-rated symptom change (Clinical Global Impression–Improvement scale, CGI-I). Secondary outcomes included clinician-rated symptom change, psychosocial functioning, and disability. Outcomes were assessed at baseline, both TMS visits and at 3-month follow-up.

**Results:** Twenty-two patients were recruited and twenty-one (96%) were successfully randomised (active=10; inactive=11). Nineteen (91%) patients were included at follow-up (active=9; inactive=10). Completion rates for most outcomes were good (80-100%). Most patients were satisfied/very satisfied with the trial in both groups, although ratings were higher in the inactive arm (active=60%, inactive=92%). Adverse events were not more common for the active treatment. Treatment effect sizes for patient-rated CGI-I scores were small-moderate (Cliff's delta= -0.1-0.3, CIs= -0.79-0.28), reflecting a more positive outcome for the active treatment (67% and 44% of active arm rated symptoms as 'much improved' at session 2 and follow-up respectively, versus 20% inactive group). Effect sizes for secondary outcomes were variable.




For peer review only - http://bmjopen.bmj.com/site/about/guidelines.xhtml

**Conclusions:** Our protocol is feasible. The findings suggest that supra-motor threshold TMS of M1 is safe, acceptable and potentially beneficial as a treatment for functional limb weakness. A larger RCT is warranted.

**Trial registration:** ISRCTN51225587

**ARTICLE SUMMARY**

**Strengths and limitations of this study**

- The study examined the feasibility of a novel, controlled TMS protocol for treating functional limb weakness.
- The TMS protocol has potential to inform the minimal dose required and mechanism of action for positive outcomes in this population.
- Both patients and outcome assessors were blind to treatment allocation, but it was not possible to blind the delivery of the treatment.
- As this was a feasibility study with a small sample size, randomisation might not have adequately balanced group differences across the treatment arms.

For peer review only

## BACKGROUND

Functional neurological disorder (FND) is defined by neurological symptoms that are incompatible with other medical/neurological diagnoses [1]. FND can resemble any neurological disorder, with seizures, motor (e.g., limb weakness, tremor, dystonia, myoclonus) and sensory (visual, auditory, somatosensory) symptoms predominating. Quality of life and prognosis are often poor [2-4]. Despite recent developments in detection and diagnosis of the disorder [5], there is still a marked paucity of evidence-based, accessible treatment options. There is emerging evidence for the efficacy of some treatment modalities (e.g., specialist physiotherapy for motor symptoms or cognitive behavioural therapy for seizures) [6-9], but availability is currently limited. The development of alternative treatment options that are safe, cost-effective, acceptable to patients and accessible is critical for improving outcomes in this population.

Transcranial magnetic stimulation (TMS) has been explored as a potential treatment option for functional motor symptoms and there is accumulating evidence for its efficacy and safety from uncontrolled studies and five randomised controlled trials (RCTs) [10-15]. These studies used divergent methods and so the optimal protocol is presently unclear, for example, whether to use single pulse (spTMS) or repetitive (rTMS) stimulation; which brain region to target; how many sessions are needed; and the optimal control intervention. Previous studies have generally found post-intervention functional motor symptom improvements following stimulation of primary motor cortex (M1) [11-15]. However, few of these RCTs reported gains in other important outcomes (e.g., comorbid psychological/physical symptoms, quality of life/global functioning, healthcare resource use). Despite post-treatment



For peer review only - http://bmjopen.bmj.com/site/about/guidelines.xhtml

improvements in core FND symptoms following rTMS to M1, Taib et al. [14] for example, did not observe superior improvements in health-related quality of life (SF-36 vitality/general health) for active rTMS relative to sham-TMS, and no improvements were observed in psychological symptoms. Similarly, McWhirter et al. [15] reported improvements in subjective symptoms immediately following spTMS of M1 relative to standard care, but no associated improvements in self-reported mental or physical health (SF-12) or clinician-rated disability (Modified Rankin Scale).

Further research is therefore needed to optimise both TMS treatment and RCT protocols to enable more definitive testing of the efficacy of TMS in improving functional motor symptoms themselves as well as other important outcome domains [16, 17].

## OBJECTIVES

We aimed to explore the feasibility and acceptability of a novel, controlled spTMS protocol for functional limb weakness, to inform the design and implementation of a subsequent larger-scale RCT. The protocol consisted of a minimal ‘dose’, consisting of two brief sessions of spTMS to M1, with the target region tailored to the specific limb weakness reported by each patient. We compared active stimulation delivered above resting motor threshold (RMT) to a control condition consisting of exactly the same procedures delivered below RMT. We hypothesised that this protocol would be feasible in terms of the following key parameters: recruitment rates, acceptance of randomisation, tolerance of the intervention, successful blinding and completion of outcome measures. We also aimed to estimate the variability of outcome measures and treatment effect sizes to inform design of the next RCT.



For peer review only - http://bmjopen.bmj.com/site/about/guidelines.xhtml

## METHODS

### Trial design

The study was a double-blind two parallel arm controlled feasibility RCT of tailored spTMS to M1 in patients with functional limb weakness. The primary outcome was patient-rated symptom change. We also measured a range of other relevant secondary outcome domains to assess their feasibility and acceptability in this population (outlined below).

### Study setting and participants

Ethical approval was received from the London-Stanmore Research Ethics Committee, UK (ref:17/LO/0410). Patients with functional limb weakness were recruited from inpatient and outpatient neurology and neuropsychiatry services across the King's Health Partners (National Health Service, UK), including King's College Hospital, Guy's and St Thomas' Hospital, and the South London and Maudsley NHS Foundation Trusts. Recruitment took place between October 2017 and March 2018.

Inclusion criteria were:

- DSM-5 diagnosis of functional neurological disorder confirmed by a consultant neuropsychiatrist or neurologist
- Motor symptoms defined by functional weakness of at least one limb
- 18 years old or older
- Capacity to consent



For peer review only - http://bmjopen.bmj.com/site/about/guidelines.xhtml

Exclusion criteria were:

- Epilepsy (or considered high risk of epilepsy from medical history)
- Other contraindication to TMS (e.g. cochlear implants, metallic intracranial clips or intracranial surgery in last 12 months)
- Comorbid neurological condition (e.g. multiple sclerosis, stroke)
- Pain as primary symptom
- Previous treatment with TMS (for any condition)
- Non-fluent English speakers (if unable to accurately complete self-report questionnaires)
- Major mental health disorder: current diagnosis of schizophrenia or bipolar disorder; current drug/alcohol dependence
- History of factitious disorder
- Currently involved in another trial

Preliminary eligibility screening was completed by clinical neurology and neuropsychiatry staff. When patients were considered potentially eligible, Participant Information Sheets were provided (Supplementary File 1), and permission sought for the research team (TN/SP) to contact the patient. When permission was granted, a member of the research team subsequently contacted the patient to answer any questions and arrange an initial screening assessment visit, if the patient wished to enrol. Written informed consent was obtained at the initial screening visit, after the study had been explained in full and any remaining questions answered. All participants were told that TMS had shown promising results in previous small-scale research studies and that the current study was aiming to test the treatment more



For peer review only - http://bmjopen.bmj.com/site/about/guidelines.xhtml

stringently. Hypotheses regarding the possible mechanisms of treatment were not disclosed. Possible side effects of the treatment were outlined (e.g., headaches, scalp tingling).

Participants were not reimbursed for involvement in the study, but assistance with travel arrangements and expenses was provided, as necessary.

**Patient and Public Involvement**

A specialist service user advisory group was set up to inform the design and conduct of the study. Key national and international patient groups are involved in the dissemination plans.

**Background/screening measures**

At the initial screening visit, demographic details and medical history were obtained and a formal psychiatric screening tool administered (MINI International Neuropsychiatric Interview)[18]. Additional background measures were administered, including a personality disorder screen (Standardised Assessment of Personality – Self-Report, SAPAS-SR)[19], a measure of estimated intellectual functioning (National Adult Reading Test, NART)[20], and a trauma inventory (Childhood Experiences of Care and Abuse Questionnaire, CECA-Q)[21].

**Intervention**

Participants were randomised to receive active or inactive TMS, as described below. Both groups received two TMS sessions, separated by approximately 4 weeks. A formal script was not used during the sessions, but care was taken to have a



For peer review only - http://bmjopen.bmj.com/site/about/guidelines.xhtml

consistent and neutral approach in terms of patient interactions regarding potential improvements to minimise and standardise placebo effect.

### ***Active TMS***

The active treatment consisted of spTMS delivered to M1 including stimuli above resting motor threshold (RMT), thereby causing observable movement of the target limb. The target limb was determined for each participant, defined as the weakest limb (i.e. arm or leg on either side) that caused most significant functional impairment in daily life. The target limb remained unchanged throughout both treatment sessions. The treatment was delivered in 2 phases:

*Phase 1: Measuring resting motor threshold (RMT)*

Single pulses were delivered with a Magstim 200 (Magstim, Whitland U.K.) TMS machine either using a circular coil to the area of M1 corresponding to the hand region of both the symptomatic and non-symptomatic arms, or using a double cone coil to deliver pulses to the M1 area for the legs (for participants with leg weakness only). As double cone coils cannot target left or right legs separately (M1 for both legs are stimulated) the same procedure was repeated twice as if targeting each side individually so that the procedure was the same for legs as it was for arms.

Pulses started at 20% of machine output and increased at increments of 5% until the evoked response (measured by surface electromyography in the first dorsal interosseous of the hand or extensor digitorum brevis of the foot) exceeded 50mcV in 50% of trials using a standardised protocol which allows electromyographic



For peer review only - http://bmjopen.bmj.com/site/about/guidelines.xhtml

detection of RMT at 5-10% of TMS output, below that which will produce a movement of the limb detectable by the patient [22]. This value was recorded as the RMT. As a variable number of pulses was needed to establish RMT in each patient, further pulses were then delivered at an interval of 5-10 seconds so that a total of 100 stimuli were delivered (50 stimuli to the same region of M1 bilaterally), to ensure that all participants received an equal number of stimuli during this phase.

*Phase 2: Supra-threshold (Active) TMS*

A further 20 pulses, again at an interval of 5-10 seconds, were delivered at 120% of RMT, applied to the region of M1 corresponding to the participant's weakest limb. No deliberate effort was made by the TMS deliverer to draw attention to the movement of the target limb. A total of 120 pulses were delivered during each of the two treatment sessions. The total number of 120 stimuli was adopted because 100 stimuli is the minimum required to reliably measure RMT and an extra 20 stimuli were needed for clear supra-threshold stimulation for therapeutic effect. This number has been recommended in standardised protocols for RMT measurement [22].

***Inactive (control) TMS***

The inactive control treatment consisted of spTMS delivered to M1 that was always below RMT, thereby not leading to observable movement of the target limb. Phase 1 was identical to the procedures outlined above for measuring RMT.

*Phase 2: Sub-motor threshold (inactive) TMS*

A further 20 pulses at 80% of RMT were applied to the region of M1 corresponding to the patient's weakest limb. Whilst this constituted 'real' TMS, these stimuli did not



For peer review only - http://bmjopen.bmj.com/site/about/guidelines.xhtml

produce any limb movement. Therefore, the key difference between the treatment conditions was whether stimulation was delivered above or below RMT and the initiation of automatic limb movement or not, respectively. As with the active treatment, a total of 120 stimuli were delivered during each TMS session.

**Changes to protocol during trial**

The original protocol specified that the second TMS session would follow the first within a narrowly defined period (30 +/- 2 days); however, during the course of the trial it became clear that this was too restrictive and therefore not practicable, so the time period permitted between treatment sessions was extended (TMS session 2 to occur 28-50 days after TMS session 1).

**Outcome measures**

Outcome measures were completed before and/or after the first TMS session (baseline), before and/or after the second TMS session and three-months after the first TMS session. The primary outcome measure was patient-rated symptom change assessed with the Clinical Global Impression Improvement (CGI-I) scale [23], given the emerging consensus that patient-rated, subjective symptom improvements are particularly meaningful outcomes in this disorder [16, 17].

A range of secondary outcome measures was also included to assess the feasibility of measuring other relevant outcome domains in this group:

- outcome-rater and carer assessed symptom change (CGI-I scale)
- manual muscle testing (MRC strength scale performed by neurologist)



For peer review only - http://bmjopen.bmj.com/site/about/guidelines.xhtml

- dynamometry (if upper limb weakness present)
- subjective ratings of strength (0-100%) and weakness (1-5) in the weakest/target limb
- somatic symptoms (Patient Health Questionnaire (PHQ)-15) [24]
- depression (PHQ-9) [25]
- overall psychological distress (Core Outcomes in Routine Evaluation – 10, CORE-10) [26]
- quality of life (Short-Form Health Survey – 36, SF-36) [27]
- anxiety (Generalised Anxiety Disorder Questionnaire – 7 item, GAD-7) [28]
- disability / physical functioning (Barthel Index / Functional Independence Measure and Functional Assessment Measure (FIM/FAM) [29, 30]
- social and occupational functioning (Work and Social Adjustment Scale, WSAS) [31]
- healthcare utilisation (Client Service Receipt Inventory, CSRI) [32]

**Randomisation and blinding**

Randomisation occurred after the initial screening visit, once eligibility and consent had been confirmed. Randomisation was carried out online by the King's Clinical Trials Unit (KCTU) at the Institute of Psychiatry, Psychology and Neuroscience (IoPPN), using block randomisation. Computer-generated randomisation was initiated when the trial outcome-rater (SP) entered the initials and date-of-birth of the participant onto an online system. Randomisation was then conducted automatically and a confidential email with treatment allocation (active or inactive) sent directly to the TMS deliverer (TN). The outcome-rater (SP) remained blind to treatment allocation throughout the study, as did participants.

For peer review only

After completion of all study visits for each participant, blinding of the outcome-rater and participant were tested with a forced-choice question about which treatment the patient had received (active or inactive). The patient and outcome-rater answered the question independently. At the end of the study, participants were unblinded individually by the Principal Investigator (TN) during debriefing, with the outcome-rater absent from the room. The outcome-rater remained blind to treatment allocation until all outcome data analyses were completed by the trial statistician.

## Safety monitoring

Adverse events (AEs) were monitored and recorded at each study visit and reported to the Principal Investigator (TN) or Trial Steering Committee as appropriate. Patients were invited to contact the research team at any time during the trial, in case of an AE occurring between visits.

## Statistical analysis

### *Sample size determination*

Published data on TMS in FND indicates an improvement rate of approximately 10%, albeit on the basis of uncontrolled data. As spontaneous recovery rates are very low, a 10% improvement rate in the control arm at 1 month would be a conservative figure. From a previous CBT trial in FND [7], we would expect 30% of eligible patients to decline participation and then 10% to not complete treatment. Hence with alpha=0.05 and 90% power, to detect an improvement rate of 80% in the active treatment arm relative to 10% in the control (z test between two independent proportions), 9 patients would be needed per arm. For 18 patients to complete the



For peer review only - http://bmjopen.bmj.com/site/about/guidelines.xhtml

study, given a 10% drop out, we would need to randomise 20 participants (30 consented). This allows 10% dropout rate to be assessed with an expected 95% CI of 0% to 24%.

***Feasibility parameters***

Data analysis was carried out in R (v.3.2) by the blinded trial statistician (JH) and adopted the intention-to-treat (ITT) principle. The aims of the analysis were to examine trial feasibility parameters as follows:

- recruitment, randomisation and loss to follow-up rates
- tolerance of treatment, safety, treatment fidelity, participant / outcome-rater blinding and patient satisfaction
- estimate treatment effect sizes as potential outcomes of future trials

The analysis primarily consisted of descriptive statistics to summarise the rates of consent and randomisation of eligible patients, study retention, data quality (i.e., completion of outcome measures, missing data) and the acceptability of TMS to the patient population. Participant demographic and clinical characteristics were also described at baseline.

To assess improvement in symptoms, estimates of treatment effect sizes and 95% confidence intervals on the primary outcome measure (patient-rated CGI-I scale) were obtained using Cliff's Delta as this scale is ordinal. Cliff's Delta is a functional equivalent to Cohen's d for ordinal data, which does not make assumptions about



For peer review only - http://bmjopen.bmj.com/site/about/guidelines.xhtml

the shape or spread of the distribution. In this analysis, Cliff's delta represents the mean between-group difference of within-group change. The effect size values can be interpreted as reflecting the number of times a value in one distribution (active group) is higher than the value in the other distribution (inactive group). Criteria for interpreting the effect size were given by Romano et al. [33], with delta < 0.147 being negligible, delta < 0.33 small, delta < 0.474 being medium and otherwise large. For the secondary outcomes, descriptive statistics and effect sizes were calculated as appropriate for the type of data. Effect sizes (and 95% CI) for secondary outcomes were presented as Cohen's d or Cliff's delta as appropriate to the outcome data.

## RESULTS

### Sample characteristics

#### *Demographics*

The demographic characteristics of participants at enrolment to the study are displayed in Table 1. The average age in each group was similar and the majority of participants in both groups was female, right-handed, married/cohabiting, and most often of white or black British ethnicity. Participants were most likely to report holding an undergraduate degree or vocational qualification. Participants were most often unemployed, but a proportion of patients reported being retired due to ill-health or employed full-time.

#### *Background / clinical characteristics*

Table 2 shows key background and clinical features of participants at entry to the study. The MINI screen identified one patient with possible current psychosis, who



For peer review only - http://bmjopen.bmj.com/site/about/guidelines.xhtml

was subsequently excluded and referred to appropriate clinical services. In eligible patients, the most common comorbid mental health diagnoses identified were major depressive disorder (n=8, 38%) and post-traumatic stress disorder (n=6, 29%). A larger proportion of the inactive group reported additional FND symptoms (i.e., other than limb weakness), relative to the active group. The duration of time since diagnosis was longer for the inactive group, although the duration since symptom onset was similar across groups. A similar proportion of patients in each group reported concurrent interventions at entry to the study and the average number of medications taken was approximately equal. Full details of concomitant treatments are provided in Supplementary File 2. All participants in both groups were taking medication at every time point, with the most common medications being antidepressant, anti-epileptic, anxiolytic and analgesic. The second most common intervention received was physiotherapy (outpatient or during inpatient hospital stays). A small proportion of participants received additional input from occupational therapy, psychology, psychiatry, specialist neurorehabilitation or inpatient hospital (general/neurology) services during the trial.

For peer review only

## Table 1 – Participant demographic characteristics

| | | Active TMS (N=10) | Inactive TMS (N=11) |
|---|---|---|---|
| **Age (Median, interquartile range)** | | 38 (32.5, 46.5) | 41 (33.5, 51) |
| **Gender** | **Female** | 8 (80) | 10 (90.9) |
| | **Male** | 2 (20) | 1 (9.1) |
| **Marital Status** | **Single** | 5 (50) | 3 (27.3) |
| | **Cohabiting / Married** | 5 (50) | 7 (63.6) |
| | **Separated / Divorced** | 0 (0) | 1 (9.1) |
| **Qualifications** | **None** | 0 (0) | 1 (9.1) |
| | **GCSE** | 4 (40) | 1 (9.1) |
| | **A Levels** | 1 (10) | 0 (0) |
| | **Graduate** | 3 (30) | 3 (27.3) |
| | **Postgraduate** | 0 (0) | 1 (9.1) |
| | **Vocational** | 2 (20) | 5 (45.5) |
| **Employment** | **Full Time** | 1 (10) | 3 (27.3) |
| | **Part Time** | 2 (20) | 0 (0) |
| | **Unemployed** | 7 (70) | 4 (36.4) |
| | **Retired (ill health)** | 0 (0) | 4 (36.4) |
| **Handedness** | **Right** | 8 (80) | 8 (72.7) |
| | **Left** | 2 (20) | 2 (18.2) |
| | **Ambidextrous** | 0 (0) | 1 (9.1) |
| **Ethnicity** | **White British** | 5 (50) | 7 (63.6) |
| | **Irish** | 1 (10) | 0 (0) |
| | **White and Black Caribbean** | 0 (0) | 1 (9.1) |
| | **Mixed** | 1 (10) | 0 (0) |
| | **Black British** | 2 (20) | 2 (18.2) |
| | **Caribbean** | 0 (0) | 1 (9.1) |
| | **Other** | 1 (10) | 0 (0) |

**Key:** TMS=transcranial magnetic stimulation

For peer review only

**Table 2 – Background/clinical characteristics by treatment group**

| | Active TMS (n=10) | Inactive TMS (n=11) |
|---|---|---|
| **SAPAS-SR Total scores (median, IQR)** | 3 (2, 4.8) | 3 (2, 4) |
| **NART estimated IQ scores (median, IQR)** | 107 (105, 113) | 108 (108, 112) |
| **Psychiatric comorbidity present (baseline) (n, %)** | 6 (60) | 5 (45.5) |
| **Other FND symptoms (baseline) (n, %)** | 5 (50) | 9 (81.8) |
| **Age at FND onset, years (median, IQR)** | 35 (28.25, 45) | 31 (23.5, 48.5) |
| **Duration of FND, months (baseline) (median, IQR)** | 41 (14.75 ,63) | 42 (37, 107.5) |
| **Duration since FND diagnosis, months (baseline) (median, IQR)** | 1 (0, 5.25) | 12 (0.5, 38.5) |
| **Number of current medications (median, IQR)** | | |
| **Baseline** | 3 (2.25, 11) | 4 (3.5, 6) |
| **TMS session 1** | 3 (2, 11) | 4 (3.5, 6.5) |
| **TMS session 2** | 7 (2.25, 12.5) | 4.5 (3.25, 6.5) |
| **Follow-up** | 3 (2, 12) | 5 (3.5, 7) |
| **Concurrent treatments (n, %)** | | |
| **Baseline** | 10 (100) | 10 (100) |
| **TMS session 1** | 10 (100) | 9 (100) |
| **TMS session 2** | 6 (100) | 8 (100) |
| **Follow-up** | 9 (100) | 9 (100) |

**Key:** FND=functional neurological disorder; IQR=interquartile range; MDD=major depressive disorder; MINI=MINI International Neuropsychiatric Interview; NART=National Adult Reading Test; PTSD=post-traumatic stress disorder; SAPAS-SR=Standardised Assessment of Personality Abbreviated Scale–Self-Report; TMS=transcranial magnetic stimulation

## Feasibility parameters

Figure 1 displays rates of recruitment, treatment allocation, completion of the study and participants included in the data analysis (CONSORT flow diagram).

<insert Figure 1>

### *Recruitment, attendance and completion*

Of 32 potential candidates referred to the study, 22 consented to participate. Of these, 21 were found to be eligible at baseline screening. All 21 eligible patients were randomised and attended the first TMS treatment session. A total of five



For peer review only

patients did not attend the second TMS session (active=4; inactive=1), none gave reasons directly related to the intervention (Figure 1). At follow-up, two patients did not attend (active=1; inactive=1). Recruitment of the target number of participants (n=20) was completed within six months. The final follow up session took place approximately nine months after commencement of the study.

### *Data quality*

For each visit, the percentage return for each outcome measure was calculated in relation to the number of patients who attended that session (Supplementary File 3). Completion rates for the primary outcome measure (patient-rated CGI-I scale) was 100% at all timepoints. For most other measures, return rates were between 90-100% (i.e., outcome-rater CGI-I scale, Barthel Index, GAD-7, PHQ-9, PHQ-15, WSAS, CORE-10, most SF-36 subscales). A small number of scales were completed less consistently, although rates were still above 80% (e.g., SF-36 Role Emotional at TMS session 1, patient strength ratings/dynamometry at follow-up). Two measures were completed infrequently (carer-rated CGI-I scale/FIM-FAM) in 25% or fewer of the attendees at each timepoint.

### *Blinding*

There were no unexpected compromises to blinding during the study procedures. When asked with a forced-choice question at the end of the study, the active treatment was more likely to be correctly guessed as active by both patients (40%) and the outcome-rater (50%), compared to the inactive treatment (patients=36%; outcome-rater=27%). The percentage of correct responses by either informant was not above chance.



For peer review only - http://bmjopen.bmj.com/site/about/guidelines.xhtml
For peer review only

### *Patient satisfaction*

Patients' ratings of their overall experiences of the trial were good. The majority of patients (76%) stated that they were either 'somewhat' or 'very' satisfied with the trial, although ratings were higher in the inactive group (active=60%, inactive=92%). None of the patients in either group reported being 'unsatisfied' (neither 'somewhat' nor 'very'). Qualitatively, patients reported feeling pleased with the level of support and information provided by the research team, felt valued, found assistance with travel arrangements beneficial, and were pleased to be part of a study that could help people with FND more broadly. For some patients, lack of improvement and/or unwanted side effects were noted in the feedback to explain less positive satisfaction ratings (i.e., 'neither satisfied nor unsatisfied').

### *Adverse events*

There were four serious adverse events (SAEs) reported during the study (active=3; inactive=1). One SAE occurred between TMS session 1 and 2, and the other three occurred between TMS session 2 and follow-up. There were no SAEs immediately following a TMS session and none of the SAEs were considered related to the treatment by the Trial Steering Committee. In total there were 78 (non-serious) adverse events (AEs) with 15 of these occurring before the first treatment session. Following the start of treatment, there were 26 AEs in the active group and 37 in the inactive group. A proportion of patients in each group reported headaches at some time during the trial, but rates were higher in the inactive group (n=5) relative to the active group (n=3). Worsening of FND symptoms was reported by some patients in each group at one or more time point, but the frequency of such reports was higher in the inactive group (15) compared to the active group (12).



For peer review only - http://bmjopen.bmj.com/site/about/guidelines.xhtml

**Primary outcome: patient-rated CGI-I scores**

Figure 2 displays the patient-rated CGI-I scores by group. Immediately prior to TMS session 1, 1 participant (9%) in the inactive group and 0% of the active group rated their symptoms as ‘much improved’ relative to their condition at entry to the study. Immediately after TMS session 1, these ratings remained the same. Immediately prior to TMS session 2, 67% of patients in the active group and 20% in the inactive group reported that their symptoms were ‘much improved’. The relative percentage of ‘much improved’ again remained the same immediately following TMS session 2. Finally, at three-month follow-up, the number ‘much improved’ was 44% in the active group and 20% in the inactive group.

<insert Figure 2>

Effect sizes and 95% confidence intervals (Cliff’s Delta) for patient-rated CGI-I scores were calculated. The effect size was positive prior to TMS session 1 reflecting coincidentally worse ratings in the active group (Cliff’s delta=0.35 (-0.17, 0.71)). This difference remained the same immediately following TMS session 1 (Cliff’s delta=0.35 (-0.15, 0.7)). However, this pattern was reversed by TMS session 2, indicating a benefit for the active treatment with moderate effect sizes pre- (Cliff’s delta = -0.35 (-0.73, 0.19)) and post-treatment (Cliff’s delta = -0.44 (-0.79, 0.13)). At three-month follow-up there was still an advantage for the active treatment; however, the difference was smaller (Cliff’s delta = -0.2 (-0.6, 0.28)), potentially due to a relative improvement in the inactive group.



For peer review only - http://bmjopen.bmj.com/site/about/guidelines.xhtml

### Secondary outcomes

Descriptive statistics, effect sizes and confidence intervals for the secondary outcomes can be found in Supplementary File 4. There was considerable variability in the effect sizes and 95% confidence intervals for these outcomes and so the findings cannot be interpreted conclusively. However, the pattern of findings for the following outcomes suggested a benefit of active TMS: outcome-rater CGI-I scores, psychological distress (CORE-10), aspects of quality of life (SF-36 physical functioning, vitality/energy, role limitations due physical and emotional factors), activities of daily living (Barthel), primary care service use. The following outcomes did not suggest a benefit of active TMS: grip strength (dynamometry), subjective (patient-rated) limb strength, additional physical symptoms (PHQ-15), anxiety (GAD-7), depression (PHQ-9), some aspects of quality of life (SF-36 bodily pain, social functioning, mental health), social/occupational functioning (WSAS), inpatient hospital admissions and total outpatient healthcare contacts.

## DISCUSSION

This novel double-blind RCT of spTMS to M1 for the treatment of functional limb weakness was found to be feasible in terms of key parameters allowing estimation of the effect sizes for key outcome variables, and to inform the planning and implementation of a larger RCT.

### Feasibility

Rates of recruitment and retention were acceptable, with only two patients (10%) failing to complete the follow-up visit. Whilst 5 patients did not attend TMS session 2, none of these instances was directly related to the nature of the intervention.



For peer review only - http://bmjopen.bmj.com/site/about/guidelines.xhtml

Nevertheless, consideration should be given to ways of improving attendance rates at the second TMS session, such as offering the session earlier (e.g., after 1 or 2 weeks) and ensuring that any barriers to attendance are identified and managed in advance.

Completion of outcome measures was generally good with rates of 90-100% for most scales. However, the carer-rated CGI-I scale and the FIM-FAM were not completed frequently. Reasons for the lack of completion of the carer-rated CGI-I related to carers not being present or different carers attending each appointment. In future, a specific carer could be identified at the start of the study (in consultation with the patient) and ratings could be obtained by telephone, should that carer be absent at specific visits. It became clear that the FIM-FAM was not a suitable measure for this study, because it requires completion on an inpatient basis, usually by one or more members of a multidisciplinary clinical team. In this study, patients were recruited from a range of outpatient and inpatient settings, and ratings from inpatient clinical teams were at times difficult to obtain. Furthermore, several items on the measure replicated similar constructs assessed within other measures used in the trial (i.e., Barthel, SF-36).

Blinding appeared to be successful, with correct identification of active treatment below chance for both the patients and outcome-rater at the end of the study. Patient satisfaction ratings were also encouraging, suggesting that the trial procedures and the intervention were acceptable in this population. There were no SAEs directly related to the intervention and rates of potentially related AEs (i.e., headaches, FND



For peer review only - http://bmjopen.bmj.com/site/about/guidelines.xhtml

symptom worsening) were not reported at higher rates in the active group. Adverse events should be closely monitored in future studies.

**Outcomes**

***Primary outcome – patient-rated symptom improvement***

Point estimates for the patient-reported symptom improvement showed superiority for the active spTMS intervention relative to the inactive intervention, with small to moderate effect sizes. Improvements were most apparent at TMS session 2 but were still evident at follow-up. It is notable that the pattern of scores on the outcome-rater CGI-I scale concurred with the patient-rated CGI-I scores. These findings suggest that tailored spTMS, delivered above RMT to the area of M1 corresponding to a target limb (i.e., that limb which is functionally weakest) and thus causing movement of that limb, potentially could lead to greater improvements than the same intervention delivered below RMT (i.e., not inducing observable movement). These results concur with those of other studies [11-15] which have previously shown improvements in subjective or objective measures of functional motor symptom severity following spTMS or rTMS to M1.

The mechanism(s) by which TMS to M1 yields improvements in functional motor symptoms is unclear. It is possible that a neuromodulatory mechanism may operate in protocols using rTMS and/or that a general placebo effect could be responsible for improvements in cases where patients/outcome assessors are not blind to treatment allocation. However, similarly to Garcin et al. [12], our study suggests that elicitation of normal function of the weak limb with minimal doses of spTMS is sufficient to induce improvements, at least in the short-term. Induction of observable normal



For peer review only - http://bmjopen.bmj.com/site/about/guidelines.xhtml

function in the limb might result in modification of patients' beliefs and expectations regarding limb functioning and the possibility of recovery, and/or may represent a form of motor retraining effect. It is notable that the improvements did not occur immediately after the first treatment but were instead evident by the second treatment session (pre-TMS), suggesting that whilst one TMS session was sufficient to induce change, the mechanism by which change occurred required time to manifest as symptom reduction.

The findings in this study suggest that the patient-rated CGI-I scale is acceptable and sensitive to change as a measure of symptom improvement in FND intervention studies, in accordance with previous findings across treatment modalities and FND symptom types. This measure has recently been recommended as a primary outcome measure in FND treatment studies [17].

***Secondary outcomes***

High rates of completion of most of the secondary outcome measures indicated that they are appropriate tools for use in future, similar studies. Of the range of outcome domains included, the clearest trends for intervention-related improvements were in activities of daily living/disability (Barthel), overall psychological distress (CORE-10), aspects of health-related quality of life (i.e., physical functioning, physical role, vitality, emotional role) and primary care service use. Whilst extreme caution should be exercised in interpreting these findings due to the small sample size, small-moderate effect sizes and variable confidence intervals, these initial findings suggest that active spTMS might be associated with improvements in aspects of mental health, daily functioning (i.e., roles, daily activities, physical) and treatment seeking,



For peer review only - http://bmjopen.bmj.com/site/about/guidelines.xhtml

in addition to core FND symptom improvements. This extends the findings of previous studies, which have generally demonstrated improvements in functional motor symptoms only. However, it is not possible to say whether improvements in these additional outcome domains followed or preceded motor symptoms.

**Strengths and limitations**

A key strength of this study included the use of a minimal TMS protocol (two brief sessions of spTMS only), which was acceptable to patients and therefore resulted in good treatment adherence rates. This minimal TMS protocol also has potential to be used as a widely accessible treatment that could be used as adjunct to other therapies in a range of settings.

Another strength was that our inactive intervention was similar enough to the active treatment (i.e., 'real' TMS) to reduce the risk of patients inadvertently becoming unblinded to treatment allocation. Furthermore, blinding of both patients and outcome assessors ensured that post-treatment gains were not due entirely to general placebo effects. The inclusion of patients with additional functional neurological symptoms, non-major psychiatric comorbidities and those undergoing concomitant treatments yielded a sample that was representative of the broader FND patient population, improving the generalisability of the findings.

However, it is possible that the additional interventions that some patients were undergoing (e.g., physiotherapy, specialist neurorehabilitation) may have facilitated some of the improvements reported following treatment. Future RCTs with larger samples should balance the influence of concomitant treatments and/or any



For peer review only - http://bmjopen.bmj.com/site/about/guidelines.xhtml

incidental baseline between-group differences in symptoms, background features, or other relevant variables.

Another limitation to note is that some degree of improvement in FND symptoms was observed in both groups prior to commencing the first TMS session, relative to enrolment to the study. It is therefore unclear whether the improvements observed following TMS reflected the effect of the intervention (including its anticipation) or the natural course of the disorder. The lack of a formal script during treatment sessions might have led to inconsistencies in placebo effect. Future studies might valuably include an additional standard care or waiting-list control group, to examine these factors.

**Conclusion**

The findings suggest that active (supra-motor threshold) spTMS to M1 is a safe, efficient, acceptable, and potentially effective treatment for functional limb weakness, leading to improvements in core symptoms and potentially other important outcome domains. A larger, pilot RCT is now warranted, to obtain a more robust estimate of effect sizes and variability in outcomes for this promising intervention.

For peer review only

## FIGURE LEGENDS

**Figure 1 – CONSORT diagram**

**Figure 2 – Patient-rated CGI-I categories by treatment group and timepoint**

## DECLARATIONS

### Ethics approval and consent to participate

The study was reviewed and approved by the London-Stanmore NHS Research Ethics Committee - study reference number 17/LO/0410). All participants provided informed, written consent prior to involvement in the study.

### Consent for publication

Not applicable.

### Competing interests

Not applicable.

### Data availability

All data relevant to the study are included in the article or uploaded as supplementary information

### Funding

TN, SP and the study were funded by a National Institute of Health Research (NIHR) Clinician Scientist Award to TN. This article represents independent research funded by the NIHR. The views expressed are those of the authors and not necessarily those of the NHS, NIHR or the Department of Health and Social Care.



For peer review only - http://bmjopen.bmj.com/site/about/guidelines.xhtml

## Authors' contributions

TN, AP and AD developed the study design. TN wrote the ethics proposal/study protocol, recruited some participants and conducted the TMS sessions. SP recruited and screened participants, conducted baseline and all subsequent outcome assessments, cleaned/entered data, and wrote the first/subsequent drafts of the manuscript. JH conducted the statistical analyses, prepared the CONSORT flow diagram and some sections of the results. BS, KS, JB, HA, IS, and AE conducted clinical strength tests during outcome assessments. All authors contributed to editing of the manuscript for important intellectual content and approved the final version prior to submission.

For peer review only

## REFERENCES

[1] American Psychiatric Association. Diagnostic and statistical manual of mental disorders (DSM-5). 5th edn. Washington: American Psychiatric Association, 2013.

[2] Gelauff J, Stone J, Edwards M, *et al*. The prognosis of functional (psychogenic) motor symptoms: a systematic review. *J Neurol Neurosurg Psychiatry* 2014;**85**(2):220-226.

[3] Anderson KE, Gruber-Baldini AL, Vaughan CG, *et al*. Impact of psychogenic movement disorders versus Parkinson's on disability, quality of life, and psychopathology. *Mov Disord* 2007;**22**(15):2204-2209.

[4] Szaflarski JP, Hughes C, Szaflarski M, *et al*. Quality of life in psychogenic nonepileptic seizures. *Epilepsia* 2003;**44**(2):236-242.

[5] Espay A.J., Aybek S., Carson A., *et al*. Current concepts in diagnosis and treatment of functional neurological disorders. *JAMA Neurol* 2018;**75**(9):1132-1141.

[6] LaFrance WC, Jr., Baird GL, Barry JJ, *et al*. Multicenter pilot treatment trial for psychogenic nonepileptic seizures: A randomized clinical trial. *JAMA Psychiatry* 2014;**71**(9):997-1005.

[7] Goldstein LH, Chalder T, Chigwedere C, *et al*. Cognitive-behavioral therapy for psychogenic nonepileptic seizures: a pilot RCT. *Neurology* 2010;**74**(24):1986-1994.

[8] Nielsen G, Buszewicz M, Stevenson F, *et al*. Randomised feasibility study of physiotherapy for patients with functional motor symptoms. *J Neurol Neurosurg Psychiatry* 2017;**88**(6):484-490.

[9] Jordbru AA, Smedstad LM, Klungsøyr O, *et al*. Psychogenic gait disorder: a randomized controlled trial of physical rehabilitation with one-year follow-up. *J Rehabil Med* 2014;**46**(2):181-187.

[10] Pollak TA, Nicholson TR, Edwards MJ, *et al*. A systematic review of transcranial magnetic stimulation in the treatment of functional (conversion) neurological symptoms. *J Neurol Neurosurg Psychiatry* 2014;**85**(2):191-197.

[11] Broersma M, Koops EA, Vroomen PC, *et al*. Can repetitive transcranial magnetic stimulation increase muscle strength in functional neurological paresis? A proof-of-principle study. *Eur J Neurol* 2015;**22**(5):866-873.

[12] Garcin B, Mesrati F, Hubsch C, *et al*. Impact of transcranial magnetic stimulation on functional movement disorders: cortical modulation or a behavioral effect? *Front Neurol* 2017;**8**:338.

[13] Garcin B, Roze E, Mesrati F, *et al*. Transcranial magnetic stimulation as an efficient treatment for psychogenic movement disorders. *J Neurol Neurosurg Psychiatry* 2013;**84**(9):1043-1046.



For peer review only - http://bmjopen.bmj.com/site/about/guidelines.xhtml

[14] Taib S, Ory-Magne F, Brefel-Courbon C*, et al*. Repetitive transcranial magnetic stimulation for functional tremor: A randomized, double-blind, controlled study. *Mov Disord* 2019;**34**(8):1210-1219.

[15] McWhirter L, Ludwig L, Carson A*, et al*. Transcranial magnetic stimulation as a treatment for functional (psychogenic) upper limb weakness. *J Psychosom Res* 2016;**89**:102-106.

[16] Nicholson TR, Carson AJ, Edwards MJ*, et al*. Outcome measures for functional neurological disorder – a review of the theoretical complexities. *J Neuropsychiatry Clin Neurosci* 2020;**32**(1):33-42.

[17] Pick S, Anderson D, Asadi-Pooya A*, et al*. Outcome measurement in functional neurological disorder: a systematic review and recommendations. *J Neurol Neurosurg Psychiatry* (in press). doi:10.1136/jnnp-2019-322180.

[18] Sheehan DV, Lecrubier Y, Sheehan KH*, et al*. The Mini-International Neuropsychiatric Interview (M.I.N.I.): the development and validation of a structured diagnostic psychiatric interview for DSM-IV and ICD-10. *J Clin Psychiatry* 1998;**59 Suppl 20**:22-33.

[19] Germans S, Van Heck GL, Moran P*, et al*. The Self-report Standardized Assessment of Personality-abbreviated Scale: Preliminary results of a brief screening test for personality disorders. *Personal Ment Health* 2008;**2**(2):70-76.

[20] Nelson HE. National Adult Reading Test (NART): For the assessment of premorbid intelligence in patients with dementia: Test manual. Nfer-Nelson, 1982.

[21] Bifulco A, Bernazzani O, Moran P*, et al*. The childhood experience of care and abuse questionnaire (CECA. Q): validation in a community series. *Br J Clin Psychol* 2005;**44**(4):563-581.

[22] Mills KR. Magnetic Stimulation of the Human Nervous System. Oxford: Oxford University Press 1999.

[23] Guy W. ECDEU Assessment Manual for Psychopharmacology. Rockville, MD, US: Department of Health, Education, and Welfare, 1976.

[24] Kroenke K, Spitzer RL, Williams JB. The PHQ-15: validity of a new measure for evaluating the severity of somatic symptoms. *Psychosom Med* 2002;**64**(2):258-266.

[25] Kroenke K, Spitzer RL, Williams JBW. The PHQ-9. *J Gen Intern Med* 2001;**16**(9):606-613.

[26] Barkham M, Bewick B, Mullin T*, et al*. The CORE-10: A short measure of psychological distress for routine use in the psychological therapies. *Couns Psychother Res* 2013;**13**(1):3-13.

[27] Ware Jr JE, Sherbourne CD. The MOS 36-item short-form health survey (SF-36): I. Conceptual framework and item selection. *Med Care* 1992;**30**:473-483.



For peer review only - http://bmjopen.bmj.com/site/about/guidelines.xhtml

[28] Spitzer RL, Kroenke K, Williams JB, *et al*. A brief measure for assessing generalized anxiety disorder: the GAD-7. *Arch Intern Med* 2006;**166**(10):1092-1097.

[29] Mahoney FI, Barthel DW. Functional evaluation: the Barthel Index: a simple index of independence useful in scoring improvement in the rehabilitation of the chronically ill. *Md State Med J* 1965;**14**:61-65.

[30] Turner-Stokes L, Nyein K, Turner-Stokes T, *et al*. The UK FIM FAM: development and evaluation. *Clin Rehabil* 1999;**13**(4):277-287.

[31] Mundt JC, Marks IM, Shear MK, *et al*. The Work and Social Adjustment Scale: a simple measure of impairment in functioning. *Br J Psychiatry* 2002;**180**(5):461-464.

[32] Beecham J, Knapp M. Costing psychiatric interventions. Measuring mental health needs.Gaskell, London 2001;200-224.

[33] Romano J, Kromrey JD, Coraggio J, *et al*. Exploring methods for evaluating group differences on the NSSE and other surveys: Are the t-test and Cohen'sd indices the most appropriate choices. In *Annual Meeting of the Southern Association for Institutional Research* 2006;1-51.

For peer review only



For peer review only - http://bmjopen.bmj.com/site/about/guidelines.xhtml

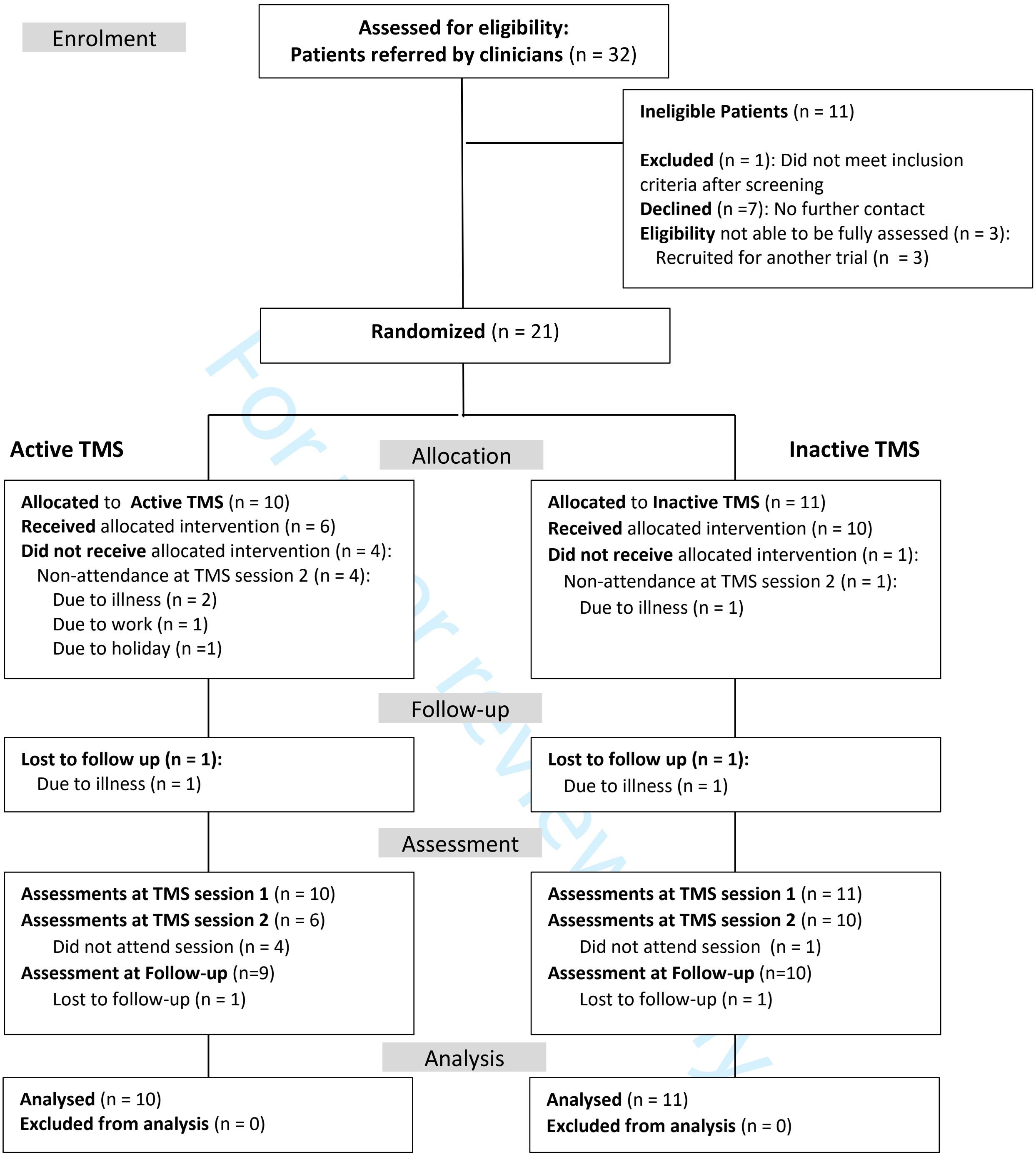
Enrolment
Assessed for eligibility:
Patients referred by clinicians (n = 32)
Ineligible Patients (n = 11)
Excluded (n = 1): Did not meet inclusion criteria after screening
Declined (n =7): No further contact
Eligibility not able to be fully assessed (n = 3):
Recruited for another trial (n = 3)
Randomized (n = 21)
Active TMS
Allocation
Inactive TMS
Allocated to Active TMS (n = 10)
Received allocated intervention (n = 6)
Did not receive allocated intervention (n = 4):
Non-attendance at TMS session 2 (n = 4):
Due to illness (n = 2)
Due to work (n = 1)
Due to holiday (n =1)
Allocated to Inactive TMS (n = 11)
Received allocated intervention (n = 10)
Did not receive allocated intervention (n = 1):
Non-attendance at TMS session 2 (n = 1):
Due to illness (n = 1)
Follow-up
Lost to follow up (n = 1):
Due to illness (n = 1)
Lost to follow up (n = 1):
Due to illness (n = 1)
Assessment
Assessments at TMS session 1 (n = 10)
Assessments at TMS session 2 (n = 6)
Did not attend session (n = 4)
Assessment at Follow-up (n=9)
Lost to follow-up (n = 1)
Assessments at TMS session 1 (n = 11)
Assessments at TMS session 2 (n = 10)
Did not attend session (n = 1)
Assessment at Follow-up (n=10)
Lost to follow-up (n = 1)
Analysis
Analysed (n = 10)
Excluded from analysis (n = 0)
Analysed (n = 11)
Excluded from analysis (n = 0)

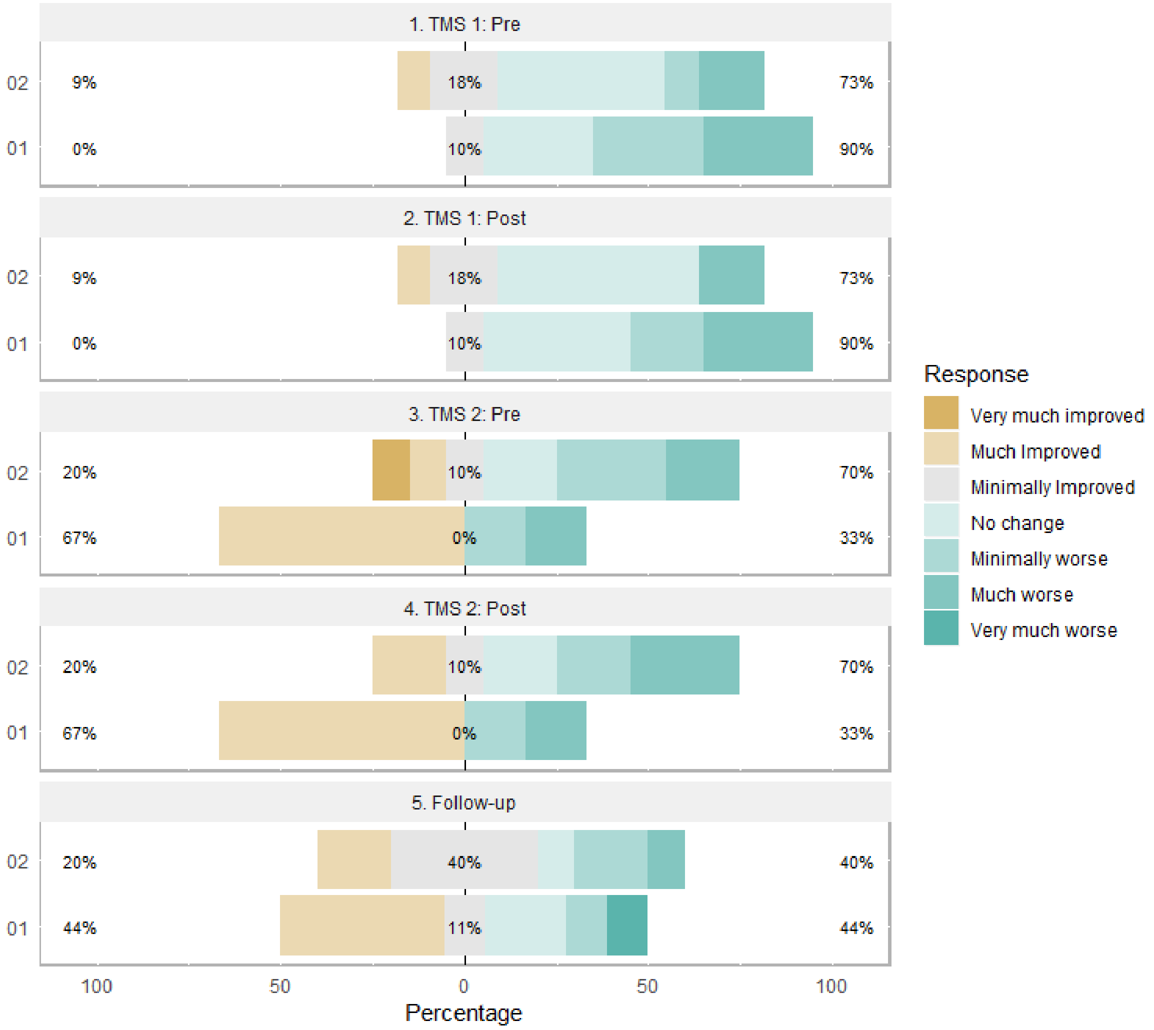


Key: 01=Active TMS; 02=Inactive TMS

**Supplementary File 1. Patient Information Sheet**

*There is no potentially identifiable patient information in this document.

# Patient Information Sheet & Consent Form

## Trial Of Neurostimulation In Conversion Symptoms (TONICS):
### A Randomised Controlled Trial (RCT) feasibility study of Transcranial Magnetic Stimulation (TMS) for conversion disorder with motor symptoms

REC reference number 17/LO/0410

You are being invited to take part in a research study.  Before you decide, please take time to read the following information and to decide whether or not you would like to participate. If anything is unclear or you would like further information, please ask a member or the research team. Thank you for taking the time to read this.

### What is the purpose of the study?

This study aims to assess Transcranial Magnetic Stimulation (TMS) as a new potential treatment for conversion disorder (CD), also known as Functional Neurological Disorder (FND).  CD is where neurological symptoms, such as weakness, occur but no structural neurological disease can be found – therefore they are disorders of function, rather than structure. There are few proven treatments for weakness that is caused by CD. However, there is encouraging preliminary evidence that TMS could be an effective and safe treatment for such symptoms but until a Randomised Controlled Trial (RCT) is conducted it is not possible to establish whether this is the case.

### What is Transcranial Magnetic Stimulation (TMS)?

TMS is a form of ‘non-invasive brain stimulation’, i.e. it is a way of stimulating the brain from outside the head. It works by holding a magnetic coil approximately the size of a small side plate against the head (it rests on the scalp) which then delivers magnetic pulses that stimulate the underlying brain. It was developed over 30 years ago and has been increasingly used treat a number of neurological and psychiatric disorders. It is considered to be a relatively safe and generally well-tolerated treatment.  This is a picture of TMS coil being used in our laboratory*:

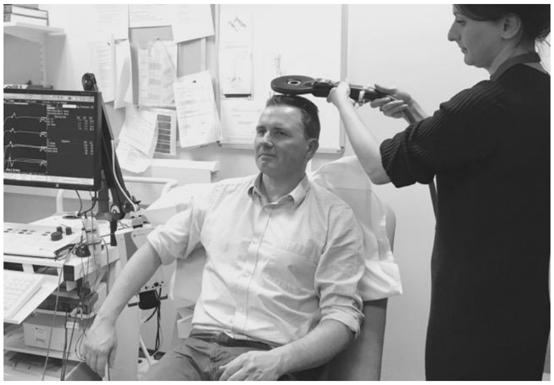

*The individuals depicted in this image are members of the research team, not clinical cases.



For peer review only

For peer review only - http://bmjopen.bmj.com/site/about/guidelines.xhtml

**What is a Randomised Controlled Trial (RCT)?**

Randomised Controlled Trials (RCTs) are the best way to tell whether a treatment really works and each year thousands of people take part in them. The word 'controlled' means that a 'control' treatment, e.g. an inactive or 'placebo' form of the treatment, is used to compare response to the 'active' treatment being investigated. This allows us to know whether any improvements (or side effects) are really due to the treatment or could either have occurred due to placebo effects or could have naturally occurred. Therefore patients are allocated to different groups to receive either the active treatment (Group A) or the inactive / placebo treatment (Group B).

The term 'randomised' means that people allocated at random to one of these two groups as this is the only way to compare treatments fairly. Randomisation means the chances are exactly equal for being allocated to either group and therefore no-one can predict in advance the group to which you will be allocated, in case this in any way affects what you or we expect to be the outcome of the study. Random allocation could be done using the result of tossing a coin (i.e. 'heads' for group A and 'tails' for Group B) to decide which treatment you will get but we will do this using a computer.

It is also important, where possible, that patients do no know (i.e. are 'blind' to) which treatment they have been allocated to as this can affect response. This means you won't know which group you've been allocated to until *after* you have not only completed the treatment but also completed the follow up interviews and questionnaires which will assess your response to the treatment you received.

For those who were allocated the 'inactive treatment' if it is felt after completing the treatment that they might benefit from receiving 'active' TMS as well, they will be offered this treatment after finishing the trial.

**Why have I been chosen?**

You have been chosen because you are over 18 and have been diagnosed with CD that is causing weakness in at least one of your limbs – this is known as 'motor' CD. As we don't currently know if TMS is any more helpful to patients than placebo, a Randomised Controlled Trial (RCT) is the most exact and fair way for us to see how helpful TMS really is at improving weakness in motor CD.

**Do I have to take part and can I withdraw from the study if I change my mind?**

It is completely up to you to decide whether or not to take part. You may consider this at your leisure, and contact us for more information, at the number below or arrange to discuss the study with a member of the research team. If you do decide to take part you will still be free to withdraw at any time and without giving a reason and this will not affect the standard of care you receive now or in the future. We would not collect any new information on you. However, any information that we had already collected would be kept by the study team.

**What will happen to me if I take part?**

If you decide to take part then a research worker will arrange to meet with you at a time that is convenient for you. At the appointment the research worker will explain the study to you in more detail, check you are eligible for the study and answer any questions that


For peer review only - http://bmjopen.bmj.com/site/about/guidelines.xhtml

you may have. We will give you another copy of this Information Sheet to keep and ask you to sign a consent form.

The research worker will then collect some simple information on things such as your age, previous medical history, current medications and employment history. They will undertake an assessment of any psychological problems that you may have and ask you to complete a number of questionnaires. In total this will take about 1.5 hours.  and will explain how treatment might help you. They will also carefully check that it is safe to give you TMS treatment, such as whether you have seizures (specifically epileptic seizures).

You will then be randomly assigned to either Group A, where you will receive the active treatment, or to Group B where you will receive the inactive treatment. The randomisation will be done by someone who does not know you and who is not directly involved in the study.

You will then be invited for the first treatments session. The treatment itself will take about 30 minutes and beforehand your strength will be tested by a member of the research team and you will be asked to fill in some more questionnaires about your current symptoms and health which will take approximately another 60 minutes so the whole session will take about an hour and a half.  You will then be invited back for another identical treatment session 1 month later. Another 2 months later, so 3 months after the first session, you will be invited for a final session – this time with no treatment but just the examination and questionnaires. All these sessions will be arranged at a time to suit you and we will provide your transport costs.

**How long will I be in the study?**

If you agree to take part in this part of the study it will take 3 months from the start of treatment until the completion of the last follow up session.

**What are the possible risks of taking part?**

There are some risks to taking part in the study as TMS can cause side effects. The most common side effect is that some people can find the TMS treatment uncomfortable around the area it is delivered to (the scalp) and for some this experience is painful but the vast majority of people given the type of TMS in this study find it tolerable.

It can also cause headaches which generally resolve soon after the treatment is given. Very rarely it can cause seizures – but is only reported to occur with higher 'doses' of TMS than used in this study and only in those with, or predisposed to, epilepsy - which is why this is carefully screened for beforehand.

It is also possible that some of the questionnaires you will be asked to fill might cause you distress to answer as they ask about you past psychiatric history and if you have suffered from any abuse. If you experience any of these issues you can discuss them with a member of the research team or your GP and re-evaluate whether you want to continue with the study or not.

**What are the possible benefits of taking part?**

By taking part in the study you will help us understand more about treatments that are effective in helping people with weakness caused by CD.  We cannot be sure at this stage whether the active TMS will be any more effective than the inactive TMS and


For peer review only - http://bmjopen.bmj.com/site/about/guidelines.xhtml
For peer review only

therefore whether you will personally benefit, regardless of which group you are allocated to.

**Will taking part or not influence my medical care?**

Your participation will have no influence on your medical care. There will be no restrictions on your diet or lifestyle during the study. Any doctors or other healthcare professionals you see can make any changes to your medication or other treatments that they feel are necessary for you. Similarly, as mentioned above, not taking part will have no influence on any aspect of your care.

**What expenses will be covered?**

Whichever group you are allocated to, we will pay for your travel up to a maximum £25 for each assessment that is necessary. However, if you take time off work to attend the study appointments we cannot pay you or your employer for this.

**Will my taking part in this study be kept confidential?**

All information which is collected about you during the course of the research will be kept strictly confidential. The research workers who contact you will need to keep your contact details at the university research sites, but only for the purposes of contacting you about arranging to see you or to send you questionnaires. Any other information about you will have your name and address removed so that you cannot be recognised from it. We will not identify you in our computers or publications by name, and will only refer to you by participant number, which will be used in place of your name on any future publications. All information will be stored on password protected computers and paperwork will be stored securely in locked university offices.

If you take part in this Randomised Controlled Trial (RCT) we will ask if we can contact you, perhaps through your GP, if you move house during our study. With your permission we would want to inform your GP that you are taking part in the study and potentially also see your medical file. We would also need to inform your GP or other professionals if one of the health professionals or research workers in the study became concerned about your well-being or about the implications of what you tell us for someone else's well-being. We would of course discuss this with you if such a situation arose.

**What will happen if new information becomes available?**

Sometimes during the course of a study new information might become available about the treatment that is being tested. If this happens, either your medical doctor or a member of the research team will contact you and arrange to talk to you about this and discuss with you whether you want to continue. If you decide to withdraw from the study your doctor will make arrangements for your care to continue. If you decide to continue in the study you may be asked to sign an updated consent form.

**What happens when the trial is over?**

Once the trial is over, we will see whether the active TMS has helped people reduce their weakness any more than the inactive TMS. If you did not receive active TMS during the study then the doctors treating you will decide whether you might still benefit from this and if so they will refer you for this treatment.

**What happens if something goes wrong?**


For peer review only - http://bmjopen.bmj.com/site/about/guidelines.xhtml

We do not expect there to be any significant adverse effects from taking part in this study. However if you are harmed during the study and this is due to someone’s negligence, then you may have grounds for legal action for compensation against the NHS but you may have to pay your legal costs. Regardless of this, if you wish to complain, or have any concerns about any aspect of the way you have been approached or treated during the course of this study, the normal National Health Service complaints mechanisms should be available to you.

King’s College London holds insurance policies that apply to this study. If you experience harm or injury as a result of taking part in this study you may be eligible to claim compensation without having to prove that King’s College London is at fault. This does not affect your legal rights to seek compensation.

If for any reason your symptoms get much worse during the study, then you will be able to talk to your medical specialist or any of us who are involved in the study and discuss what you want to do.

**What happens to the results of the research study?**

We will publish the results of the research in scientific journals and we will present the results at scientific meetings. In addition we will talk to service providers about the results of our research. We will not identify you in any report/publication. If you would like a copy of the published results, we can provide this at the end of the study.

**How often will I be contacted by the investigators?**

We will need to contact you at different stages of the study to arrange treatment or follow up sessions and will give you two reminders to let us have this information. If at any particular stage you change your mind about taking part in the study and we do not hear from you at all, we will contact you on only one further occasion to discuss the study. If we cannot discuss this with you we will assume you have chosen to leave the study. We can reassure you that you will not be contacted repeatedly if you decide you no longer wish to be part of the study. If you then change your mind about letting us have the information we asked for, you can contact us by phone, letter or email to then re-join the study if you wish.

**Can my participation in the study be discontinued by the investigators?**

Yes. At any time during the study, the investigators have the right to end your participation in the study for any reason. If so, this reason will be explained to you. If later on in the study it is concluded that you no longer have capacity to consent to participating we would like to be able to continue to use any data that we have already collected, in an anonymised form.

**Who is organized, funded and reviewed the research?**

The research is funded by the National Institute of Health Research, and administered by the Institute of Psychiatry Psychology & Neuroscience, part of King’s College London. The study has been reviewed and approved by a UK Research Ethics Committee (London-Stanmore Research Ethics Committee - study reference number 17/LO/0410).


For peer review only - http://bmjopen.bmj.com/site/about/guidelines.xhtml

**Contacts for Further Information**

If you require any further information, please contact Dr Nicholson or a member of the research team at the Section of Cognitive Neuropsychiatry (PO68), Institute of Psychiatry Psychology & Neuroscience, De Crespigny Park, London SE5 8AF
Tel: 0207 848 5136 Fax: 0207 848 0572 Email timothy.nicholson@kcl.ac.uk.

You will be given a copy of this information sheet and a signed consent form to keep.

---

If you would like any independent advice about taking part in a research study, or have concerns about the conduct of the study, please contact your Trust Patient Advice and Liaison Service (PALS). PALS offers free confidential advice, support and information on health-related matters and are independent of clinical services. They provide a point of contact for patients, their families and their carers. PALS also helps to improve the NHS by listening to your concerns and suggestions. You can find your nearest PALS on the NHS Choices website: http://www.nhs.uk/Service-Search/Patient-advice-and-liaison-services-(PALS)/LocationSearch/363

Local PALS offices are also listed below:

**South London & Maudsley NHS Foundation Trust (SLAM) PALS**
Website: http://www.slam.nhs.uk/patients-and-carers/advice-and-information
Email: pals@slam.nhs.uk
Phone: 0800 731 2864 (freephone number)

**King's College Hospital NHS Foundation Trust (KCH) PALS**
Online contact form: https://www.kch.nhs.uk/contact/pals
Email: kch-tr.PALS@nhs.net
Phone: 020 3299 3601, 9am to 4.30pm, Monday to Friday (not bank holidays)

**Guys and St Thomas' NHS Foundation Trust (GST) PALS**
Online contact form: http://www.guysandstthomas.nhs.uk/contact-us/feedback-forms/Questions-about-care.aspx
Email: pals@gstt.nhs.uk
Phone: 020 7188 8801


For peer review only - http://bmjopen.bmj.com/site/about/guidelines.xhtml

## Supplementary File 2

*Table 2.1. Concomitant treatments by group and timepoint**

| Treatment | Baseline | TMS Visit 1 n (%) | TMS Visit 2 n (%) | Follow up n (%) |
|---|---|---|---|---|
| **Medication** | Active=10 (100)<br>Inactive=10 (100) | Active=10 (100)<br>Inactive=9 (100) | Active=6 (100)<br>Inactive=8 (100) | Active=9 (100)<br>Inactive=9 (100) |
| **Physiotherapy** | Active=4 (40)<br>Inactive=2 (20) | Active=4 (40)<br>Inactive=2 (2) | Active=0 (0)<br>Inactive=1 (13) | Active=1 (11)<br>Inactive=1 (11) |
| **Neurology inpatient** | Active=0 (0)<br>Inactive=0 (0) | Active=1 (10)<br>Inactive=0 (0) | Active=0 (0)<br>Inactive=0 (0) | Active=0 (0)<br>Inactive=0 (0) |
| **General inpatient** | Active=3 (30)<br>Inactive=1 (10) | Active=2 (20)<br>Inactive=1 (11) | Active=0 (0)<br>Inactive=1 (13) | Active=0 (0)<br>Inactive=0 (0) |
| **Specialist MDT inpatient neurorehabilitation** | Active=1 (10)<br>Inactive=1 (10) | Active=1 (10)<br>Inactive=1 (11) | Active=1 (17)<br>Inactive=1 (13) | Active=1 (11)<br>Inactive=1 (11) |
| **Specialist MDT day hospital** | Active=0 (0)<br>Inactive=1 (10) | Active=0 (0)<br>Inactive=1 (11) | Active=0 (0)<br>Inactive=0 (0) | Active=1 (11)<br>Inactive=0 (0) |
| **CBT / Psychology** | Active=2 (20)<br>Inactive=1 (10) | Active=2 (20)<br>Inactive=1 (11) | Active=1 (17)<br>Inactive=0 (0) | Active=1 (11)<br>Inactive=2 (22) |
| **Occupational therapy** | Active=2 (20)<br>Inactive=1 (10) | Active=2 (20)<br>Inactive=1 (11) | Active=1 (17)<br>Inactive=1 (13) | Active=1 (11)<br>Inactive=0 (0) |
| **Psychiatry (outpatient)** | Active=0 (0)<br>Inactive=0 (0) | Active=0 (0)<br>Inactive=0 (0) | Active=0 (0)<br>Inactive=1 (13) | Active=1 (11)<br>Inactive=1 (11) |

**Key:** CBT=cognitive behavioural therapy; MDT=multidisciplinary team; TMS=transcranial magnetic stimulation

For peer review only

## Supplementary File 3 – Outcome measure completion data

***Table 3.1. Data quality by timepoint****

| Outcome measure | TMS Visit 1 n (%) | TMS Visit 2 n (%) | Follow up n (%) |
|---|---|---|---|
| **CGI Patient** | 21 (100) | 16 (100) | 19 (100) |
| **CGI Outcome assessor** | 21 (100) | 16 (100) | 20 (105) |
| **CGI Carer** | 2 (10) | 4 (25) | 4 (21) |
| **SF36: Physical Function** | 21 (100) | 16 (100) | 19 (100) |
| **SF36: Role Physical** | 20 (95) | 16 (100) | 19 (100) |
| **SF36: Bodily Pain** | 21 (100) | 16 (100) | 19 (100) |
| **SF36: General Health** | 21 (100) | 16 (100) | 19 (100) |
| **SF36: Vitality** | 21 (100) | 16 (100) | 19 (100) |
| **SF36: Social Functioning** | 21 (100) | 16 (100) | 19 (100) |
| **SF36: Role Emotional** | 18 (86) | 16 (100) | 19 (100) |
| **SF36: Mental Health** | 21 (100) | 16 (100) | 19 (100) |
| **Barthel Index** | 21 (100) | 16 (100) | 20 (105) |
| **FIM-FAM** | 4 (19) | 2 (12) | 2 (11) |
| **GAD 7** | 21 (100) | 16 (100) | 19 (100) |
| **PHQ 9** | 21 (100) | 16 (100) | 19 (100) |
| **PHQ 15** | 21 (100) | 16 (100) | 19 (100) |
| **CORE-10** | 21 (100) | 16 (100) | 19 (100) |
| **WSAS** | 21 (100) | 16 (100) | 19 (100) |
| **Left Arm; Strength** | 20 (95) | 15 (94) | 17 (89) |
| **Left Arm: Weakness** | 20 (95) | 15 (94) | 18 (95) |
| **Right Arm: Strength** | 20 (95) | 15 (94) | 17 (89) |
| **Right Arm: Weakness** | 20 (95) | 15 (94) | 18 (95) |
| **Left Leg; Strength** | 21 (100) | 16 (100) | 18 (95) |
| **Left Leg: Weakness** | 21 (100) | 16 (100) | 19 (100) |
| **Right Leg: Strength** | 20 (95) | 15 (94) | 17 (89) |
| **Right Leg: Weakness** | 20 (95) | 15 (94) | 18 (95) |
| **Dynamometry Left Arm: Max** | 20 (95) | 15 (94) | 17 (89) |
| **Dynamometry Left Arm: Max** | 20 (95) | 15 (94) | 18 (95) |
| **Dynamometry Left Arm: Max** | 20 (95) | 15 (94) | 17 (89) |
| **Dynamometry Left Arm: Max** | 20 (95) | 15 (94) | 18 (95) |

**Key:** CGI=Clinical Global Impression; CORE=10=Clinical Outcomes in Routine Evaluation-10 item; GAD-7=Generalised Anxiety Disorder-7 item; KG=kilogram; PHQ=Patient Health Questionnaire; SF-36=Short Form Health Survey-36 item; TMS=transcranial magnetic stimulation; WSAS=Work & Social Adjustment Scale

******Percentages calculated relative to the number of patients in attendance in each group*

For peer review only

## Supplementary File 4 - Descriptive statistics and effect sizes for primary and secondary outcomes

### *Supplementary Table 4.1. Patient CGI-I ratings*

| | | Visit 1 | | | | Visit 2 | | | | Follow-up | |
|---|---|---|---|---|---|---|---|---|---|---|---|
| | | Pre-TMS | | Post-TMS | | Pre-TMS | | Post-TMS | | | |
| | | **Active** | **Inactive** | **Active** | **Inactive** | **Active** | **Inactive** | **Active** | **Inactive** | **Active** | **Inactive** |
| **Very much improved** | **n (%)** | 0 (0) | 0 (0) | 0 (0) | 0 (0) | 0 (0) | 1 (10) | 0 (0) | 0 (0) | 0 (0) | 0 (0) |
| **Much improved** | **n (%)** | 0 (0) | 1 (9) | 0 (0) | 1 (9) | 4 (67) | 1 (10) | 4 (67) | 2 (20) | 4 (44) | 2 (20) |
| **Minimally improved** | **n (%)** | 1 (10) | 2 (18) | 1 (10) | 2 (18) | 0 (0) | 1 (10) | 0 (0) | 1 (10) | 1 (11) | 4 (40) |
| **No change** | **n (%)** | 3 (30) | 5 (45) | 4 (40) | 6 (55) | 0 (0) | 2 (20) | 0 (0) | 2 (20) | 2 (22) | 1 (10) |
| **Minimally worse** | **n (%)** | 3 (30) | 1 (9) | 2 (20) | 0 (0) | 1 (17) | 3 (30) | 1 (17) | 2 (20) | 1 (11) | 2 (20) |
| **Much worse** | **n (%)** | 3 (30) | 2 (18) | 3 (30) | 2 (18) | 1 (17) | 2 (20) | 1 (17) | 3 (30) | 0 (0) | 1 (10) |
| **Very much worse** | **n (%)** | 0 (0) | 0 (0) | 0 (0) | 0 (0) | 0 (0) | 0 (0) | 0 (0) | 0 (0) | 1 (11) | 0 (0) |
| **Total** | **n (%)** | 10 (100) | 11 (100) | 10(100) | 11 (100) | 6 (60) | 10 (91) | 6 (60) | 10 (91) | 9 (90) | 10 (91) |
| **Missing*** | **n (%)** | 0 (0) | 0 (0) | 0 (0) | 0 (0) | 4 (40) | 1 (9) | 4 (40) | 1 (9) | 1 (10) | 1 (9) |
| **Effect size (negative = benefit)** | **Cliff's delta (95% CI)** | 0.35 (-0.17, 0.71) | | 0.35 (-0.15, 0.7) | | -0.35 (-0.73, 0.19) | | -0.44 (-0.79, 0.13) | | -0.2 (-0.6, 0.28) | |

**Key:** CGI-I=Clinical Global Impression-Improvement; CI=confidence interval; TMS=transcranial magnetic stimulation

*****
*Percentage calculated relative to total number of participants enrolled in study*

***Supplementary Table 4.2. Outcome assessor CGI-I ratings***

| | | Visit 1 | | | | Visit 2 | | | | Follow-up | |
|---|---|---|---|---|---|---|---|---|---|---|---|
| | | Pre-TMS | | Post-TMS | | Pre-TMS | | Post-TMS | | | |
| | | Active | Inactive | Active | Inactive | Active | Inactive | Active | Inactive | Active | Inactive |
| **Very much improved** | **n (%)** | 0 (0) | 0 (0) | 0 (0) | 0 (0) | 0 (0) | 0 (0) | 0 (0) | 0 (0) | 0 (0) | 0 (0) |
| **Much improved** | **n (%)** | 0 (0) | 0 (0) | 0 (0) | 0 (0) | 3 (50) | 2 (20) | 3 (50) | 2 (20) | 4 (44) | 2 (18) |
| **Minimally improved** | **n (%)** | 1 (10) | 3 (27) | 1 (10) | 3 (27) | 1 (17) | 1 (10) | 1 (17) | 1 (10) | 3 (33) | 5 (45) |
| **No change** | **n (%)** | 3 (30) | 7 (64) | 4 (40) | 6 (55) | 0 (0) | 5 (50) | 0 (0) | 5 (50) | 1 (11) | 2 (18) |
| **Minimally worse** | **n (%)** | 3 (30) | 1 (9) | 1 (10) | 2 (18) | 2 (33) | 2 (20) | 2 (33) | 2 (20) | 0 (0) | 1 (9) |
| **Much worse** | **n (%)** | 3 (30) | 0 (0) | 4 (40) | 0 (0) | 0 (0) | 0 (0) | 0 (0) | 0 (0) | 0 (0) | 1 (9) |
| **Very much worse** | **n (%)** | 0 (0) | 0 (0) | 0 (0) | 0 (0) | 0 (0) | 0 (0) | 0 (0) | 0 (0) | 1 (11) | 0 (0) |
| **Total** | **n (%)** | 10 (100) | 11 (100) | 10 (100) | 11 (100) | 6 (60) | 10 (91) | 6 (60) | 10 (91) | 9 (90) | 11 (100) |
| **Missing*** | **n (%)** | 0 (0) | 0 (0) | 0 (0) | 0 (0) | 4 (40) | 1 (9) | 4 (40) | 1 (9) | 1 (10) | 0 (0) |
| **Effect size (negative = benefit)** | **Cliff's delta (95% CI)** | 0.55 (0.05, 0.83) | | 0.45 (-0.06, 0.77) | | -0.29 (-0.69, 0.25) | | -0.29 (-0.69, 0.25) | | -0.26 (-0.65, 0.23) | |

**Key:** CGI-I=Clinical Global Impression-Improvement; CI=confidence interval; TMS=transcranial magnetic stimulation

**Percentage calculated relative to total number of participants enrolled in the study*

For peer review only

***Supplementary Table 4.3. Patient weakness ratings***

| | | Visit 1 | | | | Visit 2 | | | | Follow-up | |
|---|---|---|---|---|---|---|---|---|---|---|---|
| | | Pre-TMS | | Post-TMS | | Pre-TMS | | Post-TMS | | | |
| | | Active | Inactive | Active | Inactive | Active | Inactive | Active | Inactive | Active | Inactive |
| **No weakness** | **n(%)** | 2 (20) | 1 (9) | 3 (30) | 1 (9) | 0 (0) | 1 (10) | 1 (17) | 1 (10) | 2 (22) | 0 (0) |
| **Mild weakness** | **n(%)** | 1 (10) | 3 (27) | 0 (0) | 5 (45) | 1 (17) | 4 (40) | 0 (0) | 5 (50) | 1 (11) | 5 (50) |
| **Moderate weakness** | **n(%)** | 1 (10) | 3 (27) | 1 (10) | 0 (0) | 0 (0) | 1 (10) | 1 (17) | 0 (0) | 3 (33) | 1 (10) |
| **Severe weakness** | **n(%)** | 3 (30) | 1 (9) | 3 (30) | 2 (18) | 3 (50) | 2 (20) | 3 (50) | 3 (30) | 1 (11) | 3 (30) |
| **Very severe weakness** | **n(%)** | 3 (30) | 3 (27) | 3 (30) | 3 (27) | 2 (33) | 2 (20) | 1 (17) | 1 (10) | 2 (22) | 1 (10) |
| **Total** | **n(%)** | 10 (100) | 11 (100) | 10 (100) | 11 (100) | 6 (60) | 10 (91) | 6 (60) | 10 (91) | 9 (90) | 10 (91) |
| **Missing*** | **n(%)** | 0 (0) | 0 (0) | 0 (0) | 0 (0) | 4 (40) | 1 (9) | 4 (40) | 1 (9) | 1 (10) | 1 (9) |
| **Effect size (negative = treatment benefit)** | **Cliff's Delta (95% CI)** | 0.09 (-0.41, 0.55) | | 0.04 (-0.46, 0.51) | | 0.27 (-0.11, 0.58) | | 0.17 (-0.25, 0.53) | | -0.08 (-0.51, 0.37) | |

**Key:** CI=confidence interval; TMS=transcranial magnetic stimulation

**Percentage calculated relative to total number of participants enrolled in the study*

For peer review only

*Supplementary Table 4.4. Additional secondary outcome measures*

| | | Visit 1 | | Visit 2 | | Follow-up |
|---|---|---|---|---|---|---|
| **Measure** | **Statistic** | **Pre-TMS** | **Post-TMS** | **Pre-TMS** | **Post-TMS** | |
| **Target limb strength rating (0-100%)** | **Mean (SD)** | Active=42.5 (37.4)<br>Inactive=52.3 (30.4) | Active=44.5 (40.6)<br>Inactive=52.7 (35) | Active=38.3 (25)<br>Inactive=55 (34) | Active=42.8 (34.1)<br>Inactive=57 (34) | Active=41.9 (27.5)<br>Inactive=51.8 (36.2) |
| | **Cohen's d** | 0.29 (-0.63, 1.21) | 0.22 (-0.7, 1.14) | 0.54 (-0.59, 1.66) | 0.42 (-0.7, 1.53) | 0.3 (-0.71, 1.31) |
| **Dynamometry – left arm (average KG)** | **Mean (SD)** | Active=12.4 (10.8)<br>Inactive=6.1 (6.9) | Active=11.3 (11.7)<br>Inactive=7 (8.9) | Active=11.9 (3.7)<br>Inactive=6.3 (11) | Active=11.6 (6.1)<br>Inactive=6.4 (12) | Active=10.7 (9.1)<br>Inactive=9.7 (12.3) |
| | **Cohen's d** | 0.68 (-0.35, 1.71) | 0.41 (-0.61, 1.42) | 0.65 (-0.6, 1.91) | 0.53 (-0.72, 1.77) | 0.09 (-1.02, 1.21) |
| **Dynamometry – right arm (average KG)** | **Mean (SD)** | Active=9.4 (9)<br>Inactive=10.5 (9.1) | Active=9.4 (8.6)<br>Inactive=9.6 (8.8) | Active=11.9 (6.6)<br>Inactive=10.3 (9.1) | Active=11.9 (9)<br>Inactive=9.6 (12.2) | Active=12.5 (12.9)<br>Inactive=11.1 (9.1) |
| | **Cohen's d** | -0.12 (-1.09, 0.85) | -0.02 (-0.99, 0.95) | 0.19 (-0.99, 1.37) | 0.21 (-0.97, 1.39) | 0.13 (-0.95, 1.2) |
| **PHQ-15** | **Mean (SD)** | Active=15.4 (3.3)<br>Inactive=13.5 (6) | | Active=15.7 (4.4)<br>Inactive=14.2 (7.2) | | Active=15.2 (5.3)<br>Inactive=12.4 (6) |
| | **Cohen's d** | -0.39 (-1.31, 0.54) | | -0.26 (-1.38, 0.85) | | -0.5 (-1.48, 0.49) |
| **PHQ-9** | **Mean (SD)** | Active=15 (5.2)<br>Inactive=14.1 (8.9) | | Active=13.3 (2.2)<br>Inactive=12.8 (8.4) | | Active=14.3 (6.1)<br>Inactive=12.3 (11.2) |
| | **Cohen's d** | -0.13 (-1.04, 0.79) | | -0.1 (-1.21, 1.01) | | -0.22 (-1.19, 0.75) |
| **GAD-7** | **Mean (SD)** | Active=8.7 (5.6)<br>Inactive=10.5 (7.7) | | Active=7.3 (3.4)<br>Inactive=7.5 (7) | | Active=7.1 (4.9)<br>Inactive=9.1 (7.6) |
| | **Cohen's d** | 0.28 (-0.64, 1.2) | | 0.03 (-1.07, 1.14) | | 0.32 (-0.66, 1.29) |

For peer review only

| | | Visit 1 | | Visit 2 | | Follow-up |
|---|---|---|---|---|---|---|
| **Measure** | **Statistic** | **Pre-TMS** | **Post-TMS** | **Pre-TMS** | **Post-TMS** | |
| **CORE-10** | **Mean (SD)** | Active=18.4 (8.3)<br>Inactive=17.1 (10.3) | | Active=16.7 (4)<br>Inactive=16.5 (9.4) | | Active=14.8 (5.2)<br>Inactive=16.4 (8.2) |
| | **Cohen's d** | -0.14 (-1.06, 0.77) | | -0.03 (-1.13, 1.08) | | 0.24 (-0.73, 1.21) |
| **SF-36 Physical functioning** | **Mean (SD)** | Active=10 (11.5)<br>Inactive=22.7 (22.2) | | Active=15.8 (21.3)<br>Inactive=30 (28.9) | | Active=21.2 (26.4)<br>Inactive=28 (29.6) |
| | **Cohen's d** | 0.73 (-0.21, 1.68) | | 0.58 (-0.55, 1.71) | | 0.24 (-0.73, 1.22) |
| **SF-36 Physical role** | **Mean (SD)** | Active=2.5 (7.9)<br>Inactive=15 (33.7) | | Active=4.2 (10.2)<br>Inactive=20 (36.9) | | Active=8.3 (25)<br>Inactive=17.5 (37.4) |
| | **Cohen's d** | 0.51 (-0.44, 1.46) | | 0.67 (-0.46, 1.81) | | 0.29 (-0.68, 1.27) |
| **SF-36 Bodily pain** | **Mean (SD)** | Active=22.2 (18.3)<br>Inactive=25 (27.1) | | Active=29.8 (27.7)<br>Inactive=19.1 (22.1) | | Active=31 (23.6)<br>Inactive=32.6 (21) |
| | **Cohen's d** | 0.12 (-0.79, 1.04) | | -0.42 (-1.53, 0.7) | | 0.07 (-0.9, 1.04) |
| **SF-36 General health** | **Mean (SD)** | Active=29.9 (9.7)<br>Inactive=30.8 (21.2) | | Active=38.2 (15.8)<br>Inactive=35.4 (26.2) | | Active=31.6 (11)<br>Inactive=39.8 (20.2) |
| | **Cohen's d** | 0.06 (-0.86, 0.97) | | -0.14 (-1.25, 0.97) | | 0.51 (-0.47, 1.5) |
| **SF-36 Vitality** | **Mean (SD)** | Active=17.5 (11.6)<br>Inactive=22.9 (24.6) | | Active=20 (8.4)<br>Inactive=26.5 (25.6) | | Active=29.4 (12.6)<br>Inactive=30.5 (30.1) |
| | **Cohen's d** | 0.28 (-0.63, 1.2) | | 0.39 (-0.73, 1.51) | | 0.05 (-0.92, 1.02) |
| **SF-36 Social functioning** | **Mean (SD)** | Active=20 (17.9)<br>Inactive=28.4 (29.1) | | Active=39.6 (31)<br>Inactive=42.5 (35) | | Active=20.8 (25.8)<br>Inactive=40 (33.7) |
| | **Cohen's d** | 0.35 (-0.57, 1.27) | | 0.09 (-1.02, 1.2) | | 0.64 (-0.35, 1.64) |

For peer review only

| | | Visit 1 | | Visit 2 | | Follow-up |
|---|---|---|---|---|---|---|
| Measure | Statistic | Pre-TMS | Post-TMS | Pre-TMS | Post-TMS | |
| **SF-36 Emotional role** | **Mean (SD)** | Active=12.5 (24.8)<br>Inactive=46.7 (50.2) | | Active=25 (41.8)<br>Inactive=33.3 (41.6) | | Active=59.3 (40.1)<br>Inactive=30 (48.3) |
| | **Cohen's d** | 0.9 (-0.16, 1.95) | | 0.2 (-0.91, 1.31) | | -0.66 (-1.66, 0.33) |
| **SF-36 Mental health** | **Mean (SD)** | Active=54.4 (20.8)<br>Inactive=54.5 (30) | | Active=56 (14.8)<br>Inactive=56.8 (29.7) | | Active=59.6 (18.4)<br>Inactive=59.6 (25.6) |
| | **Cohen's d** | 0.01 (-0.91, 0.92) | | 0.03 (-1.08, 1.14) | | 0 (-0.97, 0.97) |
| **Barthel** | **Mean (SD)** | Active=12.3 (3.8)<br>Inactive=14.5 (5.6) | | Active=12.5 (4.4)<br>Inactive=14.4 (5.6) | | Active=14.9 (4.2)<br>Inactive=15.8 (5.2) |
| | **Cohen's d** | 0.44 (-0.48, 1.37) | | 0.36 (-0.75, 1.48) | | 0.19 (-0.75, 1.14) |
| **WSAS** | **Mean (SD)** | Active=32.3 (3.4)<br>Inactive=29.1 (9.1) | | Active=29.7 (8.3)<br>Inactive=23.9 (10.6) | | Active=29.9 (9.9)<br>Inactive=23.2 (11.8) |
| | **Cohen's d** | -0.48 (-1.4, 0.45) | | -0.63 (-1.76, 0.5) | | -0.62 (-1.61, 0.37) |

**Key:** CORE=10=Clinical Outcomes in Routine Evaluation-10 item; GAD-7=Generalised Anxiety Disorder-7 item; IQR=interquartile range; KG=kilogram; PHQ=Patient Health Questionnaire; SD=standard deviation; SF-36=Short Form Health Survey-36 item; TMS=transcranial magnetic stimulation; WSAS=Work & Social Adjustment Scale

For peer review only

# CONSORT 2010 checklist of information to include when reporting a randomised trial*

| Section/Topic | Item No | Checklist item | Reported on page No |
|---|---|---|---|
| **Title and abstract** | | | |
| | 1a | Identification as a randomised trial in the title | 1 |
| | 1b | Structured summary of trial design, methods, results, and conclusions (for specific guidance see CONSORT for abstracts) | 2 |
| **Introduction** | | | |
| Background and objectives | 2a | Scientific background and explanation of rationale | 4-5 |
| | 2b | Specific objectives or hypotheses | 5 |
| **Methods** | | | |
| Trial design | 3a | Description of trial design (such as parallel, factorial) including allocation ratio | 6 |
| | 3b | Important changes to methods after trial commencement (such as eligibility criteria), with reasons | 11 |
| Participants | 4a | Eligibility criteria for participants | 6-7 |
| | 4b | Settings and locations where the data were collected | 6 |
| Interventions | 5 | The interventions for each group with sufficient details to allow replication, including how and when they were actually administered | 8-11 |
| Outcomes | 6a | Completely defined pre-specified primary and secondary outcome measures, including how and when they were assessed | 11-12 |
| | 6b | Any changes to trial outcomes after the trial commenced, with reasons | NA |
| Sample size | 7a | How sample size was determined | 13-14 |
| | 7b | When applicable, explanation of any interim analyses and stopping guidelines | NA |
| Randomisation: | | | |
| Sequence generation | 8a | Method used to generate the random allocation sequence | 12 |
| | 8b | Type of randomisation; details of any restriction (such as blocking and block size) | 12 |
| Allocation concealment mechanism | 9 | Mechanism used to implement the random allocation sequence (such as sequentially numbered containers), describing any steps taken to conceal the sequence until interventions were assigned | 12 |
| Implementation | 10 | Who generated the random allocation sequence, who enrolled participants, and who assigned participants to interventions | |
| Blinding | 11a | If done, who was blinded after assignment to interventions (for example, participants, care providers, those | 12-13 |

For peer review only

| | | | |
|---|---|---|---|
| | | assessing outcomes) and how | |
| | 11b | If relevant, description of the similarity of interventions | 8-11 |
| Statistical methods | 12a | Statistical methods used to compare groups for primary and secondary outcomes | 14-15 |
| | 12b | Methods for additional analyses, such as subgroup analyses and adjusted analyses | NA |
| **Results** | | | |
| Participant flow (a diagram is strongly recommended) | 13a | For each group, the numbers of participants who were randomly assigned, received intended treatment, and were analysed for the primary outcome | 18-19 (Fig 1) |
| | 13b | For each group, losses and exclusions after randomisation, together with reasons | 18-19 (Fig 1) |
| Recruitment | 14a | Dates defining the periods of recruitment and follow-up | 6, 19 |
| | 14b | Why the trial ended or was stopped | 18 |
| Baseline data | 15 | A table showing baseline demographic and clinical characteristics for each group | 17-18 |
| Numbers analysed | 16 | For each group, number of participants (denominator) included in each analysis and whether the analysis was by original assigned groups | 18-19 (Fig 1) |
| Outcomes and estimation | 17a | For each primary and secondary outcome, results for each group, and the estimated effect size and its precision (such as 95% confidence interval) | 21-22 (Fig 2) (Supplementary File 4) |
| | 17b | For binary outcomes, presentation of both absolute and relative effect sizes is recommended | NA |
| Ancillary analyses | 18 | Results of any other analyses performed, including subgroup analyses and adjusted analyses, distinguishing pre-specified from exploratory | NA |
| Harms | 19 | All important harms or unintended effects in each group (for specific guidance see CONSORT for harms) | 20 |
| **Discussion** | | | |
| Limitations | 20 | Trial limitations, addressing sources of potential bias, imprecision, and, if relevant, multiplicity of analyses | 26-27 |
| Generalisability | 21 | Generalisability (external validity, applicability) of the trial findings | 26-27 |
| Interpretation | 22 | Interpretation consistent with results, balancing benefits and harms, and considering other relevant evidence | 22-26 |
| **Other information** | | | |
| Registration | 23 | Registration number and name of trial registry | 3 |
| Protocol | 24 | Where the full trial protocol can be accessed, if available | NA |
| Funding | 25 | Sources of funding and other support (such as supply of drugs), role of funders | 28 |

*We strongly recommend reading this statement in conjunction with the CONSORT 2010 Explanation and Elaboration for important clarifications on all the items. If relevant, we also recommend reading CONSORT extensions for cluster randomised trials, non-inferiority and equivalence trials, non-pharmacological treatments, herbal interventions, and pragmatic trials. Additional extensions are forthcoming: for those and for up to date references relevant to this checklist, see www.consort-statement.org.


For peer review only - http://bmjopen.bmj.com/site/about/guidelines.xhtml
For peer review only

BMJ Open

# Trial Of Neurostimulation In Conversion Symptoms (‘TONICS’): a feasibility randomised controlled trial of transcranial magnetic stimulation for functional limb weakness

| | |
|---|---|
| Journal: | *BMJ Open* |
| Manuscript ID | bmjopen-2020-037198.R2 |
| Article Type: | Original research |
| Date Submitted by the Author: | 09-Jul-2020 |
| Complete List of Authors: | Pick, Susannah; Institute of Psychiatry Psychology and Neuroscience<br>Hodsoll, John ; Institute of Psychiatry Psychology and Neuroscience<br>Stanton, Biba; Institute of Psychiatry Psychology and Neuroscience; King's College Hospital NHS Foundation Trust<br>Eskander, Amy; King's College Hospital NHS Foundation Trust<br>Stavropoulos, Ioannis; King's College Hospital NHS Foundation Trust<br>Samra, Kiran; King's College Hospital NHS Foundation Trust<br>Bottini, Julia; King's College Hospital NHS Foundation Trust<br>Ahmad, Hena; King's College Hospital NHS Foundation Trust<br>David, Anthony; University College London, Institute of Mental Health<br>Purves, Alistair; King's College Hospital NHS Foundation Trust<br>Nicholson, Timothy; Institute of Psychiatry Psychology and Neuroscience |
| <b>Primary Subject Heading</b>: | Neurology |
| Secondary Subject Heading: | Mental health |
| Keywords: | Adult neurology < NEUROLOGY, Adult psychiatry < PSYCHIATRY, Clinical trials < THERAPEUTICS |
| | |

SCHOLARONE™
Manuscripts

*I, the Submitting Author has the right to grant and does grant on behalf of all authors of the Work (as defined in the below author licence), an exclusive licence and/or a non-exclusive licence for contributions from authors who are: i) UK Crown employees; ii) where BMJ has agreed a CC-BY licence shall apply, and/or iii) in accordance with the terms applicable for US Federal Government officers or employees acting as part of their official duties; on a worldwide, perpetual, irrevocable, royalty-free basis to BMJ Publishing Group Ltd ("BMJ") its licensees and where the relevant Journal is co-owned by BMJ to the co-owners of the Journal, to publish the Work in this journal and any other BMJ products and to exploit all rights, as set out in our licence.*

*The Submitting Author accepts and understands that any supply made under these terms is made by BMJ to the Submitting Author unless you are acting as an employee on behalf of your employer or a postgraduate student of an affiliated institution which is paying any applicable article publishing charge ("APC") for Open Access articles. Where the Submitting Author wishes to make the Work available on an Open Access basis (and intends to pay the relevant APC), the terms of reuse of such Open Access shall be governed by a Creative Commons licence – details of these licences and which Creative Commons licence will apply to this Work are set out in our licence referred to above.*

*Other than as permitted in any relevant BMJ Author's Self Archiving Policies, I confirm this Work has not been accepted for publication elsewhere, is not being considered for publication elsewhere and does not duplicate material already published. I confirm all authors consent to publication of this Work and authorise the granting of this licence.*

For peer review only

# Trial Of Neurostimulation In Conversion Symptoms ('TONICS'): a feasibility randomised controlled trial of transcranial magnetic stimulation for functional limb weakness

**Susannah Pick[1], John Hodsoll[1], Biba Stanton[1,2], Amy Eskander[2], Ioannis Stavropoulos[2], Kiran Samra[2], Julia Bottini[2], Hena Ahmad[2], Anthony S. David[3], Alistair Purves[2], & Timothy R. Nicholson[1‡]**

[1]Institute of Psychiatry, Psychology & Neuroscience, King's College London, London, UK
[2] King's College Hospital NHS Foundation Trust, London, UK
[3]Institute of Mental Health, University College London, London, UK

‡corresponding author
Dr Timothy Nicholson. Email: timothy.nicholson@kcl.ac.uk; Tel: 0207 848 5136

For peer review only

Word count: 4996
Tables: 2
Figures: 2
Supplementary files: 4

Keywords: functional neurological disorder, conversion disorder, transcranial magnetic stimulation, functional motor, trial, treatment



For peer review only - http://bmjopen.bmj.com/site/about/guidelines.xhtml

## ABSTRACT

**Objectives:** Transcranial magnetic stimulation (TMS) has been used therapeutically for functional (conversion) motor symptoms but there is limited evidence for its efficacy and the optimal protocol. We examined the feasibility of a novel randomised controlled trial (RCT) protocol of TMS to treat functional limb weakness.

**Design:** A double-blind (patient, outcome assessor) two parallel-arm, controlled RCT.

**Setting:** Specialist neurology and neuropsychiatry services at a large National Health Service Foundation Trust in London, UK.

**Participants:** Patients with DSM-5 diagnosis of functional limb weakness. Exclusion criteria included comorbid neurological or major psychiatric disorder, contraindications to TMS, or previous TMS treatment.

**Interventions:** Patients were randomised to receive either active (single-pulse TMS to primary motor cortex (M1) above resting motor-threshold) or inactive treatment (single-pulse TMS to M1 below resting motor-threshold). Both groups received two TMS sessions, four weeks apart.

**Outcome measures:** We assessed recruitment, randomisation, and retention rates. The primary outcome was patient-rated symptom change (Clinical Global Impression–Improvement scale, CGI-I). Secondary outcomes included clinician-rated symptom change, psychosocial functioning, and disability. Outcomes were assessed at baseline, both TMS visits and at 3-month follow-up.

**Results:** Twenty-two patients were recruited and twenty-one (96%) were successfully randomised (active=10; inactive=11). Nineteen (91%) patients were included at follow-up (active=9; inactive=10). Completion rates for most outcomes were good (80-100%). Most patients were satisfied/very satisfied with the trial in both groups, although ratings were higher in the inactive arm (active=60%, inactive=92%). Adverse events were not more common for the active treatment. Treatment effect sizes for patient-rated CGI-I scores were small-moderate (Cliff's delta= -0.1-0.3, CIs= -0.79-0.28), reflecting a more positive outcome for the active treatment (67% and 44% of active arm rated symptoms as 'much improved' at session 2 and follow-up respectively, versus 20% inactive group). Effect sizes for secondary outcomes were variable.




For peer review only - http://bmjopen.bmj.com/site/about/guidelines.xhtml

For peer review only

**Conclusions:** Our protocol is feasible. The findings suggest that supra-motor threshold TMS of M1 is safe, acceptable and potentially beneficial as a treatment for functional limb weakness. A larger RCT is warranted.

**Trial registration:** ISRCTN51225587

## ARTICLE SUMMARY

### Strengths and limitations of this study

- The study examined the feasibility of a novel, controlled TMS protocol for treating functional limb weakness.
- The TMS protocol has potential to inform the minimal dose required and mechanism of action for positive outcomes in this population.
- Both patients and outcome assessors were blind to treatment allocation, but it was not possible to blind the delivery of the treatment.
- As this was a feasibility study with a small sample size, randomisation might not have adequately balanced group differences across the treatment arms.

For peer review only



For peer review only - http://bmjopen.bmj.com/site/about/guidelines.xhtml

## BACKGROUND

Functional neurological disorder (FND) is defined by neurological symptoms that are incompatible with other medical/neurological diagnoses [1]. FND can resemble any neurological disorder, with seizures, motor (e.g., limb weakness, tremor, dystonia, myoclonus) and sensory (visual, auditory, somatosensory) symptoms predominating. Quality of life and prognosis are often poor [2-4]. Despite recent developments in detection and diagnosis of the disorder [5], there is still a marked paucity of evidence-based, accessible treatment options. There is emerging evidence for the efficacy of some treatment modalities (e.g., specialist physiotherapy for motor symptoms or cognitive behavioural therapy for seizures) [6-9], but availability is currently limited. The development of alternative treatment options that are safe, cost-effective, acceptable to patients and accessible is critical for improving outcomes in this population.

Transcranial magnetic stimulation (TMS) has been explored as a potential treatment option for functional motor symptoms and there is accumulating evidence for its efficacy and safety from uncontrolled studies and five randomised controlled trials (RCTs) [10-15]. These studies used divergent methods and so the optimal protocol is presently unclear, for example, whether to use single pulse (spTMS) or repetitive (rTMS) stimulation; which brain region to target; how many sessions are needed; and the optimal control intervention. Previous studies have generally found post-intervention functional motor symptom improvements following stimulation of primary motor cortex (M1) [11-15]. However, few of these RCTs reported gains in other important outcomes (e.g., comorbid psychological/physical symptoms, quality of life/global functioning, healthcare resource use). Despite post-treatment



For peer review only - http://bmjopen.bmj.com/site/about/guidelines.xhtml

improvements in core FND symptoms following rTMS to M1, Taib et al. [14] for example, did not observe superior improvements in health-related quality of life (SF-36 vitality/general health) for active rTMS relative to sham-TMS, and no improvements were observed in psychological symptoms. Similarly, McWhirter et al. [15] reported improvements in subjective symptoms immediately following spTMS of M1 relative to standard care, but no associated improvements in self-reported mental or physical health (SF-12) or clinician-rated disability (Modified Rankin Scale).

Further research is therefore needed to optimise both TMS treatment and RCT protocols to enable more definitive testing of the efficacy of TMS in improving functional motor symptoms themselves as well as other important outcome domains [16, 17].

## OBJECTIVES

We aimed to explore the feasibility and acceptability of a novel, controlled spTMS protocol for functional limb weakness, to inform the design and implementation of a subsequent larger-scale RCT. The protocol consisted of a minimal 'dose', consisting of two brief sessions of spTMS to M1, with the target region tailored to the specific limb weakness reported by each patient. We compared active stimulation delivered above resting motor threshold (RMT) to a control condition consisting of exactly the same procedures delivered below RMT. We hypothesised that this protocol would be feasible in terms of the following key parameters: recruitment rates, acceptance of randomisation, tolerance of the intervention, successful blinding and completion of outcome measures. We also aimed to estimate the variability of outcome measures and treatment effect sizes to inform design of the next RCT.

For peer review only

## METHODS

### Trial design

The study was a double-blind two parallel arm controlled feasibility RCT of tailored spTMS to M1 in patients with functional limb weakness. The primary outcome was patient-rated symptom change. We also measured a range of other relevant secondary outcome domains to assess their feasibility and acceptability in this population (outlined below).

### Study setting and participants

Ethical approval was received from the London-Stanmore Research Ethics Committee, UK (ref:17/LO/0410). Patients with functional limb weakness were recruited from inpatient and outpatient neurology and neuropsychiatry services across the King's Health Partners (National Health Service, UK), including King's College Hospital, Guy's and St Thomas' Hospital, and the South London and Maudsley NHS Foundation Trusts. Recruitment took place between October 2017 and March 2018.

Inclusion criteria were:

- DSM-5 diagnosis of functional neurological disorder confirmed by a consultant neuropsychiatrist or neurologist
- Motor symptoms defined by functional weakness of at least one limb
- 18 years old or older
- Capacity to consent



For peer review only - http://bmjopen.bmj.com/site/about/guidelines.xhtml

Exclusion criteria were:

- Epilepsy (or considered high risk of epilepsy from medical history)
- Other contraindication to TMS (e.g. cochlear implants, metallic intracranial clips or intracranial surgery in last 12 months)
- Comorbid neurological condition (e.g. multiple sclerosis, stroke)
- Pain as primary symptom
- Previous treatment with TMS (for any condition)
- Non-fluent English speakers (if unable to accurately complete self-report questionnaires)
- Major mental health disorder: current diagnosis of schizophrenia or bipolar disorder; current drug/alcohol dependence
- History of factitious disorder
- Currently involved in another trial

Preliminary eligibility screening was completed by clinical neurology and neuropsychiatry staff. When patients were considered potentially eligible, Participant Information Sheets were provided (Supplementary File 1), and permission sought for the research team (TN/SP) to contact the patient. When permission was granted, a member of the research team subsequently contacted the patient to answer any questions and arrange an initial screening assessment visit, if the patient wished to enrol. Written informed consent was obtained at the initial screening visit, after the study had been explained in full and any remaining questions answered. All participants were told that TMS had shown promising results in previous small-scale research studies and that the current study was aiming to test the treatment more

For peer review only

stringently. Hypotheses regarding the possible mechanisms of treatment were not disclosed. Possible side effects of the treatment were outlined (e.g., headaches, scalp tingling).

Participants were not reimbursed for involvement in the study, but assistance with travel arrangements and expenses was provided, as necessary.

**Patient and Public Involvement**

A specialist service user advisory group was set up to inform the design and conduct of the study. Key national and international patient groups are involved in the dissemination plans.

**Background/screening measures**

At the initial screening visit, demographic details and medical history were obtained and a formal psychiatric screening tool administered (MINI International Neuropsychiatric Interview)[18]. Additional background measures were administered, including a personality disorder screen (Standardised Assessment of Personality – Self-Report, SAPAS-SR)[19], a measure of estimated intellectual functioning (National Adult Reading Test, NART)[20], and a trauma inventory (Childhood Experiences of Care and Abuse Questionnaire, CECA-Q)[21].

**Intervention**

Participants were randomised to receive active or inactive TMS, as described below. Both groups received two TMS sessions, separated by approximately 4 weeks. A formal script was not used during the sessions, but care was taken to have a



For peer review only - http://bmjopen.bmj.com/site/about/guidelines.xhtml

consistent and neutral approach in terms of patient interactions regarding potential improvements to minimise and standardise placebo effect.

### *Active TMS*

The active treatment consisted of spTMS delivered to M1 including stimuli above resting motor threshold (RMT), thereby causing observable movement of the target limb. The target limb was determined for each participant, defined as the weakest limb (i.e. arm or leg on either side) that caused most significant functional impairment in daily life. The target limb remained unchanged throughout both treatment sessions. The treatment was delivered in 2 phases:

*Phase 1: Measuring resting motor threshold (RMT)*

Single pulses were delivered with a Magstim 200 (Magstim, Whitland U.K.) TMS machine either using a circular coil to the area of M1 corresponding to the hand region of both the symptomatic and non-symptomatic arms, or using a double cone coil to deliver pulses to the M1 area for the legs (for participants with leg weakness only). As double cone coils cannot target left or right legs separately (M1 for both legs are stimulated) the same procedure was repeated twice as if targeting each side individually so that the procedure was the same for legs as it was for arms.

Pulses started at 20% of machine output and increased at increments of 5% until the evoked response (measured by surface electromyography in the first dorsal interosseous of the hand or extensor digitorum brevis of the foot) exceeded 50mcV in 50% of trials using a standardised protocol which allows electromyographic



For peer review only - http://bmjopen.bmj.com/site/about/guidelines.xhtml

detection of RMT at 5-10% of TMS output, below that which will produce a movement of the limb detectable by the patient [22]. This value was recorded as the RMT. As a variable number of pulses was needed to establish RMT in each patient, further pulses were then delivered at an interval of 5-10 seconds so that a total of 100 stimuli were delivered (50 stimuli to the same region of M1 bilaterally), to ensure that all participants received an equal number of stimuli during this phase.

*Phase 2: Supra-threshold (Active) TMS*

A further 20 pulses, again at an interval of 5-10 seconds, were delivered at 120% of RMT, applied to the region of M1 corresponding to the participant's weakest limb. No deliberate effort was made by the TMS deliverer to draw attention to the movement of the target limb. A total of 120 pulses were delivered during each of the two treatment sessions. The total number of 120 stimuli was adopted because 100 stimuli is the minimum required to reliably measure RMT and an extra 20 stimuli were needed for clear supra-threshold stimulation for therapeutic effect. This number has been recommended in standardised protocols for RMT measurement [22].

***Inactive (control) TMS***

The inactive control treatment consisted of spTMS delivered to M1 that was always below RMT, thereby not leading to observable movement of the target limb. Phase 1 was identical to the procedures outlined above for measuring RMT.

*Phase 2: Sub-motor threshold (inactive) TMS*

A further 20 pulses at 80% of RMT were applied to the region of M1 corresponding to the patient's weakest limb. Whilst this constituted 'real' TMS, these stimuli did not



For peer review only - http://bmjopen.bmj.com/site/about/guidelines.xhtml

For peer review only

produce any limb movement. Therefore, the key difference between the treatment conditions was whether stimulation was delivered above or below RMT and the initiation of automatic limb movement or not, respectively. As with the active treatment, a total of 120 stimuli were delivered during each TMS session.

**Changes to protocol during trial**

The original protocol specified that the second TMS session would follow the first within a narrowly defined period (30 +/- 2 days); however, during the course of the trial it became clear that this was too restrictive and therefore not practicable, so the time period permitted between treatment sessions was extended (TMS session 2 to occur 28-50 days after TMS session 1).

**Outcome measures**

Outcome measures were completed before and/or after the first TMS session (baseline), before and/or after the second TMS session and three-months after the first TMS session. The primary outcome measure was patient-rated symptom change assessed with the Clinical Global Impression Improvement (CGI-I) scale [23], given the emerging consensus that patient-rated, subjective symptom improvements are particularly meaningful outcomes in this disorder [16, 17].

A range of secondary outcome measures was also included to assess the feasibility of measuring other relevant outcome domains in this group:

- outcome-rater and carer assessed symptom change (CGI-I scale)
- manual muscle testing (MRC strength scale performed by neurologist)



For peer review only - http://bmjopen.bmj.com/site/about/guidelines.xhtml

- dynamometry (if upper limb weakness present)
- subjective ratings of strength (0-100%) and weakness (1-5) in the weakest/target limb
- somatic symptoms (Patient Health Questionnaire (PHQ)-15) [24]
- depression (PHQ-9) [25]
- overall psychological distress (Core Outcomes in Routine Evaluation – 10, CORE-10) [26]
- quality of life (Short-Form Health Survey – 36, SF-36) [27]
- anxiety (Generalised Anxiety Disorder Questionnaire – 7 item, GAD-7) [28]
- disability / physical functioning (Barthel Index / Functional Independence Measure and Functional Assessment Measure (FIM/FAM) [29, 30]
- social and occupational functioning (Work and Social Adjustment Scale, WSAS) [31]
- healthcare utilisation (Client Service Receipt Inventory, CSRI) [32]

For peer review only

**Randomisation and blinding**

Randomisation occurred after the initial screening visit, once eligibility and consent had been confirmed. Randomisation was carried out online by the King's Clinical Trials Unit (KCTU) at the Institute of Psychiatry, Psychology and Neuroscience (IoPPN), using block randomisation. Computer-generated randomisation was initiated when the trial outcome-rater (SP) entered the initials and date-of-birth of the participant onto an online system. Randomisation was then conducted automatically and a confidential email with treatment allocation (active or inactive) sent directly to the TMS deliverer (TN). The outcome-rater (SP) remained blind to treatment allocation throughout the study, as did participants.



For peer review only - http://bmjopen.bmj.com/site/about/guidelines.xhtml

After completion of all study visits for each participant, blinding of the outcome-rater and participant were tested with a forced-choice question about which treatment the patient had received (active or inactive). The patient and outcome-rater answered the question independently. At the end of the study, participants were unblinded individually by the Principal Investigator (TN) during debriefing, with the outcome-rater absent from the room. The outcome-rater remained blind to treatment allocation until all outcome data analyses were completed by the trial statistician.

**Safety monitoring**

Adverse events (AEs) were monitored and recorded at each study visit and reported to the Principal Investigator (TN) or Trial Steering Committee as appropriate. Patients were invited to contact the research team at any time during the trial, in case of an AE occurring between visits.

**Statistical analysis**

***Sample size determination***

Published data on TMS in FND indicates an improvement rate of approximately 10%, albeit on the basis of uncontrolled data. As spontaneous recovery rates are very low, a 10% improvement rate in the control arm at 1 month would be a conservative figure. From a previous CBT trial in FND [7], we would expect 30% of eligible patients to decline participation and then 10% to not complete treatment. Hence with alpha=0.05 and 90% power, to detect an improvement rate of 80% in the active treatment arm relative to 10% in the control (z test between two independent proportions), 9 patients would be needed per arm. For 18 patients to complete the



For peer review only - http://bmjopen.bmj.com/site/about/guidelines.xhtml

study, given a 10% drop out, we would need to randomise 20 participants (30 consented). This allows 10% dropout rate to be assessed with an expected 95% CI of 0% to 24%.

***Feasibility parameters***

Data analysis was carried out in R (v.3.2) by the blinded trial statistician (JH) and adopted the intention-to-treat (ITT) principle. The aims of the analysis were to examine trial feasibility parameters as follows:

- recruitment, randomisation and loss to follow-up rates
- tolerance of treatment, safety, treatment fidelity, participant / outcome-rater blinding and patient satisfaction
- estimate treatment effect sizes as potential outcomes of future trials

The analysis primarily consisted of descriptive statistics to summarise the rates of consent and randomisation of eligible patients, study retention, data quality (i.e., completion of outcome measures, missing data) and the acceptability of TMS to the patient population. Participant demographic and clinical characteristics were also described at baseline.

To assess improvement in symptoms, estimates of treatment effect sizes and 95% confidence intervals on the primary outcome measure (patient-rated CGI-I scale) were obtained using Cliff's Delta as this scale is ordinal. Cliff's Delta is a functional equivalent to Cohen's d for ordinal data, which does not make assumptions about



For peer review only - http://bmjopen.bmj.com/site/about/guidelines.xhtml

the shape or spread of the distribution. In this analysis, Cliff's delta represents the mean between-group difference of within-group change. The effect size values can be interpreted as reflecting the number of times a value in one distribution (active group) is higher than the value in the other distribution (inactive group). Criteria for interpreting the effect size were given by Romano et al. [33], with delta < 0.147 being negligible, delta < 0.33 small, delta < 0.474 being medium and otherwise large. For the secondary outcomes, descriptive statistics and effect sizes were calculated as appropriate for the type of data. Effect sizes (and 95% CI) for secondary outcomes were presented as Cohen's d or Cliff's delta as appropriate to the outcome data.

## RESULTS

### Sample characteristics

#### *Demographics*

The demographic characteristics of participants at enrolment to the study are displayed in Table 1. The average age in each group was similar and the majority of participants in both groups was female, right-handed, married/cohabiting, and most often of white or black British ethnicity. Participants were most likely to report holding an undergraduate degree or vocational qualification. Participants were most often unemployed, but a proportion of patients reported being retired due to ill-health or employed full-time.

#### *Background / clinical characteristics*

Table 2 shows key background and clinical features of participants at entry to the study. The MINI screen identified one patient with possible current psychosis, who



For peer review only - http://bmjopen.bmj.com/site/about/guidelines.xhtml

was subsequently excluded and referred to appropriate clinical services. In eligible patients, the most common comorbid mental health diagnoses identified were major depressive disorder (n=8, 38%) and post-traumatic stress disorder (n=6, 29%). A larger proportion of the inactive group reported additional FND symptoms (i.e., other than limb weakness), relative to the active group. The duration of time since diagnosis was longer for the inactive group, although the duration since symptom onset was similar across groups. A similar proportion of patients in each group reported concurrent interventions at entry to the study and the average number of medications taken was approximately equal. Full details of concomitant treatments are provided in Supplementary File 2. All participants in both groups were taking medication at every time point, with the most common medications being antidepressant, anti-epileptic, anxiolytic and analgesic. The second most common intervention received was physiotherapy (outpatient or during inpatient hospital stays). A small proportion of participants received additional input from occupational therapy, psychology, psychiatry, specialist neurorehabilitation or inpatient hospital (general/neurology) services during the trial.

For peer review only

## Table 1 – Participant demographic characteristics

| | | Active TMS (N=10) | Inactive TMS (N=11) |
|---|---|---|---|
| **Age (Median, interquartile range)** | | 38 (32.5, 46.5) | 41 (33.5, 51) |
| **Gender** | **Female** | 8 (80) | 10 (90.9) |
| | **Male** | 2 (20) | 1 (9.1) |
| **Marital Status** | **Single** | 5 (50) | 3 (27.3) |
| | **Cohabiting / Married** | 5 (50) | 7 (63.6) |
| | **Separated / Divorced** | 0 (0) | 1 (9.1) |
| **Qualifications** | **None** | 0 (0) | 1 (9.1) |
| | **GCSE** | 4 (40) | 1 (9.1) |
| | **A Levels** | 1 (10) | 0 (0) |
| | **Graduate** | 3 (30) | 3 (27.3) |
| | **Postgraduate** | 0 (0) | 1 (9.1) |
| | **Vocational** | 2 (20) | 5 (45.5) |
| **Employment** | **Full Time** | 1 (10) | 3 (27.3) |
| | **Part Time** | 2 (20) | 0 (0) |
| | **Unemployed** | 7 (70) | 4 (36.4) |
| | **Retired (ill health)** | 0 (0) | 4 (36.4) |
| **Handedness** | **Right** | 8 (80) | 8 (72.7) |
| | **Left** | 2 (20) | 2 (18.2) |
| | **Ambidextrous** | 0 (0) | 1 (9.1) |
| **Ethnicity** | **White British** | 5 (50) | 7 (63.6) |
| | **Irish** | 1 (10) | 0 (0) |
| | **White and Black Caribbean** | 0 (0) | 1 (9.1) |
| | **Mixed** | 1 (10) | 0 (0) |
| | **Black British** | 2 (20) | 2 (18.2) |
| | **Caribbean** | 0 (0) | 1 (9.1) |
| | **Other** | 1 (10) | 0 (0) |

**Key:** TMS=transcranial magnetic stimulation

For peer review only



For peer review only - http://bmjopen.bmj.com/site/about/guidelines.xhtml

**Table 2 – Background/clinical characteristics by treatment group**

| | Active TMS (n=10) | Inactive TMS (n=11) |
|---|---|---|
| **SAPAS-SR Total scores (median, IQR)** | 3 (2, 4.8) | 3 (2, 4) |
| **NART estimated IQ scores (median, IQR)** | 107 (105, 113) | 108 (108, 112) |
| **Psychiatric comorbidity present (baseline) (n, %)** | 6 (60) | 5 (45.5) |
| **Other FND symptoms (baseline) (n, %)** | 5 (50) | 9 (81.8) |
| **Age at FND onset, years (median, IQR)** | 35 (28.25, 45) | 31 (23.5, 48.5) |
| **Duration of FND, months (baseline) (median, IQR)** | 41 (14.75 ,63) | 42 (37, 107.5) |
| **Duration since FND diagnosis, months (baseline) (median, IQR)** | 1 (0, 5.25) | 12 (0.5, 38.5) |
| **Number of current medications (median, IQR)** | | |
| **Baseline** | 3 (2.25, 11) | 4 (3.5, 6) |
| **TMS session 1** | 3 (2, 11) | 4 (3.5, 6.5) |
| **TMS session 2** | 7 (2.25, 12.5) | 4.5 (3.25, 6.5) |
| **Follow-up** | 3 (2, 12) | 5 (3.5, 7) |
| **Concurrent treatments (n, %)** | | |
| **Baseline** | 10 (100) | 10 (100) |
| **TMS session 1** | 10 (100) | 9 (100) |
| **TMS session 2** | 6 (100) | 8 (100) |
| **Follow-up** | 9 (100) | 9 (100) |

**Key:** FND=functional neurological disorder; IQR=interquartile range; MDD=major depressive disorder; MINI=MINI International Neuropsychiatric Interview; NART=National Adult Reading Test; PTSD=post-traumatic stress disorder; SAPAS-SR=Standardised Assessment of Personality Abbreviated Scale–Self-Report; TMS=transcranial magnetic stimulation

## Feasibility parameters

Figure 1 displays rates of recruitment, treatment allocation, completion of the study and participants included in the data analysis (CONSORT flow diagram).

<insert Figure 1>

### *Recruitment, attendance and completion*

Of 32 potential candidates referred to the study, 22 consented to participate. Of these, 21 were found to be eligible at baseline screening. All 21 eligible patients were randomised and attended the first TMS treatment session. A total of five



For peer review only - http://bmjopen.bmj.com/site/about/guidelines.xhtml

For peer review only

patients did not attend the second TMS session (active=4; inactive=1), none gave reasons directly related to the intervention (Figure 1). At follow-up, two patients did not attend (active=1; inactive=1). Recruitment of the target number of participants (n=20) was completed within six months. The final follow up session took place approximately nine months after commencement of the study.

***Data quality***

For each visit, the percentage return for each outcome measure was calculated in relation to the number of patients who attended that session (Supplementary File 3). Completion rates for the primary outcome measure (patient-rated CGI-I scale) was 100% at all timepoints. For most other measures, return rates were between 90-100% (i.e., outcome-rater CGI-I scale, Barthel Index, GAD-7, PHQ-9, PHQ-15, WSAS, CORE-10, most SF-36 subscales). A small number of scales were completed less consistently, although rates were still above 80% (e.g., SF-36 Role Emotional at TMS session 1, patient strength ratings/dynamometry at follow-up). Two measures were completed infrequently (carer-rated CGI-I scale/FIM-FAM) in 25% or fewer of the attendees at each timepoint.

For peer review only

***Blinding***

There were no unexpected compromises to blinding during the study procedures. When asked with a forced-choice question at the end of the study, the active treatment was more likely to be correctly guessed as active by both patients (40%) and the outcome-rater (50%), compared to the inactive treatment (patients=36%; outcome-rater=27%). The percentage of correct responses by either informant was not above chance.



For peer review only - http://bmjopen.bmj.com/site/about/guidelines.xhtml

### *Patient satisfaction*

Patients' ratings of their overall experiences of the trial were good. The majority of patients (76%) stated that they were either 'somewhat' or 'very' satisfied with the trial, although ratings were higher in the inactive group (active=60%, inactive=92%). None of the patients in either group reported being 'unsatisfied' (neither 'somewhat' nor 'very'). Qualitatively, patients reported feeling pleased with the level of support and information provided by the research team, felt valued, found assistance with travel arrangements beneficial, and were pleased to be part of a study that could help people with FND more broadly. For some patients, lack of improvement and/or unwanted side effects were noted in the feedback to explain less positive satisfaction ratings (i.e., 'neither satisfied nor unsatisfied').

### *Adverse events*

There were four serious adverse events (SAEs) reported during the study (active=3; inactive=1). One SAE occurred between TMS session 1 and 2, and the other three occurred between TMS session 2 and follow-up. There were no SAEs immediately following a TMS session and none of the SAEs were considered related to the treatment by the Trial Steering Committee. In total there were 78 (non-serious) adverse events (AEs) with 15 of these occurring before the first treatment session. Following the start of treatment, there were 26 AEs in the active group and 37 in the inactive group. A proportion of patients in each group reported headaches at some time during the trial (inactive n=5; active n=3). Worsening of FND symptoms was reported by some patients in each group at one or more time point (inactive n=15; active n=12).

For peer review only

**Primary outcome: patient-rated CGI-I scores**

Figure 2 displays the patient-rated CGI-I scores by group. Immediately prior to TMS session 1, 1 participant (9%) in the inactive group and 0% of the active group rated their symptoms as 'much improved' relative to their condition at entry to the study. Immediately after TMS session 1, these ratings remained the same. Immediately prior to TMS session 2, 67% of patients in the active group and 20% in the inactive group reported that their symptoms were 'much improved'. The relative percentage of 'much improved' again remained the same immediately following TMS session 2. Finally, at three-month follow-up, the number 'much improved' was 44% in the active group and 20% in the inactive group.

<insert Figure 2>

Effect sizes and 95% confidence intervals (Cliff's Delta) for patient-rated CGI-I scores were calculated. The effect size was positive prior to TMS session 1 reflecting coincidentally worse ratings in the active group (Cliff's delta=0.35 (-0.17, 0.71)). This difference remained the same immediately following TMS session 1 (Cliff's delta=0.35 (-0.15, 0.7)). However, this pattern was reversed by TMS session 2, indicating a benefit for the active treatment with moderate effect sizes pre- (Cliff's delta = -0.35 (-0.73, 0.19)) and post-treatment (Cliff's delta = -0.44 (-0.79, 0.13)). At three-month follow-up there was still an advantage for the active treatment; however, the difference was smaller (Cliff's delta = -0.2 (-0.6, 0.28)), potentially due to a relative improvement in the inactive group.



For peer review only - http://bmjopen.bmj.com/site/about/guidelines.xhtml

### Secondary outcomes

Descriptive statistics, effect sizes and confidence intervals for the secondary outcomes can be found in Supplementary File 4. There was considerable variability in the effect sizes and 95% confidence intervals for these outcomes and so the findings cannot be interpreted conclusively. However, the pattern of findings for the following outcomes suggested a benefit of active TMS: outcome-rater CGI-I scores, psychological distress (CORE-10), aspects of quality of life (SF-36 physical functioning, vitality/energy, role limitations due physical and emotional factors), activities of daily living (Barthel), primary care service use. The following outcomes did not suggest a benefit of active TMS: grip strength (dynamometry), subjective (patient-rated) limb strength, additional physical symptoms (PHQ-15), anxiety (GAD-7), depression (PHQ-9), some aspects of quality of life (SF-36 bodily pain, social functioning, mental health), social/occupational functioning (WSAS), inpatient hospital admissions and total outpatient healthcare contacts.

## DISCUSSION

This novel double-blind RCT of spTMS to M1 for the treatment of functional limb weakness was found to be feasible in terms of key parameters allowing estimation of the effect sizes for key outcome variables, and to inform the planning and implementation of a larger RCT.

### Feasibility

Rates of recruitment and retention were acceptable, with only two patients (10%) failing to complete the follow-up visit. Whilst 5 patients did not attend TMS session 2,



For peer review only - http://bmjopen.bmj.com/site/about/guidelines.xhtml

For peer review only

none of these instances was directly related to the nature of the intervention. Nevertheless, consideration should be given to ways of improving attendance rates at the second TMS session, such as offering the session earlier (e.g., after 1 or 2 weeks) and ensuring that any barriers to attendance are identified and managed in advance.

Completion of outcome measures was generally good with rates of 90-100% for most scales. However, the carer-rated CGI-I scale and the FIM-FAM were not completed frequently. Reasons for the lack of completion of the carer-rated CGI-I related to carers not being present or different carers attending each appointment. In future, a specific carer could be identified at the start of the study (in consultation with the patient) and ratings could be obtained by telephone, should that carer be absent at specific visits. It became clear that the FIM-FAM was not a suitable measure for this study, because it requires completion on an inpatient basis, usually by one or more members of a multidisciplinary clinical team. In this study, patients were recruited from a range of outpatient and inpatient settings, and ratings from inpatient clinical teams were at times difficult to obtain. Furthermore, several items on the measure replicated similar constructs assessed within other measures used in the trial (i.e., Barthel, SF-36).

Blinding appeared to be successful, with correct identification of active treatment below chance for both the patients and outcome-rater at the end of the study. Patient satisfaction ratings were also encouraging, suggesting that the trial procedures and the intervention were acceptable in this population. There were no SAEs directly related to the intervention and rates of potentially related AEs (i.e., headaches, FND



For peer review only - http://bmjopen.bmj.com/site/about/guidelines.xhtml

symptom worsening) were not reported at higher rates in the active group. Adverse events should be closely monitored in future studies.

## Outcomes

### *Primary outcome – patient-rated symptom improvement*

Point estimates for the patient-reported symptom improvement showed superiority for the active spTMS intervention relative to the inactive intervention, with small to moderate effect sizes. Improvements were most apparent at TMS session 2 but were still evident at follow-up. It is notable that the pattern of scores on the outcome-rater CGI-I scale concurred with the patient-rated CGI-I scores. These findings suggest that tailored spTMS, delivered above RMT to the area of M1 corresponding to a target limb (i.e., that limb which is functionally weakest) and thus causing movement of that limb, potentially could lead to greater improvements than the same intervention delivered below RMT (i.e., not inducing observable movement). These results concur with those of other studies [11-15] which have previously shown improvements in subjective or objective measures of functional motor symptom severity following spTMS or rTMS to M1.

The mechanism(s) by which TMS to M1 yields improvements in functional motor symptoms is unclear. It is possible that a neuromodulatory mechanism may operate in protocols using rTMS and/or that a general placebo effect could be responsible for improvements in cases where patients/outcome assessors are not blind to treatment allocation. However, similarly to Garcin et al. [12], our study suggests that elicitation of normal function of the weak limb with minimal doses of spTMS is sufficient to induce improvements, at least in the short-term. Induction of observable normal



For peer review only - http://bmjopen.bmj.com/site/about/guidelines.xhtml

function in the limb might result in modification of patients' beliefs and expectations regarding limb functioning and the possibility of recovery, and/or may represent a form of motor retraining effect. It is notable that the improvements did not occur immediately after the first treatment but were instead evident by the second treatment session (pre-TMS), suggesting that whilst one TMS session was sufficient to induce change, the mechanism by which change occurred required time to manifest as symptom reduction.

The findings in this study suggest that the patient-rated CGI-I scale is acceptable and sensitive to change as a measure of symptom improvement in FND intervention studies, in accordance with previous findings across treatment modalities and FND symptom types. This measure has recently been recommended as a primary outcome measure in FND treatment studies [17].

***Secondary outcomes***

High rates of completion of most of the secondary outcome measures indicated that they are appropriate tools for use in future, similar studies. Of the range of outcome domains included, the clearest trends for intervention-related improvements were in activities of daily living/disability (Barthel), overall psychological distress (CORE-10), aspects of health-related quality of life (i.e., physical functioning, physical role, vitality, emotional role) and primary care service use. Whilst extreme caution should be exercised in interpreting these findings due to the small sample size, small-moderate effect sizes and variable confidence intervals, these initial findings suggest that active spTMS might be associated with improvements in aspects of mental health, daily functioning (i.e., roles, daily activities, physical) and treatment seeking,



For peer review only - http://bmjopen.bmj.com/site/about/guidelines.xhtml

in addition to core FND symptom improvements. This extends the findings of previous studies, which have generally demonstrated improvements in functional motor symptoms only. However, it is not possible to say whether improvements in these additional outcome domains followed or preceded motor symptoms.

**Strengths and limitations**

A key strength of this study included the use of a minimal TMS protocol (two brief sessions of spTMS only), which was acceptable to patients and therefore resulted in good treatment adherence rates. This minimal TMS protocol also has potential to be used as a widely accessible treatment that could be used as adjunct to other therapies in a range of settings.

Another strength was that our inactive intervention was similar enough to the active treatment (i.e., 'real' TMS) to reduce the risk of patients inadvertently becoming unblinded to treatment allocation. Furthermore, blinding of both patients and outcome assessors ensured that post-treatment gains were not due entirely to general placebo effects. The inclusion of patients with additional functional neurological symptoms, non-major psychiatric comorbidities and those undergoing concomitant treatments yielded a sample that was representative of the broader FND patient population, improving the generalisability of the findings.

However, it is possible that the additional interventions that some patients were undergoing (e.g., physiotherapy, specialist neurorehabilitation) may have facilitated some of the improvements reported following treatment. Future RCTs with larger samples should balance the influence of concomitant treatments and/or any



For peer review only - http://bmjopen.bmj.com/site/about/guidelines.xhtml

incidental baseline between-group differences in symptoms, background features, or other relevant variables.

Another limitation to note is that some degree of improvement in FND symptoms was observed in both groups prior to commencing the first TMS session, relative to enrolment to the study. It is therefore unclear whether the improvements observed following TMS reflected the effect of the intervention (including its anticipation) or the natural course of the disorder. The lack of a formal script during treatment sessions might have led to inconsistencies in placebo effect. Future studies might valuably include an additional standard care or waiting-list control group, to examine these factors.

**Conclusion**

The findings suggest that active (supra-motor threshold) spTMS to M1 is a safe, efficient, acceptable, and potentially effective treatment for functional limb weakness, leading to improvements in core symptoms and potentially other important outcome domains. A larger, pilot RCT is now warranted, to obtain a more robust estimate of effect sizes and variability in outcomes for this promising intervention.



For peer review only - http://bmjopen.bmj.com/site/about/guidelines.xhtml

## FIGURE LEGENDS

**Figure 1 – CONSORT diagram**

**Figure 2 – Patient-rated CGI-I categories by treatment group and timepoint**

## DECLARATIONS

### Ethics approval and consent to participate

The study was reviewed and approved by the London-Stanmore NHS Research Ethics Committee - study reference number 17/LO/0410). All participants provided informed, written consent prior to involvement in the study.

### Consent for publication

Not applicable.

### Competing interests

Not applicable.

### Data availability

All data relevant to the study are included in the article or uploaded as supplementary information

### Funding

TN, SP and the study were funded by a National Institute of Health Research (NIHR) Clinician Scientist Award to TN. This article represents independent research funded by the NIHR. The views expressed are those of the authors and not necessarily those of the NHS, NIHR or the Department of Health and Social Care.



For peer review only - http://bmjopen.bmj.com/site/about/guidelines.xhtml

## Authors' contributions

TN, AP and AD developed the study design. TN wrote the ethics proposal/study protocol, recruited some participants and conducted the TMS sessions. SP recruited and screened participants, conducted baseline and all subsequent outcome assessments, cleaned/entered data, and wrote the first/subsequent drafts of the manuscript. JH conducted the statistical analyses, prepared the CONSORT flow diagram and some sections of the results. BS, KS, JB, HA, IS, and AE conducted clinical strength tests during outcome assessments. All authors contributed to editing of the manuscript for important intellectual content and approved the final version prior to submission.

For peer review only

## REFERENCES

[1] American Psychiatric Association. Diagnostic and statistical manual of mental disorders (DSM-5). 5th edn. Washington: American Psychiatric Association, 2013.

[2] Gelauff J, Stone J, Edwards M*, et al*. The prognosis of functional (psychogenic) motor symptoms: a systematic review. *J Neurol Neurosurg Psychiatry* 2014;**85**(2):220-226.

[3] Anderson KE, Gruber-Baldini AL, Vaughan CG*, et al*. Impact of psychogenic movement disorders versus Parkinson's on disability, quality of life, and psychopathology. *Mov Disord* 2007;**22**(15):2204-2209.

[4] Szaflarski JP, Hughes C, Szaflarski M*, et al*. Quality of life in psychogenic nonepileptic seizures. *Epilepsia* 2003;**44**(2):236-242.

[5] Espay A.J., Aybek S., Carson A.*, et al*. Current concepts in diagnosis and treatment of functional neurological disorders. *JAMA Neurol* 2018;**75**(9):1132-1141.

[6] LaFrance WC, Jr., Baird GL, Barry JJ*, et al*. Multicenter pilot treatment trial for psychogenic nonepileptic seizures: A randomized clinical trial. *JAMA Psychiatry* 2014;**71**(9):997-1005.

[7] Goldstein LH, Chalder T, Chigwedere C*, et al*. Cognitive-behavioral therapy for psychogenic nonepileptic seizures: a pilot RCT. *Neurology* 2010;**74**(24):1986-1994.

[8] Nielsen G, Buszewicz M, Stevenson F*, et al*. Randomised feasibility study of physiotherapy for patients with functional motor symptoms. *J Neurol Neurosurg Psychiatry* 2017;**88**(6):484-490.

[9] Jordbru AA, Smedstad LM, Klungsøyr O*, et al*. Psychogenic gait disorder: a randomized controlled trial of physical rehabilitation with one-year follow-up. *J Rehabil Med* 2014;**46**(2):181-187.

[10] Pollak TA, Nicholson TR, Edwards MJ*, et al*. A systematic review of transcranial magnetic stimulation in the treatment of functional (conversion) neurological symptoms. *J Neurol Neurosurg Psychiatry* 2014;**85**(2):191-197.

[11] Broersma M, Koops EA, Vroomen PC*, et al*. Can repetitive transcranial magnetic stimulation increase muscle strength in functional neurological paresis? A proof-of-principle study. *Eur J Neurol* 2015;**22**(5):866-873.

[12] Garcin B, Mesrati F, Hubsch C*, et al*. Impact of transcranial magnetic stimulation on functional movement disorders: cortical modulation or a behavioral effect? *Front Neurol* 2017;**8**:338.

[13] Garcin B, Roze E, Mesrati F*, et al*. Transcranial magnetic stimulation as an efficient treatment for psychogenic movement disorders. *J Neurol Neurosurg Psychiatry* 2013;**84**(9):1043-1046.



For peer review only - http://bmjopen.bmj.com/site/about/guidelines.xhtml

[14] Taib S, Ory-Magne F, Brefel-Courbon C, *et al*. Repetitive transcranial magnetic stimulation for functional tremor: A randomized, double-blind, controlled study. *Mov Disord* 2019;**34**(8):1210-1219.

[15] McWhirter L, Ludwig L, Carson A, *et al*. Transcranial magnetic stimulation as a treatment for functional (psychogenic) upper limb weakness. *J Psychosom Res* 2016;**89**:102-106.

[16] Nicholson TR, Carson AJ, Edwards MJ, *et al*. Outcome measures for functional neurological disorder – a review of the theoretical complexities. *J Neuropsychiatry Clin Neurosci* 2020;**32**(1):33-42.

[17] Pick S, Anderson D, Asadi-Pooya A, *et al*. Outcome measurement in functional neurological disorder: a systematic review and recommendations. *J Neurol Neurosurg Psychiatry* (in press). doi:10.1136/jnnp-2019-322180.

[18] Sheehan DV, Lecrubier Y, Sheehan KH, *et al*. The Mini-International Neuropsychiatric Interview (M.I.N.I.): the development and validation of a structured diagnostic psychiatric interview for DSM-IV and ICD-10. *J Clin Psychiatry* 1998;**59 Suppl 20**:22-33.

[19] Germans S, Van Heck GL, Moran P, *et al*. The Self-report Standardized Assessment of Personality-abbreviated Scale: Preliminary results of a brief screening test for personality disorders. *Personal Ment Health* 2008;**2**(2):70-76.

[20] Nelson HE. National Adult Reading Test (NART): For the assessment of premorbid intelligence in patients with dementia: Test manual. Nfer-Nelson, 1982.

[21] Bifulco A, Bernazzani O, Moran P, *et al*. The childhood experience of care and abuse questionnaire (CECA. Q): validation in a community series. *Br J Clin Psychol* 2005;**44**(4):563-581.

[22] Mills KR. Magnetic Stimulation of the Human Nervous System. Oxford: Oxford University Press 1999.

[23] Guy W. ECDEU Assessment Manual for Psychopharmacology. Rockville, MD, US: Department of Health, Education, and Welfare, 1976.

[24] Kroenke K, Spitzer RL, Williams JB. The PHQ-15: validity of a new measure for evaluating the severity of somatic symptoms. *Psychosom Med* 2002;**64**(2):258-266.

[25] Kroenke K, Spitzer RL, Williams JBW. The PHQ-9. *J Gen Intern Med* 2001;**16**(9):606-613.

[26] Barkham M, Bewick B, Mullin T, *et al*. The CORE-10: A short measure of psychological distress for routine use in the psychological therapies. *Couns Psychother Res* 2013;**13**(1):3-13.

[27] Ware Jr JE, Sherbourne CD. The MOS 36-item short-form health survey (SF-36): I. Conceptual framework and item selection. *Med Care* 1992;**30**:473-483.



For peer review only - http://bmjopen.bmj.com/site/about/guidelines.xhtml

[28] Spitzer RL, Kroenke K, Williams JB, *et al*. A brief measure for assessing generalized anxiety disorder: the GAD-7. *Arch Intern Med* 2006;**166**(10):1092-1097.

[29] Mahoney FI, Barthel DW. Functional evaluation: the Barthel Index: a simple index of independence useful in scoring improvement in the rehabilitation of the chronically ill. *Md State Med J* 1965;**14**:61-65.

[30] Turner-Stokes L, Nyein K, Turner-Stokes T, *et al*. The UK FIM FAM: development and evaluation. *Clin Rehabil* 1999;**13**(4):277-287.

[31] Mundt JC, Marks IM, Shear MK, *et al*. The Work and Social Adjustment Scale: a simple measure of impairment in functioning. *Br J Psychiatry* 2002;**180**(5):461-464.

[32] Beecham J, Knapp M. Costing psychiatric interventions. Measuring mental health needs.Gaskell, London 2001;200-224.

[33] Romano J, Kromrey JD, Coraggio J, *et al*. Exploring methods for evaluating group differences on the NSSE and other surveys: Are the t-test and Cohen'sd indices the most appropriate choices. In *Annual Meeting of the Southern Association for Institutional Research* 2006;1-51.

For peer review only



For peer review only - http://bmjopen.bmj.com/site/about/guidelines.xhtml

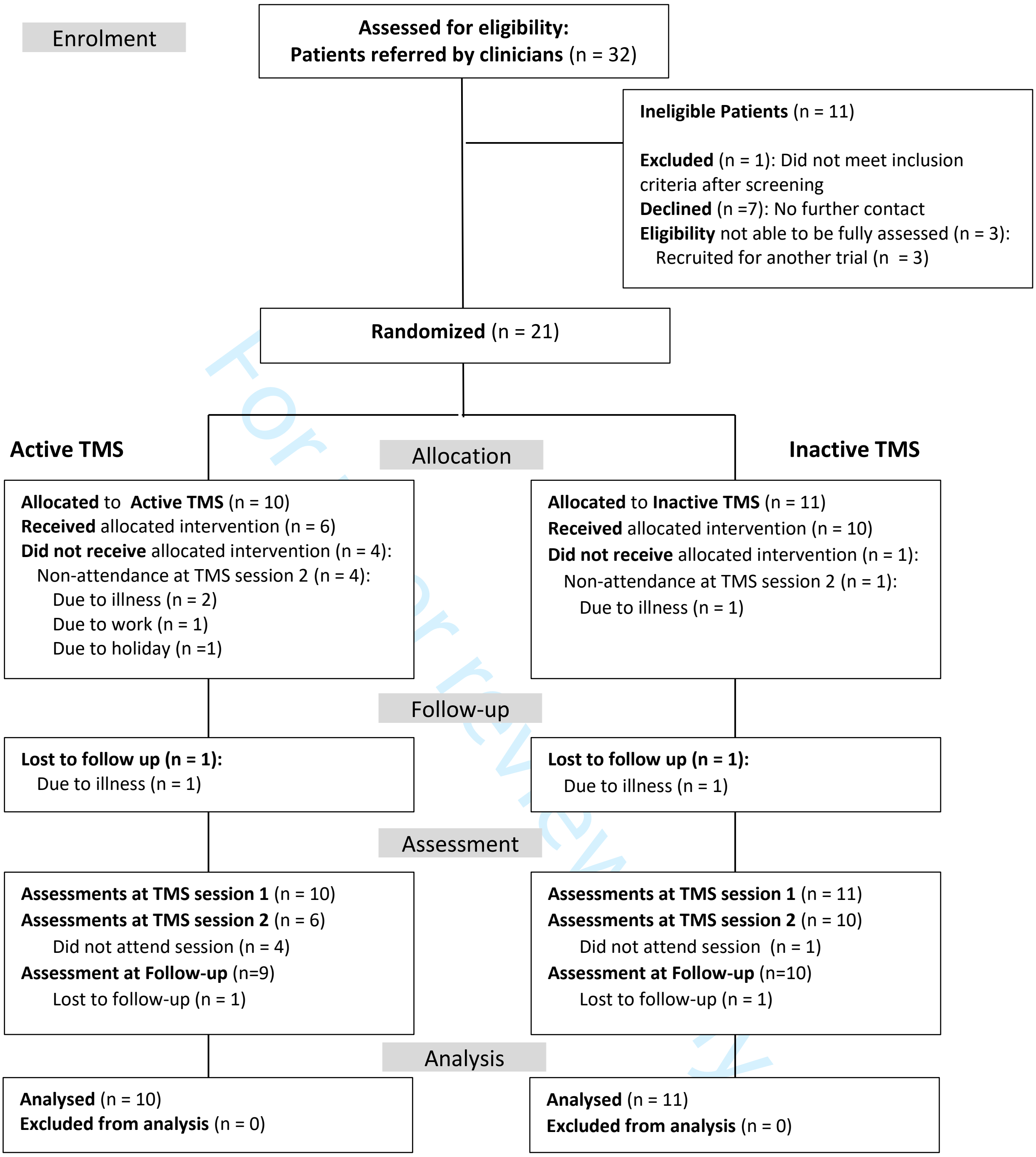


For peer review only - http://bmjopen.bmj.com/site/about/guidelines.xhtml

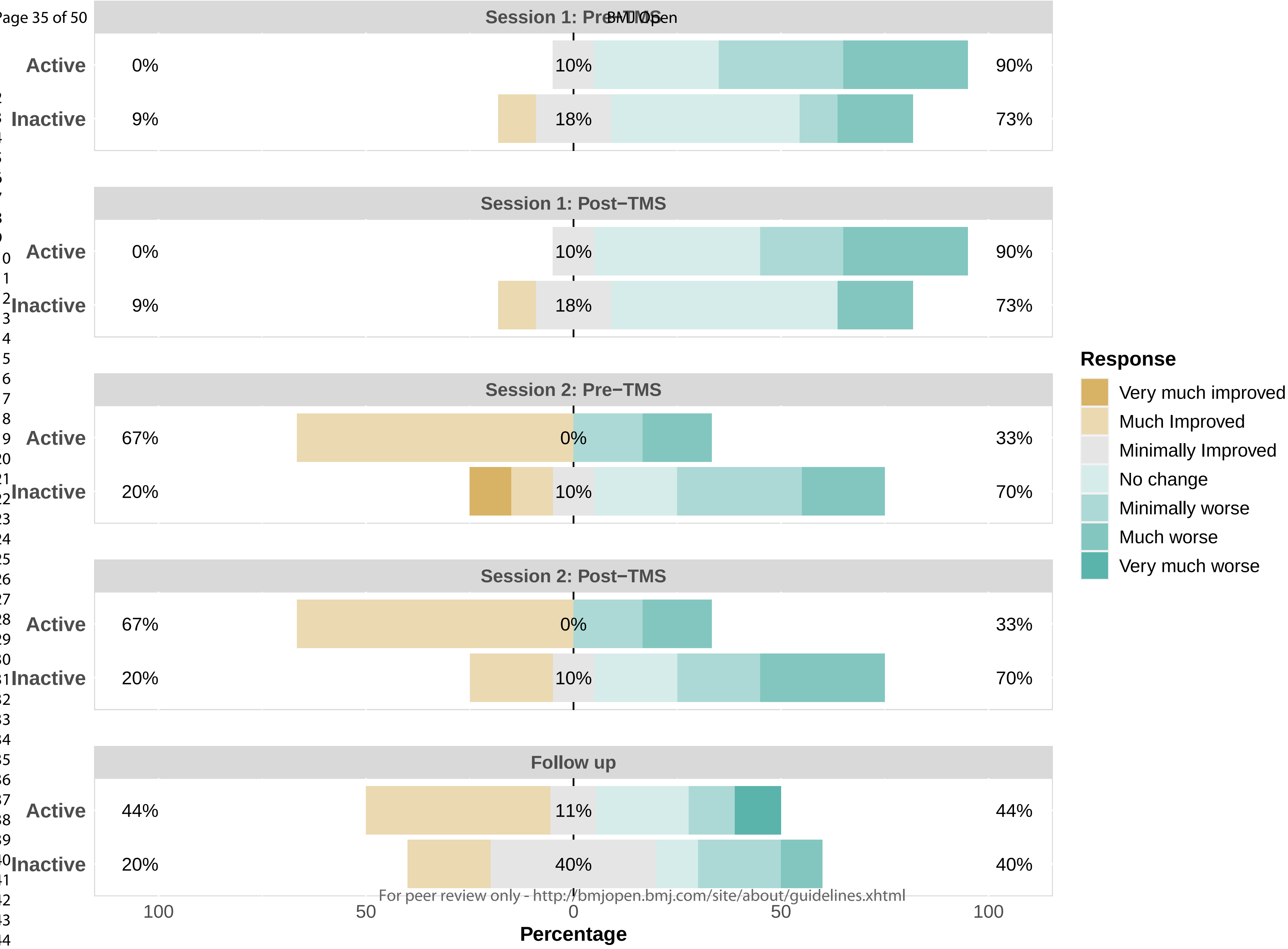

Session 1: Pre−TMS
Active
0%
10%
90%
Inactive
9%
18%
73%
Session 1: Post−TMS
Active
0%
10%
90%
Inactive
9%
18%
73%
Session 2: Pre−TMS
Active
67%
0%
33%
Inactive
20%
10%
70%
Session 2: Post−TMS
Active
67%
0%
33%
Inactive
20%
10%
70%
Follow up
Active
44%
11%
44%
Inactive
20%
40%
40%
100
50
0
50
100
Percentage
Response
Very much improved
Much Improved
Minimally Improved
No change
Minimally worse
Much worse
Very much worse

**Supplementary File 1. Patient Information Sheet**

*There is no potentially identifiable patient information in this document.

# Patient Information Sheet & Consent Form

## Trial Of Neurostimulation In Conversion Symptoms (TONICS):
### A Randomised Controlled Trial (RCT) feasibility study of Transcranial Magnetic Stimulation (TMS) for conversion disorder with motor symptoms

REC reference number 17/LO/0410

You are being invited to take part in a research study. Before you decide, please take time to read the following information and to decide whether or not you would like to participate. If anything is unclear or you would like further information, please ask a member or the research team. Thank you for taking the time to read this.

**What is the purpose of the study?**

This study aims to assess Transcranial Magnetic Stimulation (TMS) as a new potential treatment for conversion disorder (CD), also known as Functional Neurological Disorder (FND). CD is where neurological symptoms, such as weakness, occur but no structural neurological disease can be found – therefore they are disorders of function, rather than structure. There are few proven treatments for weakness that is caused by CD. However, there is encouraging preliminary evidence that TMS could be an effective and safe treatment for such symptoms but until a Randomised Controlled Trial (RCT) is conducted it is not possible to establish whether this is the case.

**What is Transcranial Magnetic Stimulation (TMS)?**

TMS is a form of 'non-invasive brain stimulation', i.e. it is a way of stimulating the brain from outside the head. It works by holding a magnetic coil approximately the size of a small side plate against the head (it rests on the scalp) which then delivers magnetic pulses that stimulate the underlying brain. It was developed over 30 years ago and has been increasingly used treat a number of neurological and psychiatric disorders. It is considered to be a relatively safe and generally well-tolerated treatment. This is a picture of TMS coil being used in our laboratory*:

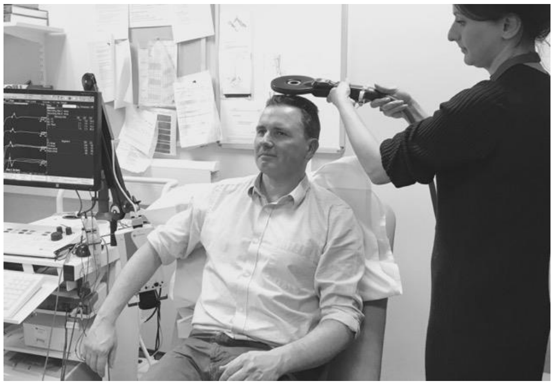

*The individuals depicted in this image are members of the research team, not clinical cases.



For peer review only - http://bmjopen.bmj.com/site/about/guidelines.xhtml

**What is a Randomised Controlled Trial (RCT)?**

Randomised Controlled Trials (RCTs) are the best way to tell whether a treatment really works and each year thousands of people take part in them. The word ‘controlled’ means that a ‘control’ treatment, e.g. an inactive or ‘placebo’ form of the treatment, is used to compare response to the ‘active’ treatment being investigated. This allows us to know whether any improvements (or side effects) are really due to the treatment or could either have occurred due to placebo effects or could have naturally occurred. Therefore patients are allocated to different groups to receive either the active treatment (Group A) or the inactive / placebo treatment (Group B).

The term ‘randomised’ means that people allocated at random to one of these two groups as this is the only way to compare treatments fairly. Randomisation means the chances are exactly equal for being allocated to either group and therefore no-one can predict in advance the group to which you will be allocated, in case this in any way affects what you or we expect to be the outcome of the study. Random allocation could be done using the result of tossing a coin (i.e. ‘heads’ for group A and ‘tails’ for Group B) to decide which treatment you will get but we will do this using a computer.

It is also important, where possible, that patients do no know (i.e. are ‘blind’ to) which treatment they have been allocated to as this can affect response. This means you won’t know which group you’ve been allocated to until *after* you have not only completed the treatment but also completed the follow up interviews and questionnaires which will assess your response to the treatment you received.

For those who were allocated the ‘inactive treatment’ if it is felt after completing the treatment that they might benefit from receiving ‘active’ TMS as well, they will be offered this treatment after finishing the trial.

**Why have I been chosen?**

You have been chosen because you are over 18 and have been diagnosed with CD that is causing weakness in at least one of your limbs – this is known as ‘motor’ CD. As we don’t currently know if TMS is any more helpful to patients than placebo, a Randomised Controlled Trial (RCT) is the most exact and fair way for us to see how helpful TMS really is at improving weakness in motor CD.

**Do I have to take part and can I withdraw from the study if I change my mind?**

It is completely up to you to decide whether or not to take part. You may consider this at your leisure, and contact us for more information, at the number below or arrange to discuss the study with a member of the research team. If you do decide to take part you will still be free to withdraw at any time and without giving a reason and this will not affect the standard of care you receive now or in the future. We would not collect any new information on you. However, any information that we had already collected would be kept by the study team.

**What will happen to me if I take part?**

If you decide to take part then a research worker will arrange to meet with you at a time that is convenient for you. At the appointment the research worker will explain the study to you in more detail, check you are eligible for the study and answer any questions that


For peer review only - http://bmjopen.bmj.com/site/about/guidelines.xhtml

you may have. We will give you another copy of this Information Sheet to keep and ask you to sign a consent form.

The research worker will then collect some simple information on things such as your age, previous medical history, current medications and employment history. They will undertake an assessment of any psychological problems that you may have and ask you to complete a number of questionnaires. In total this will take about 1.5 hours.  and will explain how treatment might help you. They will also carefully check that it is safe to give you TMS treatment, such as whether you have seizures (specifically epileptic seizures).

You will then be randomly assigned to either Group A, where you will receive the active treatment, or to Group B where you will receive the inactive treatment. The randomisation will be done by someone who does not know you and who is not directly involved in the study.

You will then be invited for the first treatments session. The treatment itself will take about 30 minutes and beforehand your strength will be tested by a member of the research team and you will be asked to fill in some more questionnaires about your current symptoms and health which will take approximately another 60 minutes so the whole session will take about an hour and a half.  You will then be invited back for another identical treatment session 1 month later. Another 2 months later, so 3 months after the first session, you will be invited for a final session – this time with no treatment but just the examination and questionnaires. All these sessions will be arranged at a time to suit you and we will provide your transport costs.

**How long will I be in the study?**

If you agree to take part in this part of the study it will take 3 months from the start of treatment until the completion of the last follow up session.

**What are the possible risks of taking part?**

There are some risks to taking part in the study as TMS can cause side effects. The most common side effect is that some people can find the TMS treatment uncomfortable around the area it is delivered to (the scalp) and for some this experience is painful but the vast majority of people given the type of TMS in this study find it tolerable.

It can also cause headaches which generally resolve soon after the treatment is given. Very rarely it can cause seizures – but is only reported to occur with higher 'doses' of TMS than used in this study and only in those with, or predisposed to, epilepsy - which is why this is carefully screened for beforehand.

It is also possible that some of the questionnaires you will be asked to fill might cause you distress to answer as they ask about you past psychiatric history and if you have suffered from any abuse. If you experience any of these issues you can discuss them with a member of the research team or your GP and re-evaluate whether you want to continue with the study or not.

**What are the possible benefits of taking part?**

By taking part in the study you will help us understand more about treatments that are effective in helping people with weakness caused by CD.  We cannot be sure at this stage whether the active TMS will be any more effective than the inactive TMS and


For peer review only - http://bmjopen.bmj.com/site/about/guidelines.xhtml

therefore whether you will personally benefit, regardless of which group you are allocated to.

**Will taking part or not influence my medical care?**

Your participation will have no influence on your medical care. There will be no restrictions on your diet or lifestyle during the study. Any doctors or other healthcare professionals you see can make any changes to your medication or other treatments that they feel are necessary for you. Similarly, as mentioned above, not taking part will have no influence on any aspect of your care.

**What expenses will be covered?**

Whichever group you are allocated to, we will pay for your travel up to a maximum £25 for each assessment that is necessary. However, if you take time off work to attend the study appointments we cannot pay you or your employer for this.

**Will my taking part in this study be kept confidential?**

All information which is collected about you during the course of the research will be kept strictly confidential. The research workers who contact you will need to keep your contact details at the university research sites, but only for the purposes of contacting you about arranging to see you or to send you questionnaires. Any other information about you will have your name and address removed so that you cannot be recognised from it. We will not identify you in our computers or publications by name, and will only refer to you by participant number, which will be used in place of your name on any future publications. All information will be stored on password protected computers and paperwork will be stored securely in locked university offices.

If you take part in this Randomised Controlled Trial (RCT) we will ask if we can contact you, perhaps through your GP, if you move house during our study. With your permission we would want to inform your GP that you are taking part in the study and potentially also see your medical file. We would also need to inform your GP or other professionals if one of the health professionals or research workers in the study became concerned about your well-being or about the implications of what you tell us for someone else's well-being. We would of course discuss this with you if such a situation arose.

**What will happen if new information becomes available?**

Sometimes during the course of a study new information might become available about the treatment that is being tested. If this happens, either your medical doctor or a member of the research team will contact you and arrange to talk to you about this and discuss with you whether you want to continue. If you decide to withdraw from the study your doctor will make arrangements for your care to continue. If you decide to continue in the study you may be asked to sign an updated consent form.

**What happens when the trial is over?**

Once the trial is over, we will see whether the active TMS has helped people reduce their weakness any more than the inactive TMS. If you did not receive active TMS during the study then the doctors treating you will decide whether you might still benefit from this and if so they will refer you for this treatment.

**What happens if something goes wrong?**


For peer review only - http://bmjopen.bmj.com/site/about/guidelines.xhtml

We do not expect there to be any significant adverse effects from taking part in this study. However if you are harmed during the study and this is due to someone's negligence, then you may have grounds for legal action for compensation against the NHS but you may have to pay your legal costs. Regardless of this, if you wish to complain, or have any concerns about any aspect of the way you have been approached or treated during the course of this study, the normal National Health Service complaints mechanisms should be available to you.

King's College London holds insurance policies that apply to this study. If you experience harm or injury as a result of taking part in this study you may be eligible to claim compensation without having to prove that King's College London is at fault. This does not affect your legal rights to seek compensation.

If for any reason your symptoms get much worse during the study, then you will be able to talk to your medical specialist or any of us who are involved in the study and discuss what you want to do.

**What happens to the results of the research study?**

We will publish the results of the research in scientific journals and we will present the results at scientific meetings. In addition we will talk to service providers about the results of our research. We will not identify you in any report/publication. If you would like a copy of the published results, we can provide this at the end of the study.

**How often will I be contacted by the investigators?**

We will need to contact you at different stages of the study to arrange treatment or follow up sessions and will give you two reminders to let us have this information. If at any particular stage you change your mind about taking part in the study and we do not hear from you at all, we will contact you on only one further occasion to discuss the study. If we cannot discuss this with you we will assume you have chosen to leave the study. We can reassure you that you will not be contacted repeatedly if you decide you no longer wish to be part of the study. If you then change your mind about letting us have the information we asked for, you can contact us by phone, letter or email to then re-join the study if you wish.

**Can my participation in the study be discontinued by the investigators?**

Yes. At any time during the study, the investigators have the right to end your participation in the study for any reason. If so, this reason will be explained to you. If later on in the study it is concluded that you no longer have capacity to consent to participating we would like to be able to continue to use any data that we have already collected, in an anonymised form.

**Who is organized, funded and reviewed the research?**

The research is funded by the National Institute of Health Research, and administered by the Institute of Psychiatry Psychology & Neuroscience, part of King's College London. The study has been reviewed and approved by a UK Research Ethics Committee (London-Stanmore Research Ethics Committee - study reference number 17/LO/0410).



For peer review only - http://bmjopen.bmj.com/site/about/guidelines.xhtml

**Contacts for Further Information**

If you require any further information, please contact Dr Nicholson or a member of the research team at the Section of Cognitive Neuropsychiatry (PO68), Institute of Psychiatry Psychology & Neuroscience, De Crespigny Park, London SE5 8AF
Tel: 0207 848 5136 Fax: 0207 848 0572 Email timothy.nicholson@kcl.ac.uk.

You will be given a copy of this information sheet and a signed consent form to keep.

---

If you would like any independent advice about taking part in a research study, or have concerns about the conduct of the study, please contact your Trust Patient Advice and Liaison Service (PALS). PALS offers free confidential advice, support and information on health-related matters and are independent of clinical services. They provide a point of contact for patients, their families and their carers. PALS also helps to improve the NHS by listening to your concerns and suggestions. You can find your nearest PALS on the NHS Choices website: http://www.nhs.uk/Service-Search/Patient-advice-and-liaison-services-(PALS)/LocationSearch/363

Local PALS offices are also listed below:

**South London & Maudsley NHS Foundation Trust (SLAM) PALS**
Website: http://www.slam.nhs.uk/patients-and-carers/advice-and-information
Email: pals@slam.nhs.uk
Phone: 0800 731 2864 (freephone number)

**King's College Hospital NHS Foundation Trust (KCH) PALS**
Online contact form: https://www.kch.nhs.uk/contact/pals
Email: kch-tr.PALS@nhs.net
Phone: 020 3299 3601, 9am to 4.30pm, Monday to Friday (not bank holidays)

**Guys and St Thomas' NHS Foundation Trust (GST) PALS**
Online contact form: http://www.guysandstthomas.nhs.uk/contact-us/feedback-forms/Questions-about-care.aspx
Email: pals@gstt.nhs.uk
Phone: 020 7188 8801


For peer review only - http://bmjopen.bmj.com/site/about/guidelines.xhtml

## Supplementary File 2

*Table 2.1. Concomitant treatments by group and timepoint**

| Treatment | Baseline | TMS Visit 1<br>n (%) | TMS Visit 2<br>n (%) | Follow up<br>n (%) |
|---|---|---|---|---|
| **Medication** | Active=10 (100)<br>Inactive=10 (100) | Active=10 (100)<br>Inactive=9 (100) | Active=6 (100)<br>Inactive=8 (100) | Active=9 (100)<br>Inactive=9 (100) |
| **Physiotherapy** | Active=4 (40)<br>Inactive=2 (20) | Active=4 (40)<br>Inactive=2 (2) | Active=0 (0)<br>Inactive=1 (13) | Active=1 (11)<br>Inactive=1 (11) |
| **Neurology inpatient** | Active=0 (0)<br>Inactive=0 (0) | Active=1 (10)<br>Inactive=0 (0) | Active=0 (0)<br>Inactive=0 (0) | Active=0 (0)<br>Inactive=0 (0) |
| **General inpatient** | Active=3 (30)<br>Inactive=1 (10) | Active=2 (20)<br>Inactive=1 (11) | Active=0 (0)<br>Inactive=1 (13) | Active=0 (0)<br>Inactive=0 (0) |
| **Specialist MDT inpatient neurorehabilitation** | Active=1 (10)<br>Inactive=1 (10) | Active=1 (10)<br>Inactive=1 (11) | Active=1 (17)<br>Inactive=1 (13) | Active=1 (11)<br>Inactive=1 (11) |
| **Specialist MDT day hospital** | Active=0 (0)<br>Inactive=1 (10) | Active=0 (0)<br>Inactive=1 (11) | Active=0 (0)<br>Inactive=0 (0) | Active=1 (11)<br>Inactive=0 (0) |
| **CBT / Psychology** | Active=2 (20)<br>Inactive=1 (10) | Active=2 (20)<br>Inactive=1 (11) | Active=1 (17)<br>Inactive=0 (0) | Active=1 (11)<br>Inactive=2 (22) |
| **Occupational therapy** | Active=2 (20)<br>Inactive=1 (10) | Active=2 (20)<br>Inactive=1 (11) | Active=1 (17)<br>Inactive=1 (13) | Active=1 (11)<br>Inactive=0 (0) |
| **Psychiatry (outpatient)** | Active=0 (0)<br>Inactive=0 (0) | Active=0 (0)<br>Inactive=0 (0) | Active=0 (0)<br>Inactive=1 (13) | Active=1 (11)<br>Inactive=1 (11) |

**Key:** CBT=cognitive behavioural therapy; MDT=multidisciplinary team; TMS=transcranial magnetic stimulation

For peer review only

## Supplementary File 3 – Outcome measure completion data

### *Table 3.1. Data quality by timepoint**

| Outcome measure | TMS Visit 1 n (%) | TMS Visit 2 n (%) | Follow up n (%) |
|---|---|---|---|
| **CGI Patient** | 21 (100) | 16 (100) | 19 (100) |
| **CGI Outcome assessor** | 21 (100) | 16 (100) | 20 (105) |
| **CGI Carer** | 2 (10) | 4 (25) | 4 (21) |
| **SF36: Physical Function** | 21 (100) | 16 (100) | 19 (100) |
| **SF36: Role Physical** | 20 (95) | 16 (100) | 19 (100) |
| **SF36: Bodily Pain** | 21 (100) | 16 (100) | 19 (100) |
| **SF36: General Health** | 21 (100) | 16 (100) | 19 (100) |
| **SF36: Vitality** | 21 (100) | 16 (100) | 19 (100) |
| **SF36: Social Functioning** | 21 (100) | 16 (100) | 19 (100) |
| **SF36: Role Emotional** | 18 (86) | 16 (100) | 19 (100) |
| **SF36: Mental Health** | 21 (100) | 16 (100) | 19 (100) |
| **Barthel Index** | 21 (100) | 16 (100) | 20 (105) |
| **FIM-FAM** | 4 (19) | 2 (12) | 2 (11) |
| **GAD 7** | 21 (100) | 16 (100) | 19 (100) |
| **PHQ 9** | 21 (100) | 16 (100) | 19 (100) |
| **PHQ 15** | 21 (100) | 16 (100) | 19 (100) |
| **CORE-10** | 21 (100) | 16 (100) | 19 (100) |
| **WSAS** | 21 (100) | 16 (100) | 19 (100) |
| **Left Arm; Strength** | 20 (95) | 15 (94) | 17 (89) |
| **Left Arm: Weakness** | 20 (95) | 15 (94) | 18 (95) |
| **Right Arm: Strength** | 20 (95) | 15 (94) | 17 (89) |
| **Right Arm: Weakness** | 20 (95) | 15 (94) | 18 (95) |
| **Left Leg; Strength** | 21 (100) | 16 (100) | 18 (95) |
| **Left Leg: Weakness** | 21 (100) | 16 (100) | 19 (100) |
| **Right Leg: Strength** | 20 (95) | 15 (94) | 17 (89) |
| **Right Leg: Weakness** | 20 (95) | 15 (94) | 18 (95) |
| **Dynamometry Left Arm: Max** | 20 (95) | 15 (94) | 17 (89) |
| **Dynamometry Left Arm: Max** | 20 (95) | 15 (94) | 18 (95) |
| **Dynamometry Left Arm: Max** | 20 (95) | 15 (94) | 17 (89) |
| **Dynamometry Left Arm: Max** | 20 (95) | 15 (94) | 18 (95) |

**Key:** CGI=Clinical Global Impression; CORE=10=Clinical Outcomes in Routine Evaluation-10 item; GAD-7=Generalised Anxiety Disorder-7 item; KG=kilogram; PHQ=Patient Health Questionnaire; SF-36=Short Form Health Survey-36 item; TMS=transcranial magnetic stimulation; WSAS=Work & Social Adjustment Scale

***Percentages calculated relative to the number of patients in attendance in each group*

For peer review only

# Supplementary File 4 - Descriptive statistics and effect sizes for primary and secondary outcomes

### *Supplementary Table 4.1. Patient CGI-I ratings*

| | | Visit 1 | | | | Visit 2 | | | | Follow-up | |
|---|---|---|---|---|---|---|---|---|---|---|---|
| | | Pre-TMS | | Post-TMS | | Pre-TMS | | Post-TMS | | | |
| | | Active | Inactive | Active | Inactive | Active | Inactive | Active | Inactive | Active | Inactive |
| Very much improved | n (%) | 0 (0) | 0 (0) | 0 (0) | 0 (0) | 0 (0) | 1 (10) | 0 (0) | 0 (0) | 0 (0) | 0 (0) |
| Much improved | n (%) | 0 (0) | 1 (9) | 0 (0) | 1 (9) | 4 (67) | 1 (10) | 4 (67) | 2 (20) | 4 (44) | 2 (20) |
| Minimally improved | n (%) | 1 (10) | 2 (18) | 1 (10) | 2 (18) | 0 (0) | 1 (10) | 0 (0) | 1 (10) | 1 (11) | 4 (40) |
| No change | n (%) | 3 (30) | 5 (45) | 4 (40) | 6 (55) | 0 (0) | 2 (20) | 0 (0) | 2 (20) | 2 (22) | 1 (10) |
| Minimally worse | n (%) | 3 (30) | 1 (9) | 2 (20) | 0 (0) | 1 (17) | 3 (30) | 1 (17) | 2 (20) | 1 (11) | 2 (20) |
| Much worse | n (%) | 3 (30) | 2 (18) | 3 (30) | 2 (18) | 1 (17) | 2 (20) | 1 (17) | 3 (30) | 0 (0) | 1 (10) |
| Very much worse | n (%) | 0 (0) | 0 (0) | 0 (0) | 0 (0) | 0 (0) | 0 (0) | 0 (0) | 0 (0) | 1 (11) | 0 (0) |
| Total | n (%) | 10 (100) | 11 (100) | 10(100) | 11 (100) | 6 (60) | 10 (91) | 6 (60) | 10 (91) | 9 (90) | 10 (91) |
| Missing* | n (%) | 0 (0) | 0 (0) | 0 (0) | 0 (0) | 4 (40) | 1 (9) | 4 (40) | 1 (9) | 1 (10) | 1 (9) |
| Effect size (negative = benefit) | Cliff's delta (95% CI) | 0.35 (-0.17, 0.71) | | 0.35 (-0.15, 0.7) | | -0.35 (-0.73, 0.19) | | -0.44 (-0.79, 0.13) | | -0.2 (-0.6, 0.28) | |

**Key:** CGI-I=Clinical Global Impression-Improvement; CI=confidence interval; TMS=transcranial magnetic stimulation

******Percentage calculated relative to total number of participants enrolled in study*

For peer review only

***Supplementary Table 4.2. Outcome assessor CGI-I ratings***

| | | Visit 1 | | | | Visit 2 | | | | Follow-up | |
|---|---|---|---|---|---|---|---|---|---|---|---|
| | | Pre-TMS | | Post-TMS | | Pre-TMS | | Post-TMS | | | |
| | | Active | Inactive | Active | Inactive | Active | Inactive | Active | Inactive | Active | Inactive |
| Very much improved | n (%) | 0 (0) | 0 (0) | 0 (0) | 0 (0) | 0 (0) | 0 (0) | 0 (0) | 0 (0) | 0 (0) | 0 (0) |
| Much improved | n (%) | 0 (0) | 0 (0) | 0 (0) | 0 (0) | 3 (50) | 2 (20) | 3 (50) | 2 (20) | 4 (44) | 2 (18) |
| Minimally improved | n (%) | 1 (10) | 3 (27) | 1 (10) | 3 (27) | 1 (17) | 1 (10) | 1 (17) | 1 (10) | 3 (33) | 5 (45) |
| No change | n (%) | 3 (30) | 7 (64) | 4 (40) | 6 (55) | 0 (0) | 5 (50) | 0 (0) | 5 (50) | 1 (11) | 2 (18) |
| Minimally worse | n (%) | 3 (30) | 1 (9) | 1 (10) | 2 (18) | 2 (33) | 2 (20) | 2 (33) | 2 (20) | 0 (0) | 1 (9) |
| Much worse | n (%) | 3 (30) | 0 (0) | 4 (40) | 0 (0) | 0 (0) | 0 (0) | 0 (0) | 0 (0) | 0 (0) | 1 (9) |
| Very much worse | n (%) | 0 (0) | 0 (0) | 0 (0) | 0 (0) | 0 (0) | 0 (0) | 0 (0) | 0 (0) | 1 (11) | 0 (0) |
| Total | n (%) | 10 (100) | 11 (100) | 10 (100) | 11 (100) | 6 (60) | 10 (91) | 6 (60) | 10 (91) | 9 (90) | 11 (100) |
| Missing* | n (%) | 0 (0) | 0 (0) | 0 (0) | 0 (0) | 4 (40) | 1 (9) | 4 (40) | 1 (9) | 1 (10) | 0 (0) |
| Effect size (negative = benefit) | Cliff's delta (95% CI) | 0.55 (0.05, 0.83) | | 0.45 (-0.06, 0.77) | | -0.29 (-0.69, 0.25) | | -0.29 (-0.69, 0.25) | | -0.26 (-0.65, 0.23) | |

**Key:** CGI-I=Clinical Global Impression-Improvement; CI=confidence interval; TMS=transcranial magnetic stimulation

**Percentage calculated relative to total number of participants enrolled in the study*

For peer review only

*Supplementary Table 4.3. Patient weakness ratings*

| | | Visit 1 | | | | Visit 2 | | | | Follow-up | |
|---|---|---|---|---|---|---|---|---|---|---|---|
| | | Pre-TMS | | Post-TMS | | Pre-TMS | | Post-TMS | | | |
| | | **Active** | **Inactive** | **Active** | **Inactive** | **Active** | **Inactive** | **Active** | **Inactive** | **Active** | **Inactive** |
| **No weakness** | **n(%)** | 2 (20) | 1 (9) | 3 (30) | 1 (9) | 0 (0) | 1 (10) | 1 (17) | 1 (10) | 2 (22) | 0 (0) |
| **Mild weakness** | **n(%)** | 1 (10) | 3 (27) | 0 (0) | 5 (45) | 1 (17) | 4 (40) | 0 (0) | 5 (50) | 1 (11) | 5 (50) |
| **Moderate weakness** | **n(%)** | 1 (10) | 3 (27) | 1 (10) | 0 (0) | 0 (0) | 1 (10) | 1 (17) | 0 (0) | 3 (33) | 1 (10) |
| **Severe weakness** | **n(%)** | 3 (30) | 1 (9) | 3 (30) | 2 (18) | 3 (50) | 2 (20) | 3 (50) | 3 (30) | 1 (11) | 3 (30) |
| **Very severe weakness** | **n(%)** | 3 (30) | 3 (27) | 3 (30) | 3 (27) | 2 (33) | 2 (20) | 1 (17) | 1 (10) | 2 (22) | 1 (10) |
| **Total** | **n(%)** | 10 (100) | 11 (100) | 10 (100) | 11 (100) | 6 (60) | 10 (91) | 6 (60) | 10 (91) | 9 (90) | 10 (91) |
| **Missing*** | **n(%)** | 0 (0) | 0 (0) | 0 (0) | 0 (0) | 4 (40) | 1 (9) | 4 (40) | 1 (9) | 1 (10) | 1 (9) |
| **Effect size (negative = treatment benefit)** | **Cliff's Delta (95% CI)** | 0.09 (-0.41, 0.55) | | 0.04 (-0.46, 0.51) | | 0.27 (-0.11, 0.58) | | 0.17 (-0.25, 0.53) | | -0.08 (-0.51, 0.37) | |

**Key:** CI=confidence interval; TMS=transcranial magnetic stimulation

*****Percentage calculated relative to total number of participants enrolled in the study*

For peer review only

*Supplementary Table 4.4. Additional secondary outcome measures*

| | | Visit 1 | | Visit 2 | | Follow-up |
|---|---|---|---|---|---|---|
| **Measure** | **Statistic** | **Pre-TMS** | **Post-TMS** | **Pre-TMS** | **Post-TMS** | |
| **Target limb strength rating (0-100%)** | **Mean (SD)** | Active=42.5 (37.4)<br>Inactive=52.3 (30.4) | Active=44.5 (40.6)<br>Inactive=52.7 (35) | Active=38.3 (25)<br>Inactive=55 (34) | Active=42.8 (34.1)<br>Inactive=57 (34) | Active=41.9 (27.5)<br>Inactive=51.8 (36.2) |
| | **Cohen's d** | 0.29 (-0.63, 1.21) | 0.22 (-0.7, 1.14) | 0.54 (-0.59, 1.66) | 0.42 (-0.7, 1.53) | 0.3 (-0.71, 1.31) |
| **Dynamometry – left arm (average KG)** | **Mean (SD)** | Active=12.4 (10.8)<br>Inactive=6.1 (6.9) | Active=11.3 (11.7)<br>Inactive=7 (8.9) | Active=11.9 (3.7)<br>Inactive=6.3 (11) | Active=11.6 (6.1)<br>Inactive=6.4 (12) | Active=10.7 (9.1)<br>Inactive=9.7 (12.3) |
| | **Cohen's d** | 0.68 (-0.35, 1.71) | 0.41 (-0.61, 1.42) | 0.65 (-0.6, 1.91) | 0.53 (-0.72, 1.77) | 0.09 (-1.02, 1.21) |
| **Dynamometry – right arm (average KG)** | **Mean (SD)** | Active=9.4 (9)<br>Inactive=10.5 (9.1) | Active=9.4 (8.6)<br>Inactive=9.6 (8.8) | Active=11.9 (6.6)<br>Inactive=10.3 (9.1) | Active=11.9 (9)<br>Inactive=9.6 (12.2) | Active=12.5 (12.9)<br>Inactive=11.1 (9.1) |
| | **Cohen's d** | -0.12 (-1.09, 0.85) | -0.02 (-0.99, 0.95) | 0.19 (-0.99, 1.37) | 0.21 (-0.97, 1.39) | 0.13 (-0.95, 1.2) |
| **PHQ-15** | **Mean (SD)** | Active=15.4 (3.3)<br>Inactive=13.5 (6) | | Active=15.7 (4.4)<br>Inactive=14.2 (7.2) | | Active=15.2 (5.3)<br>Inactive=12.4 (6) |
| | **Cohen's d** | -0.39 (-1.31, 0.54) | | -0.26 (-1.38, 0.85) | | -0.5 (-1.48, 0.49) |
| **PHQ-9** | **Mean (SD)** | Active=15 (5.2)<br>Inactive=14.1 (8.9) | | Active=13.3 (2.2)<br>Inactive=12.8 (8.4) | | Active=14.3 (6.1)<br>Inactive=12.3 (11.2) |
| | **Cohen's d** | -0.13 (-1.04, 0.79) | | -0.1 (-1.21, 1.01) | | -0.22 (-1.19, 0.75) |
| **GAD-7** | **Mean (SD)** | Active=8.7 (5.6)<br>Inactive=10.5 (7.7) | | Active=7.3 (3.4)<br>Inactive=7.5 (7) | | Active=7.1 (4.9)<br>Inactive=9.1 (7.6) |
| | **Cohen's d** | 0.28 (-0.64, 1.2) | | 0.03 (-1.07, 1.14) | | 0.32 (-0.66, 1.29) |

For peer review only

| | | Visit 1 | | Visit 2 | | Follow-up |
|---|---|---|---|---|---|---|
| **Measure** | **Statistic** | **Pre-TMS** | **Post-TMS** | **Pre-TMS** | **Post-TMS** | |
| **CORE-10** | **Mean (SD)** | Active=18.4 (8.3)<br>Inactive=17.1 (10.3) | | Active=16.7 (4)<br>Inactive=16.5 (9.4) | | Active=14.8 (5.2)<br>Inactive=16.4 (8.2) |
| | **Cohen's d** | -0.14 (-1.06, 0.77) | | -0.03 (-1.13, 1.08) | | 0.24 (-0.73, 1.21) |
| **SF-36 Physical functioning** | **Mean (SD)** | Active=10 (11.5)<br>Inactive=22.7 (22.2) | | Active=15.8 (21.3)<br>Inactive=30 (28.9) | | Active=21.2 (26.4)<br>Inactive=28 (29.6) |
| | **Cohen's d** | 0.73 (-0.21, 1.68) | | 0.58 (-0.55, 1.71) | | 0.24 (-0.73, 1.22) |
| **SF-36 Physical role** | **Mean (SD)** | Active=2.5 (7.9)<br>Inactive=15 (33.7) | | Active=4.2 (10.2)<br>Inactive=20 (36.9) | | Active=8.3 (25)<br>Inactive=17.5 (37.4) |
| | **Cohen's d** | 0.51 (-0.44, 1.46) | | 0.67 (-0.46, 1.81) | | 0.29 (-0.68, 1.27) |
| **SF-36 Bodily pain** | **Mean (SD)** | Active=22.2 (18.3)<br>Inactive=25 (27.1) | | Active=29.8 (27.7)<br>Inactive=19.1 (22.1) | | Active=31 (23.6)<br>Inactive=32.6 (21) |
| | **Cohen's d** | 0.12 (-0.79, 1.04) | | -0.42 (-1.53, 0.7) | | 0.07 (-0.9, 1.04) |
| **SF-36 General health** | **Mean (SD)** | Active=29.9 (9.7)<br>Inactive=30.8 (21.2) | | Active=38.2 (15.8)<br>Inactive=35.4 (26.2) | | Active=31.6 (11)<br>Inactive=39.8 (20.2) |
| | **Cohen's d** | 0.06 (-0.86, 0.97) | | -0.14 (-1.25, 0.97) | | 0.51 (-0.47, 1.5) |
| **SF-36 Vitality** | **Mean (SD)** | Active=17.5 (11.6)<br>Inactive=22.9 (24.6) | | Active=20 (8.4)<br>Inactive=26.5 (25.6) | | Active=29.4 (12.6)<br>Inactive=30.5 (30.1) |
| | **Cohen's d** | 0.28 (-0.63, 1.2) | | 0.39 (-0.73, 1.51) | | 0.05 (-0.92, 1.02) |
| **SF-36 Social functioning** | **Mean (SD)** | Active=20 (17.9)<br>Inactive=28.4 (29.1) | | Active=39.6 (31)<br>Inactive=42.5 (35) | | Active=20.8 (25.8)<br>Inactive=40 (33.7) |
| | **Cohen's d** | 0.35 (-0.57, 1.27) | | 0.09 (-1.02, 1.2) | | 0.64 (-0.35, 1.64) |

For peer review only

| | | Visit 1 | | Visit 2 | | Follow-up |
|---|---|---|---|---|---|---|
| **Measure** | **Statistic** | **Pre-TMS** | **Post-TMS** | **Pre-TMS** | **Post-TMS** | |
| **SF-36 Emotional role** | **Mean (SD)** | Active=12.5 (24.8)<br>Inactive=46.7 (50.2) | | Active=25 (41.8)<br>Inactive=33.3 (41.6) | | Active=59.3 (40.1)<br>Inactive=30 (48.3) |
| | **Cohen's d** | 0.9 (-0.16, 1.95) | | 0.2 (-0.91, 1.31) | | -0.66 (-1.66, 0.33) |
| **SF-36 Mental health** | **Mean (SD)** | Active=54.4 (20.8)<br>Inactive=54.5 (30) | | Active=56 (14.8)<br>Inactive=56.8 (29.7) | | Active=59.6 (18.4)<br>Inactive=59.6 (25.6) |
| | **Cohen's d** | 0.01 (-0.91, 0.92) | | 0.03 (-1.08, 1.14) | | 0 (-0.97, 0.97) |
| **Barthel** | **Mean (SD)** | Active=12.3 (3.8)<br>Inactive=14.5 (5.6) | | Active=12.5 (4.4)<br>Inactive=14.4 (5.6) | | Active=14.9 (4.2)<br>Inactive=15.8 (5.2) |
| | **Cohen's d** | 0.44 (-0.48, 1.37) | | 0.36 (-0.75, 1.48) | | 0.19 (-0.75, 1.14) |
| **WSAS** | **Mean (SD)** | Active=32.3 (3.4)<br>Inactive=29.1 (9.1) | | Active=29.7 (8.3)<br>Inactive=23.9 (10.6) | | Active=29.9 (9.9)<br>Inactive=23.2 (11.8) |
| | **Cohen's d** | -0.48 (-1.4, 0.45) | | -0.63 (-1.76, 0.5) | | -0.62 (-1.61, 0.37) |

**Key:** CORE=10=Clinical Outcomes in Routine Evaluation-10 item; GAD-7=Generalised Anxiety Disorder-7 item; IQR=interquartile range; KG=kilogram; PHQ=Patient Health Questionnaire; SD=standard deviation; SF-36=Short Form Health Survey-36 item; TMS=transcranial magnetic stimulation; WSAS=Work & Social Adjustment Scale

For peer review only

# CONSORT 2010 checklist of information to include when reporting a randomised trial*

| Section/Topic | Item No | Checklist item | Reported on page No |
|---|---|---|---|
| **Title and abstract** | | | |
| | 1a | Identification as a randomised trial in the title | 1 |
| | 1b | Structured summary of trial design, methods, results, and conclusions (for specific guidance see CONSORT for abstracts) | 2 |
| **Introduction** | | | |
| Background and objectives | 2a | Scientific background and explanation of rationale | 4-5 |
| | 2b | Specific objectives or hypotheses | 5 |
| **Methods** | | | |
| Trial design | 3a | Description of trial design (such as parallel, factorial) including allocation ratio | 6 |
| | 3b | Important changes to methods after trial commencement (such as eligibility criteria), with reasons | 11 |
| Participants | 4a | Eligibility criteria for participants | 6-7 |
| | 4b | Settings and locations where the data were collected | 6 |
| Interventions | 5 | The interventions for each group with sufficient details to allow replication, including how and when they were actually administered | 8-11 |
| Outcomes | 6a | Completely defined pre-specified primary and secondary outcome measures, including how and when they were assessed | 11-12 |
| | 6b | Any changes to trial outcomes after the trial commenced, with reasons | NA |
| Sample size | 7a | How sample size was determined | 13-14 |
| | 7b | When applicable, explanation of any interim analyses and stopping guidelines | NA |
| Randomisation: | | | |
| Sequence generation | 8a | Method used to generate the random allocation sequence | 12 |
| | 8b | Type of randomisation; details of any restriction (such as blocking and block size) | 12 |
| Allocation concealment mechanism | 9 | Mechanism used to implement the random allocation sequence (such as sequentially numbered containers), describing any steps taken to conceal the sequence until interventions were assigned | 12 |
| Implementation | 10 | Who generated the random allocation sequence, who enrolled participants, and who assigned participants to interventions | |
| Blinding | 11a | If done, who was blinded after assignment to interventions (for example, participants, care providers, those | 12-13 |



For peer review only - http://bmjopen.bmj.com/site/about/guidelines.xhtml

| | | | |
|---|---|---|---|
| | | assessing outcomes) and how | |
| | 11b | If relevant, description of the similarity of interventions | 8-11 |
| Statistical methods | 12a | Statistical methods used to compare groups for primary and secondary outcomes | 14-15 |
| | 12b | Methods for additional analyses, such as subgroup analyses and adjusted analyses | NA |
| **Results** | | | |
| Participant flow (a diagram is strongly recommended) | 13a | For each group, the numbers of participants who were randomly assigned, received intended treatment, and were analysed for the primary outcome | 18-19 (Fig 1) |
| | 13b | For each group, losses and exclusions after randomisation, together with reasons | 18-19 (Fig 1) |
| Recruitment | 14a | Dates defining the periods of recruitment and follow-up | 6, 19 |
| | 14b | Why the trial ended or was stopped | 18 |
| Baseline data | 15 | A table showing baseline demographic and clinical characteristics for each group | 17-18 |
| Numbers analysed | 16 | For each group, number of participants (denominator) included in each analysis and whether the analysis was by original assigned groups | 18-19 (Fig 1) |
| Outcomes and estimation | 17a | For each primary and secondary outcome, results for each group, and the estimated effect size and its precision (such as 95% confidence interval) | 21-22 (Fig 2) (Supplementary File 4) |
| | 17b | For binary outcomes, presentation of both absolute and relative effect sizes is recommended | NA |
| Ancillary analyses | 18 | Results of any other analyses performed, including subgroup analyses and adjusted analyses, distinguishing pre-specified from exploratory | NA |
| Harms | 19 | All important harms or unintended effects in each group (for specific guidance see CONSORT for harms) | 20 |
| **Discussion** | | | |
| Limitations | 20 | Trial limitations, addressing sources of potential bias, imprecision, and, if relevant, multiplicity of analyses | 26-27 |
| Generalisability | 21 | Generalisability (external validity, applicability) of the trial findings | 26-27 |
| Interpretation | 22 | Interpretation consistent with results, balancing benefits and harms, and considering other relevant evidence | 22-26 |
| **Other information** | | | |
| Registration | 23 | Registration number and name of trial registry | 3 |
| Protocol | 24 | Where the full trial protocol can be accessed, if available | NA |
| Funding | 25 | Sources of funding and other support (such as supply of drugs), role of funders | 28 |

*We strongly recommend reading this statement in conjunction with the CONSORT 2010 Explanation and Elaboration for important clarifications on all the items. If relevant, we also recommend reading CONSORT extensions for cluster randomised trials, non-inferiority and equivalence trials, non-pharmacological treatments, herbal interventions, and pragmatic trials. Additional extensions are forthcoming: for those and for up to date references relevant to this checklist, see www.consort-statement.org.


For peer review only - http://bmjopen.bmj.com/site/about/guidelines.xhtml
For peer review only
